# Site-Specific Labeling of Endogenous Proteins Using CoLDR Chemistry

**DOI:** 10.1021/jacs.1c06167

**Published:** 2021-11-24

**Authors:** Rambabu N. Reddi, Adi Rogel, Efrat Resnick, Ronen Gabizon, Pragati Kishore Prasad, Neta Gurwicz, Haim Barr, Ziv Shulman, Nir London

**Affiliations:** † Department of Chemical and Structural Biology, The Weizmann Institute of Science, Rehovot, 7610001, Israel; ‡ Department of Immunology, The Weizmann Institute of Science, Rehovot, 7610001, Israel; § Wohl Institute for Drug Discovery of the Nancy and Stephen Grand Israel National Center for Personalized Medicine, The Weizmann Institute of Science, Rehovot, 7610001, Israel

## Abstract

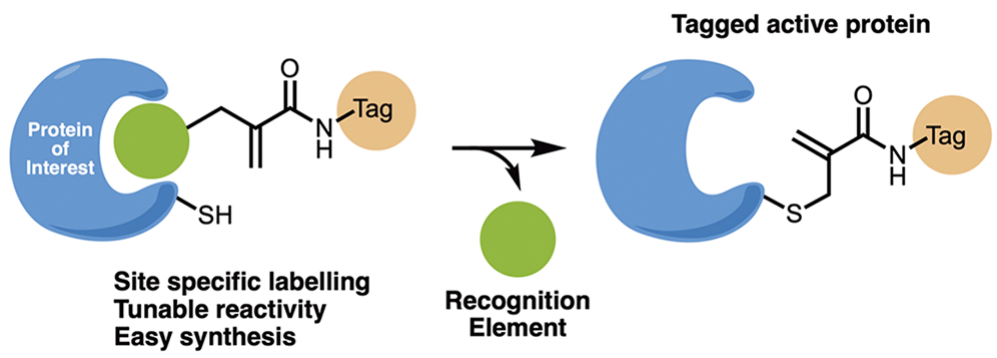

Chemical modifications of native proteins can affect their stability, activity, interactions, localization, and more. However, there are few nongenetic methods for the installation of chemical modifications at a specific protein site in cells. Here we report a covalent ligand directed release (CoLDR) site-specific labeling strategy, which enables the installation of a variety of functional tags on a target protein while releasing the directing ligand. Using this approach, we were able to label various proteins such as BTK, K-Ras^G12C^, and SARS-CoV-2 PL^pro^ with different tags. For BTK we have shown selective labeling in cells of both alkyne and fluorophores tags. Protein labeling by traditional affinity methods often inhibits protein activity since the directing ligand permanently occupies the target binding pocket. We have shown that using CoLDR chemistry, modification of BTK by these probes in cells preserves its activity. We demonstrated several applications for this approach including determining the half-life of BTK in its native environment with minimal perturbation, as well as quantification of BTK degradation by a noncovalent proteolysis targeting chimera (PROTAC) by in-gel fluorescence. Using an environment-sensitive “turn-on” fluorescent probe, we were able to monitor ligand binding to the active site of BTK. Finally, we have demonstrated efficient CoLDR-based BTK PROTACs (DC_50_ < 100 nM), which installed a CRBN binder onto BTK. This approach joins very few available labeling strategies that maintain the target protein activity and thus makes an important addition to the toolbox of chemical biology.

## Introduction

Selective modifications of native proteins in cells with chemical probes are a powerful tool to tune and investigate protein function, conformation, structure, cellular signaling, localization, and more. Fluorescent labeling of a protein of interest (POI) is a prominent example that can enable imaging, analysis of the structure, function, dynamics, and localization of a target protein.^1,2^ Other modifications can control the stability,^3^ activity,^4^ and localization^5^ of a target protein.

Genetic engineering methods allow the introduction of a fluorescent domain^6−8^ or a chemically reactive domain,^9−12^ which enables selective labeling of exogenously expressed proteins. These approaches, however, typically rely on overexpressed proteins, and the newly introduced domains can be large and perturb the very same process they aim to investigate.^13−15^ Genetic code expansion enables site-specific incorporation of unnatural amino acids bearing bioorthogonal reactive handles.^16,17^ The subsequent bio-orthogonal reaction with a suitable complementary reactive functionality allows effective and selective bioconjugation. This circumvents the introduction of a large domain, but these methods are laborious and require specifically engineered cells,^16^ limiting their scope.

An alternative to genetic methods is chemical bioconjugation. Several chemical reactions for modifying naturally occurring amino acids while elegantly controlling the selectivity of the probes have been developed for *in vitro* protein labeling and allowed the generation of well-defined biotherapeutics and post-translational modification mimics.^17−24^

In order to selectively label endogenous proteins even in the crowded environment of live cells, various molecules comprising a target recognition moiety, a reactive functionality, and a probe moiety (or tag) were developed.^25−28^ In these cases, the protein targeted by traditional affinity labeling often loses its native activity since the recognition moiety permanently occupies its ligand-binding pocket. This may hinder the investigation of protein involvement in relevant cellular processes.

Over the past decade, Hamachi et al. have pioneered ligand-directed chemistries, which include ligand-directed, tosyl (LDT),^29^ acyl imidazole (LDAI),^30^ bromo benzoate (LDBB),^31^ sulfonylpyridine,^32^ and *N*-acyl-*N*-alkyl sulfonamide (LDNASA)^33^ chemistries. In these bioconjugation methods, the ligand leaves the active site after forming a covalent bond with a nucleophilic residue on the POI.^34^ Although these methods enabled prominent applications and could retain target protein activity,^35,36^ some challenges remain. First, the size of the required activating groups and/or linkers is substantial and precludes the labeling of residues very close to the active site. Second, the nucleophile itself is not rationally selected. It is empirically discovered what residue ends up reacting with the probe; therefore it is hard to assess which target would be amenable to the chemistry. Lastly, some of these chemistries suffer from slow kinetics, low stability in the cellular environment, and structural complexity. Hence, there is a need to develop new ligand-directed chemistries using simple and small reactive groups to reach the desired location and specifically label particular nucleophilic amino acids.

**Figure 1:**
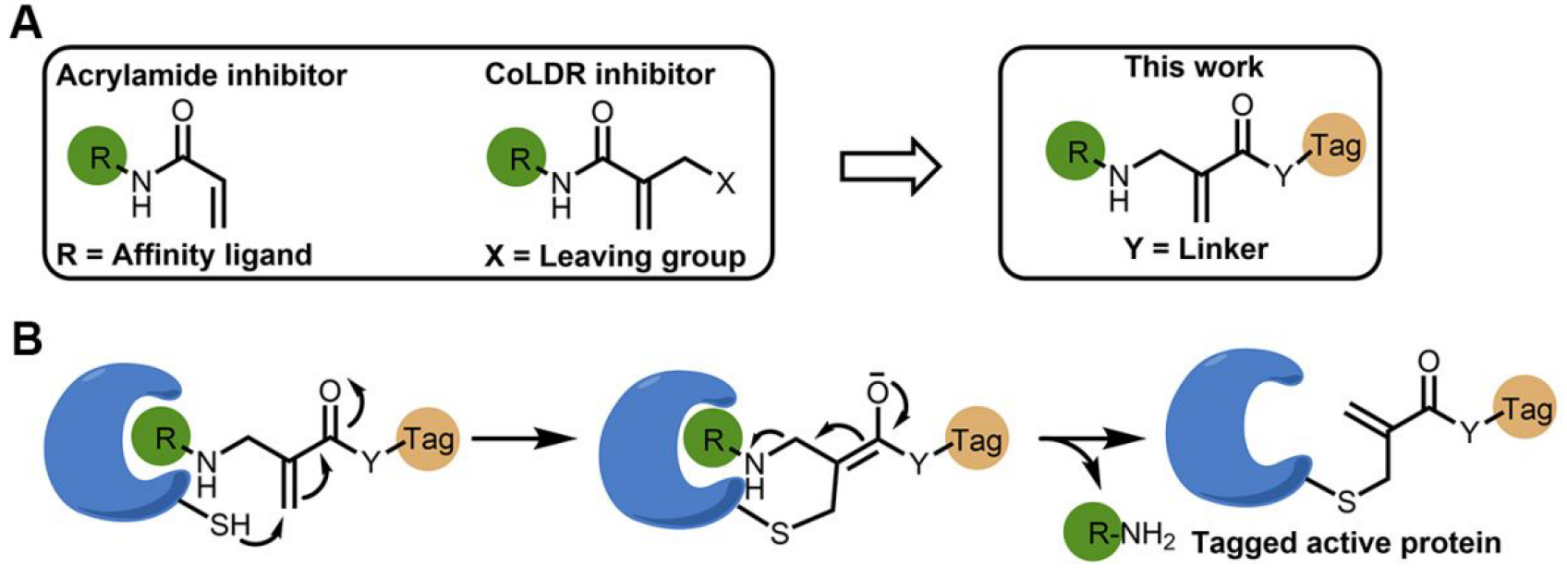
Development of ligand-directed cysteine labeling probes. (A) By reversing the directionality of our previously developed CoLDR chemistry,^37^ we generate probes that place the electrophilic carbon in the exact same position but now release the protein recognition moiety (R; typically an inhibitor). (B) Schematic representation of the reaction of a target cysteine with a substituted α-methacrylamide through CoLDR chemistry.

Acrylamides are one of the few electrophiles that meet the criteria for successful covalent “warheads” to be incorporated into drugs (Figure S1). Recently, we described α-substituted methacrylamides, which upon reaction with thiol nucleophiles, undergo a conjugated addition–elimination reaction, ultimately releasing the substituent at the α′ position.^37^ These compounds have been used as targeted covalent inhibitors, and covalent ligand directed release (CoLDR) chemistry was demonstrated for “turn-on” fluorescence and chemiluminescence probes (Figure 1A). Several amines, phenols, carboxylic acids, and carbamates successfully underwent elimination after a reaction with a thiol group. In this regard, we envisioned that reversing the directionality of the acrylamide—placing the protein-targeting moiety (recognition element) as the substituent at the α’ position—can lead to the elimination of the ligand (typically an inhibitor) after reaction with the target cysteine. This can be used for site-specific cysteine labeling at the protein active site with various tags (Figure 1B).

Here we explore CoLDR chemistry in the context of site-specific labeling of endogenous proteins *in vitro* and in cells with fluorescent and alkyne tags. Importantly we show that labeling a near active-site cysteine residue in BTK does not inhibit its activity. We used these probes to determine the half-life of BTK and demonstrated that labeling BTK with a CRBN binder efficiently degraded the protein. Since this approach allows irreversible tagging of the protein while maintaining its activity, we envision it will find many uses for novel protein proximity inducers.

## Results

### Site-Specific Labeling Probes for BTK

**Figure 2:**
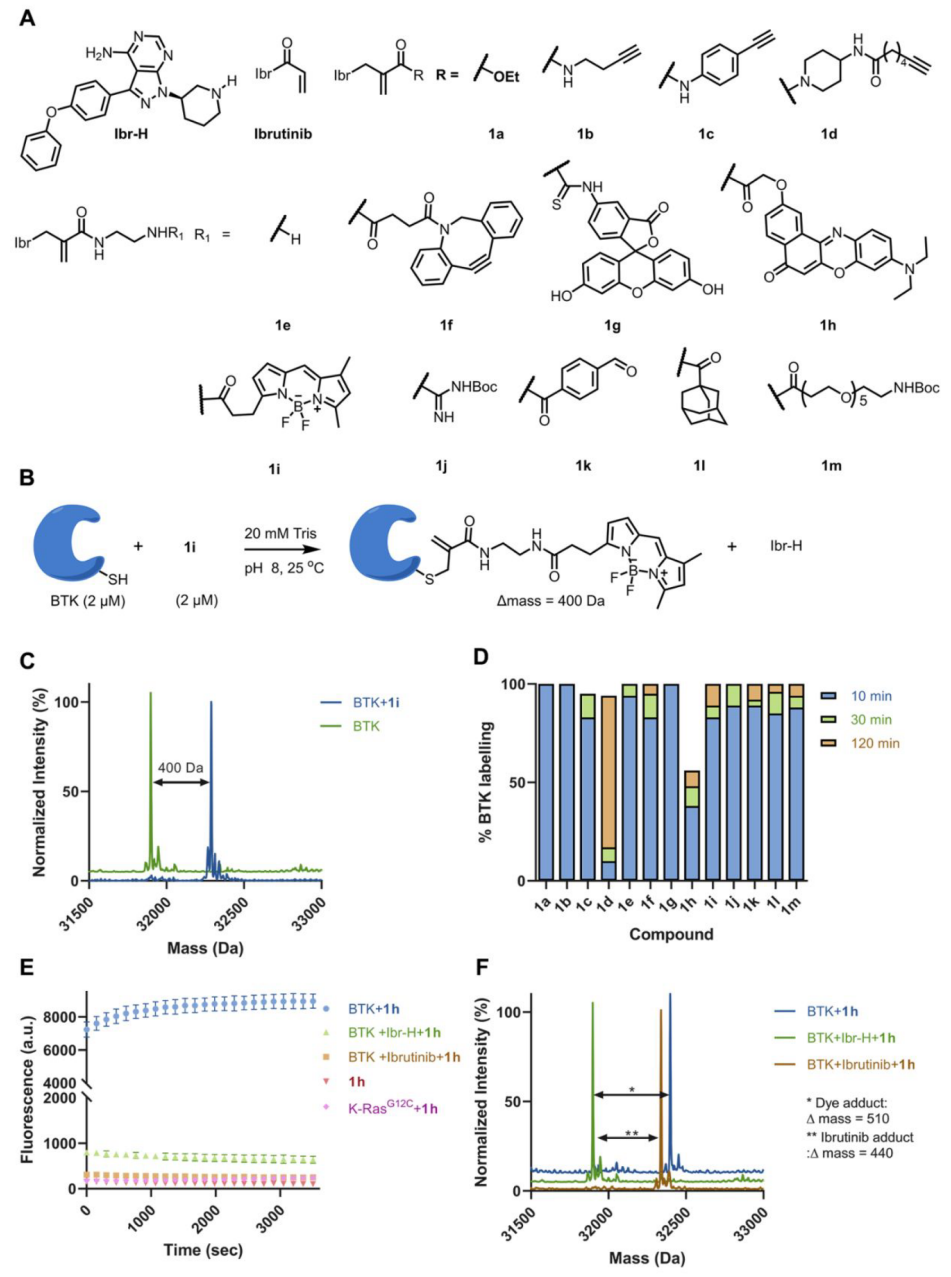
Site-selective labeling of BTK using CoLDR chemistry. (A) Chemical structures of ibrutinib-directed methacrylamides with various functional tags. (B) Typical example of the reaction of BTK (2 μM) with **1i** (2 μM) in a 20 mM Tris buffer at pH 8, 25 °C. (C) Deconvoluted LC/MS spectrum shows the labeling of a BODIPY probe and demonstrates Ibr-H leaving. (D) Percent of labeling of BTK (2 μM) with the probes (**1a**–**1m**; 2 μM) at 10, 30, and 120 min in 20 mM Tris buffer at pH 8, 25 °C. (E) Kinetics of the increase in fluorescence intensity measured at Ex/Em = 550/620 nm (*n* = 4) upon addition of BTK (2 μM) to **1h** (2 μM) in 20 mM Tris buffer at pH 8, 37 °C (blue). Control experiments without BTK (red), preincubation of ibrutinib (4 μM) and Ibr-H (4 μM) prior to adding **1h** (green and orange, respectively), and incubation of K-Ras^G12C^ (pink) with **1h** show no fluorescence. (F) Deconvoluted LC/MS spectra for BTK incubated with **1h** at the end of the fluorescence measurement (shown in E). The adduct mass corresponds to a labeling event in which the Ibr-H moiety was released, validating the proposed mechanism.

We chose Bruton’s tyrosine kinase (BTK), an established drug target for B-cell malignancies, as a model protein for ligand-directed site-selective labeling. Ibrutinib, which is a highly potent covalent inhibitor of BTK that binds at its ATP-binding pocket, was used as the ligand to guide the selective labeling of BTK’s noncatalytic cysteine 481.^38^ The amine precursor for ibrutinib (Ibr-H; Figure 2) contains a piperidine moiety, which can be installed as a heterosubstituent on an α-methacrylamide and thus serve as a leaving group.^37^ We designed and synthesized substituted methacrylamide ibrutinib analogues (Figure 2A; Figure S2) which contain various functional tags such as “click” chemistry handles: alkyne (**1b**, **1c**, and **1d**) and dibenzyl cyclooctyne (DBCO; **1f**), fluorescent dyes (**1g**, **1h**, **1i**), hydrophobic tags (**1l**, **1m**), and derivatives of natural amino acid side chains (**1a**, **1e**, **1j**, **1k**). The synthesis of these probes is straightforward by treating Ibr-H with bromomethacrylic acid to give a convenient acid that could be further functionalized (Figure S2).

To assess irreversible labeling and validate the proposed ligand release mechanism, we incubated our probes (2 μM) with recombinant BTK (2 μM) and monitored the reaction via intact protein liquid chromatography/mass spectrometry (LC/MS). For example, analysis of the reaction with **1i** (Figure 2B) verified that the shift in mass corresponds to labeling BTK with BODIPY and release of Ibr-H (Figure 2C). All of the tested probes labeled BTK to >95% within 10–120 min except **1h** (pH 8, 25 °C; Figure 2D), with an adduct mass corresponding to the probe without ligand (Figure S3). To verify the site-specificity, we analyzed BTK incubated with either DMSO or **1b** followed by trypsin digestion and analysis of the tryptic peptides by LC/MS/MS. Cys481 was identified as the site of modification both through MS/MS identification of the **1b**-modified tryptic peptide (residues 467–487, Figure S4; Data set S1) and by depletion of iodoacetamide-labeled 467–487 peptide upon reaction with **1b**.

To assess the kinetic parameters of labeling, we performed a time-dependent incubation experiment of BTK (200 nM) with various concentrations of **1b** (300–2000 nM, 20 mM Tris, pH 8, 14 °C; Figure S5A), resulting in *k*_inact_ = 2.78 × 10^–2^ s^–1^ and *K*_i_ = 3.0 × 10^–7^ M under these conditions (Figure S5B). These values are similar to previously reported values for ibrutinib^39^ (*k*_inact_ = 2.70 × 10^–2^ s^–1^; *K*_i_ = 5.42 × 10^–8^ M; *k*_inact_/*K*_i_ = 4.98 × 10^5^), where the reversible binding component is about 5-fold weaker for **1b** and *k*_inact_ is similar.

To validate that the binding site of BTK remains vacant following labeling by **1b**, we have performed surface plasmon resonance (SPR) experiments. We conjugated to the SPR chip a reversible analogue of ibrutinib through a long PEG linker (**1r**; Figure S6A). We then flowed either free BTK (Figure S6B), **1b**-labeled BTK (following irreversible labeling we removed excess **1b** and Ibr-H via dialysis, see Methods; Figure S6C), or ibrutinib-labeled BTK (Figure S6D) at various concentrations over the chip. Free BTK (*K*_D_ = 15 nM) and BTK**-1b** (*K*_D_ = 18 nM) bind **1r** with high affinity (Figure S6E), whereas BTK-ibrutinib does not show any binding. This indicates that the labeling of BTK with CoLDR probes does not affect the binding of other reversible ligands. We should note that slightly less overall binding (*R*_max_) was detected with BTK-**1b**, which is probably due to the presence of a small amount of reversible Ibr-H that was not removed during dialysis.

Next we assessed the stability of BTK labeled with a CoLDR probe in the presence of reduced glutathione (GSH). We incubated BTK (2 μM) with **1i** (2 μM; 30 min; pH 8; 25 °C). The BTK-**1i** conjugate was then further incubated with GSH (1 or 5 mM; 18 h; pH 8; 25 °C; Figure S7A). After 18 h, no detachment of the probe from BTK or addition of GSH was observed (Figure S7B), indicating the stability of this modification to conditions similar to the cellular environment.

Solvatochromic fluorophores possess emission properties that are sensitive to the nature of the local microenvironment, which is exploited to study protein structural dynamics and the detection of protein-binding interactions.^40^ Recently it was shown that proximity-induced binding of solvatochromic or torsionally responsive fluorophores to a nonspecific protein surface in the vicinity of the prove's binding site can result in “turn-on” fluorescence.^41,42^ However, the presence of bound ligands can impose significant structural changes on the structure of proteins. Compound **1h**, which has an environmentally sensitive fluorogenic probe, allowed us to develop a turn-on fluorescent probe for BTK in its apo form.

**1h** has negligible fluorescence in and of itself (Ex/Em = 550/620 nm; Figure 2E). However, upon the addition of BTK (pH 8, 37 °C), the fluorescence intensity of **1h** at 550 nm increased 80-fold within seconds, reaching saturation within 5 min (Figure 2E). Such fast labeling compared to the results reported in Figure 2D may be the result of the higher temperature at which this experiment was performed. Intact protein LC/MS following the fluorescence measurement showed the expected adduct mass of the fluorophore without the ibrutinib recognition element, validating covalent binding and the proposed mechanism (Figure 2F). Preincubation with either ibrutinib or the noncovalent analogue of ibrutinib (Ibr-H) eliminated the fluorescence, indicating that it requires binding at the active site of BTK. Further, the LC/MS chromatogram of these control reactions showed no labeling of **1h** in the presence of competitors (Figure 2F). To assess the selectivity of the probe, we incubated it with an alternative covalent target, K-Ras^G12C^, which did not elicit fluorescence (Figure 2E). We could assess the initial rate of fluorescence generation, by reducing the concentration of the reactants **1h** (50 nM) with BTK (1 μM) at 30 °C (Figure S8A).

We wished to test whether we can use **1h** to detect binding events within the active site of BTK. After labeling BTK with **1h**, we incubated the adduct with Ibr-H or with ibrutinib. This resulted in a 2–3-fold decrease of fluorescence, as well as a significant red shift of the emission from 620 to 650 nm (Figure S8B,C). These results indicate that BTK retains the ability to bind the ligands in the active site after being labeled. The change in fluorescence may be due to conformational changes of BTK or in the positioning of the fluorescent probe after binding, resulting in an altered chemical environment.^43,44^ Spectral changes were also observed with BTK prelabeled with **1i** and **1g** (Figure S8D,E).

We followed these spectral changes in a small screen of BTK active-site binders we have previously identified.^37^ We have incubated 180 compounds with **1h**-labeled BTK and recorded the fluorescence spectra (Data set S2). Interestingly, many compounds shifted the fluorescence spectrum peak from 620 to 650 and/or quenched the fluorescence (see the most pronounced changes in Figure S8F,G). Several of the compounds with the most pronounced effects are kinase inhibitors, some of which were previously reported to inhibit BTK (Data set S2).

### Intrinsic Thiol Reactivity of BTK Probes

To explore the intrinsic thiol reactivity of these BTK labeling probes, we reacted **1a**–**1m** with GSH (5 mM; phosphate buffer pH 8) as a model thiol and monitored the reaction over time via LC/MS (Figure S9A). As an example, analysis of the reaction of **1i** at time 0 and 8 h (Figure S9B) clearly indicates the formation of a substitution product, the release of Ibr-H, and the decrease of starting material. The rates of the release of Ibr-H, formation of the GSH adduct, and depletion of **1i** are identical (Figure S9D), suggesting the release of ligand (Ibr-H) is concomitant with the reaction with GSH. Further, to compare the reactivity of these probes with ibrutinib, we measured the GSH half-life (*t*_1/2_) upon incubation with all compounds (Figure S9C, D, and E). Almost all probes show a reactivity within a 2-fold range of ibrutinib. Most molecules are slightly more reactive than ibrutinib, with a few notable exceptions. Compounds **1h** and **1d** are about 2- and >20-fold less reactive, respectively, whereas the ester-based **1a** is significantly more reactive (*t*_1/2_ < 10 min). It is interesting to compare compounds **1b**–**1d**, which differ in the nature of the acrylamide amine. The simple primary amine and aniline show moderate reactivity (*t*_1/2_ = 30 min to 4 h) toward GSH, whereas **1d**, with a piperidine moiety, shows *t*_1/2_ > 100 h. This variation in reactivity may help tune the selectivity of these probes. Note that **1h** and **1d**, with the least reactivity toward GSH, also showed lower labeling of BTK (Figure 2D).

We should note that none of the compounds show decomposition under the GSH reaction conditions (Figure S10). Further, we have checked the buffer stability of these compounds in phosphate buffer at pH 8, 37 °C for 4 days and found no significant decomposition except for **1a**, **1e**, and **1g** (Figure S11). Compounds **1e** and **1g** underwent 25% and 5% Ibr-H elimination, respectively, after 4 days, whereas **1a** underwent both hydrolysis (50%) and elimination (50%) in 2 days.

### CoLDR Labeling Is General across Protein Targets

**Figure 3:**
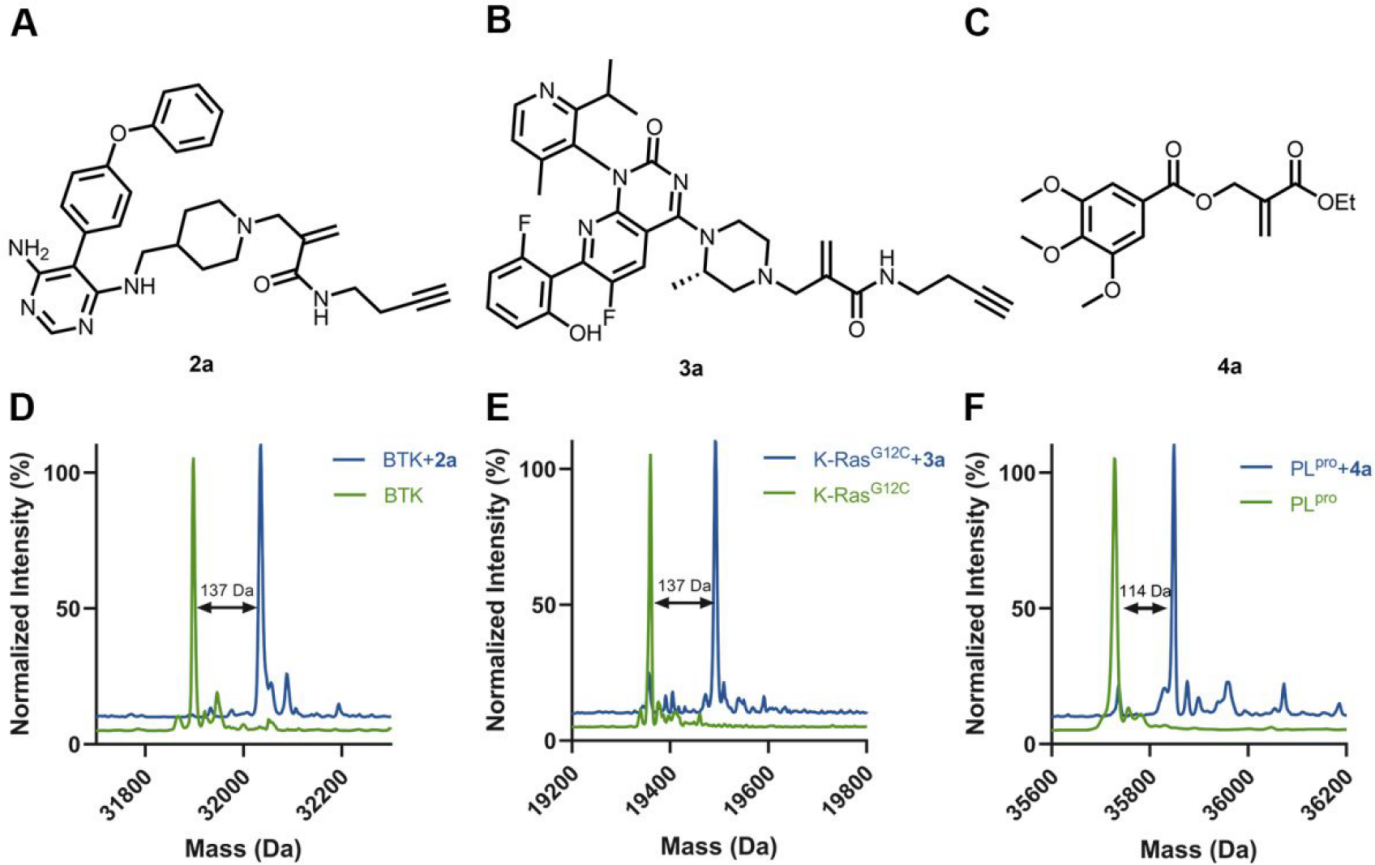
Selective labeling of various target proteins. Structures of alkyne/ester labeling probes for **(**A) BTK, (B) K-Ras^G12C^, and (C) SARS-CoV-2 PL^pro^. Deconvoluted LC/MS spectra for (D) BTK (2 μM) incubated with **2a** (2 μM) in 20 mM Tris buffer at pH 8, 25 °C, 10 min, (E) K-Ras^G12C^ (10 μM) incubated with **3a** (100 μM) in 20 mM Tris at pH 8, 37 °C, 16 h, and (F) PL^pro^ (2 μM) incubated with **4a** (10 μM) in 50 mM Tris at pH 8, 25 °C, 16 h. The adduct masses correspond to a labeling event in which the ligand was released.

To show the generality of this approach, we chose another ligand of BTK, evobrutinib, as well as two other therapeutic targets for which covalent inhibitors were available, K-Ras^G12C^ and the SARS-CoV-2 papain-like protease (PL^pro^), as model systems. We have synthesized an evobrutinib-based alkyne probe (**2a**; Figures 3A and S12) and an AMG-510-based alkyne probe to target K-Ras^G12C^ (**3a**; Figure 3B) and an ethyl-acrylate labeling ligand (**4a**; Figure 3C) for PL^pro^ based on a covalent ligand we have previously identified (Figure S1). The probes were incubated with their targets (BTK: 2 μM, 10 min, 25 °C; KRas^G12C^: 10 μM, 16 h, 37 °C; PL^pro^: 2 μM, 16 h, 37 °C; all reactions performed at pH 8). All three probes were able to reach 100% single labeling of their target as assessed by LC/MS (Figure 3D–F) with the adduct masses corresponding to the alkynes (BTK and KRas^G12C^) or ethyl acrylate (PL^pro^). We should note that in the case of PL^pro^, since the cysteine target is the catalytic residue, we expect this modification to also inhibit the enzyme.

### Ligand-Directed Site-Selective Labeling of BTK in Cells

**Figure 4:**
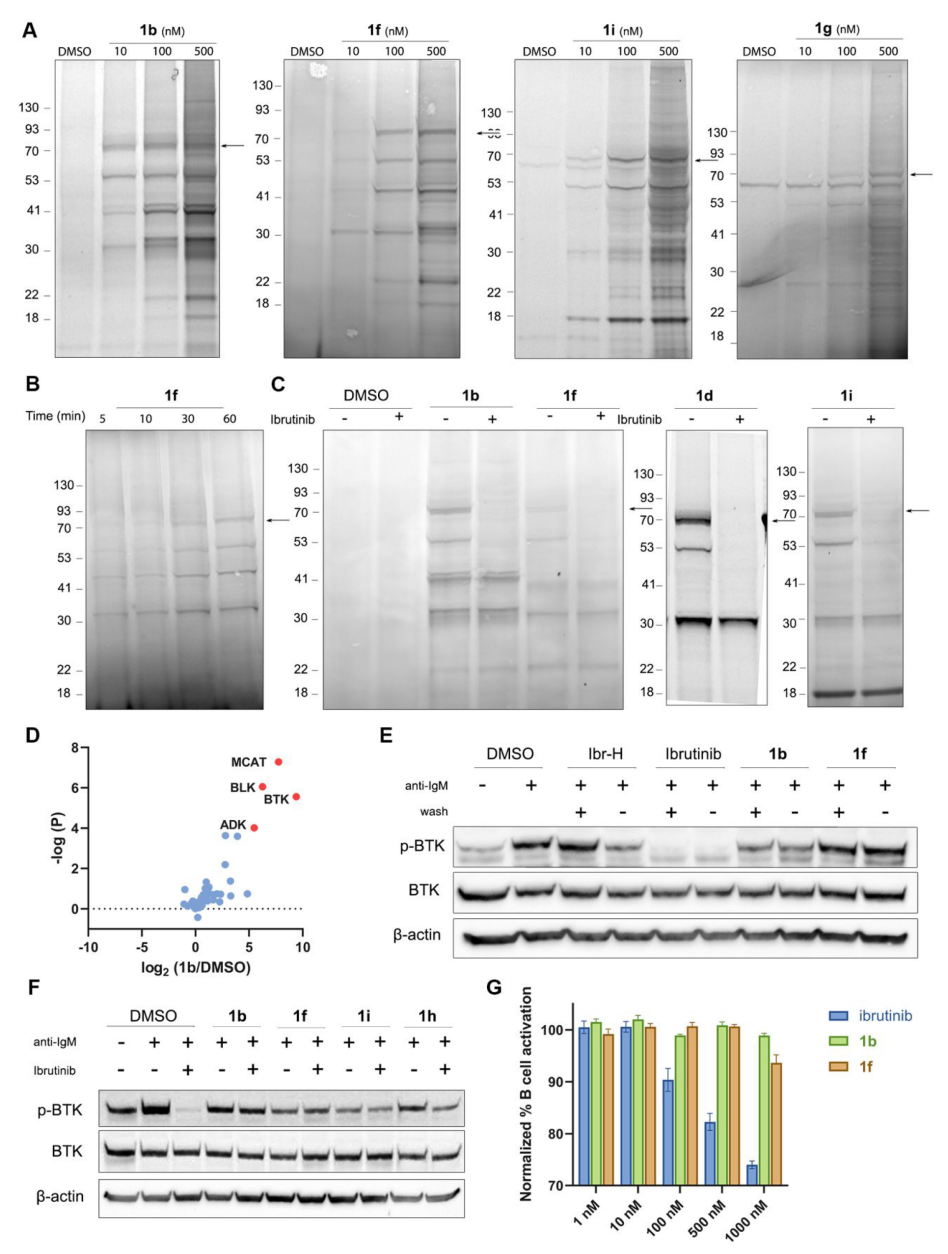
Labeling BTK with CoLDR probes does not inhibit its activity in cells. (A) Cellular labeling profile of **1b**, **1f**, and **1i** after 2 h of incubation with Mino cells and **1g** in Mino cell lysate. **1b** and **1f** samples were further reacted with TAMRA-azide in lysate before imaging. An arrow indicates BTK’s MW. (B) Time-dependent labeling profile of **1f** with BTK after incubation of Mino cells with 100 nM probe followed by a click reaction with TAMRA-azide in lysate prior to imaging. (C) Competition experiment of **1b**, **1d**, **1f**, and **1i** with ibrutinib. The cells were preincubated for 30 min with either 0.1% DMSO or 1 μM ibrutinib, followed by 2 h of incubation with 200 nM **1b** or **1f** or 100 nM **1d** or **1i**. (D) Mino cells were incubated with 0.1% DMSO or **1b** (100 nM). Samples were further reacted with biotin-azide in lysate, followed by enrichment, trypsin digestion, and peptide identification by LC/MS/MS. The log(fold-ratio) of proteins enriched by **1b** over DMSO is plotted as a function of statistical significance. BTK is clearly identified as the most enriched target; additional prominent targets that correspond to bands identified by in-gel fluorescence (panel C) are indicated. (E) BTK activity assay in Mino cells as measured by autophosphorylation of BTK. The cells were incubated for 1 h with either 0.1% DMSO, 1 μM ibrutinib, 1 μM Ibr-H, or 100 nM **1b**, **1f**, **1h**, or **1i**. The cells were either washed or not before induction of BTK activity by anti-IgM. (F) BTK activity assay: Mino cells were incubated for 2 h with either DMSO or 1 μM **1b**, **1f**, **1i**, and **1h**, washed, and then incubated for 45 min with ibrutinib (100 nM). The cells were washed again before induction of BTK activity by anti-IgM. The CoLDR probes were able to rescue BTK activity from inhibition by ibrutinib. (G) Primary B cell activation induced by anti-IgM after 24 h of treatment with increasing doses of either ibrutinib, **1b**, or **1f**, showing no inhibition of the CoLDR probes.

In addition to the *in vitro* labeling of BTK by our probes, we also tested their engagement in cells and their proteomic selectivity. We incubated Mino B cells with probes containing different tags, such as an alkyne (**1b**, **1c**, and **1d**), dibenzocyclooctyne (DBCO; **1f**), and the fluorescent dyes fluorescein (**1g**), Nile red (**1h**), and BODIPY (**1i**) and used in-gel fluorescence (following Cu-catalyzed cycloaddition (CuAAC) of TAMRA-N_3_ to the alkyne tags) to image their labeling profiles. Probes **1b** and **1i** showed robust labeling even at a concentration of 10 nM (Figure 4A), whereas **1d** labeled BTK with more selectivity (Figure 4C, Figure S13A). **1f**- and **1h**-labeled BTK at a concentration of 100 nM (Figure 4A and Figure S13B) and **1g** did not label BTK in live cells. Negatively charged fluorophores such as fluorescein have known permeability issues. Indeed, in lysate **1g** was able to label BTK at a concentration of 100 nM (Figure 4A). To assess the kinetics of the cellular labeling, we followed the time-dependent labeling by **1f**, which showed robust labeling of BTK within 30–60 min (Figure 4B).

To validate the molecular target of the probes, we performed a competition experiment, where we preincubated the cells with ibrutinib prior to labeling with our probes (Figure 4C, S13A, and S13B). This experiment confirmed BTK labeling as ibrutinib completely competed for the labeling of the band at ∼70 kDa, as well as some of the off-targets. It is interesting to note that some off-targets did not compete with ibrutinib, indicating these are new off-targets specific to our probes (Figure 4C). To identify the off-targets of these probes, we performed a pull-down proteomics experiment in Mino cells (Figure 4D) using **1b**. Cells were treated with either DMSO or **1b** (100 nM) or pretreated with ibrutinib and then with **1b**. Biotin-azide was conjugated to the alkyne via CuAAC, and avidin beads were used for enrichment. We have found BLK, MCAT, and ADK as off-targets for probe **1b** (Figure 4D; Data set S3). ADK (40.5 kDa) and MCAT (also known as SLC25A20; 33 kDa) correspond to the two bands seen in the gel (Figure 4C) that are not competed by ibrutinib. Both are abundant proteins in the cell, which may explain probe binding. Overall very few off-targets were detected for all probes at the lower concentration, similar to a previously reported ibrutinib-alkyne probe.^45^

### BTK Labeling Preserves Its Enzymatic Activity

In order to examine the effect of BTK modification by these probes on its cellular activity, we performed activity assays in both Mino and primary B cells. Mino cells were incubated (1 h) with probes **1b**, **1f**, **1h**, and **1i** followed by BTK activation using anti-human IgM. BTK autophosphorylation was followed by Western blot to assess its activity. While ibrutinib completely abolished BTK autophosphorylation, BTK remained active after labeling with all four probes. **1f**, **1h**, and **1i**, in particular, did not affect the activity (Figure 4E and Figure S14). This effect was indifferent to washing of the cells, which abolished the inhibition of the BTK reversible inhibitor Ibr-H, but not that of ibrutinib (Figure 4E). Further, to ensure that the activity did not originate from unlabeled BTK, Mino cells were treated with high concentrations of **1b**, **1f, 1i**, and **1h** (1 μM) for 2 h and then incubated with 100 nM ibrutinib for 45 min before activation with IgM. While ibrutinib alone completely inhibited BTK’s activity, we show that all CoLDR probes can rescue this inhibition. Compounds **1i** and **1h** do show some reduction in phosphorylation upon ibrutinib incubation, indicating incomplete BTK labeling in cells. When we increase the concentration of **1h** (5 μM; 4 h incubation; Figure S14), adding ibrutinib no longer reduces the activity. The fact that BTK’s activity remains intact suggests that the labeled fraction remains active (Figure 4F and Figure S14). In addition, we measured the effect of these probes on B cell receptor (BCR) signaling in primary mouse B cells. Mouse splenic cells were isolated and treated for 24 h with a dose–response of ibrutinib, **1b**, and **1f**, and B cell activation in response to stimulation with anti-IgM was measured by following the expression of CD86. In contrast to ibrutinib, both probes did not inhibit the activation of B cells, suggesting they not only preserve BTK autophosphorylation but also do not interfere with its downstream signaling (Figure 4G).

### BTK Half-Life Determination Using CoLDR Probes

**Figure 5:**
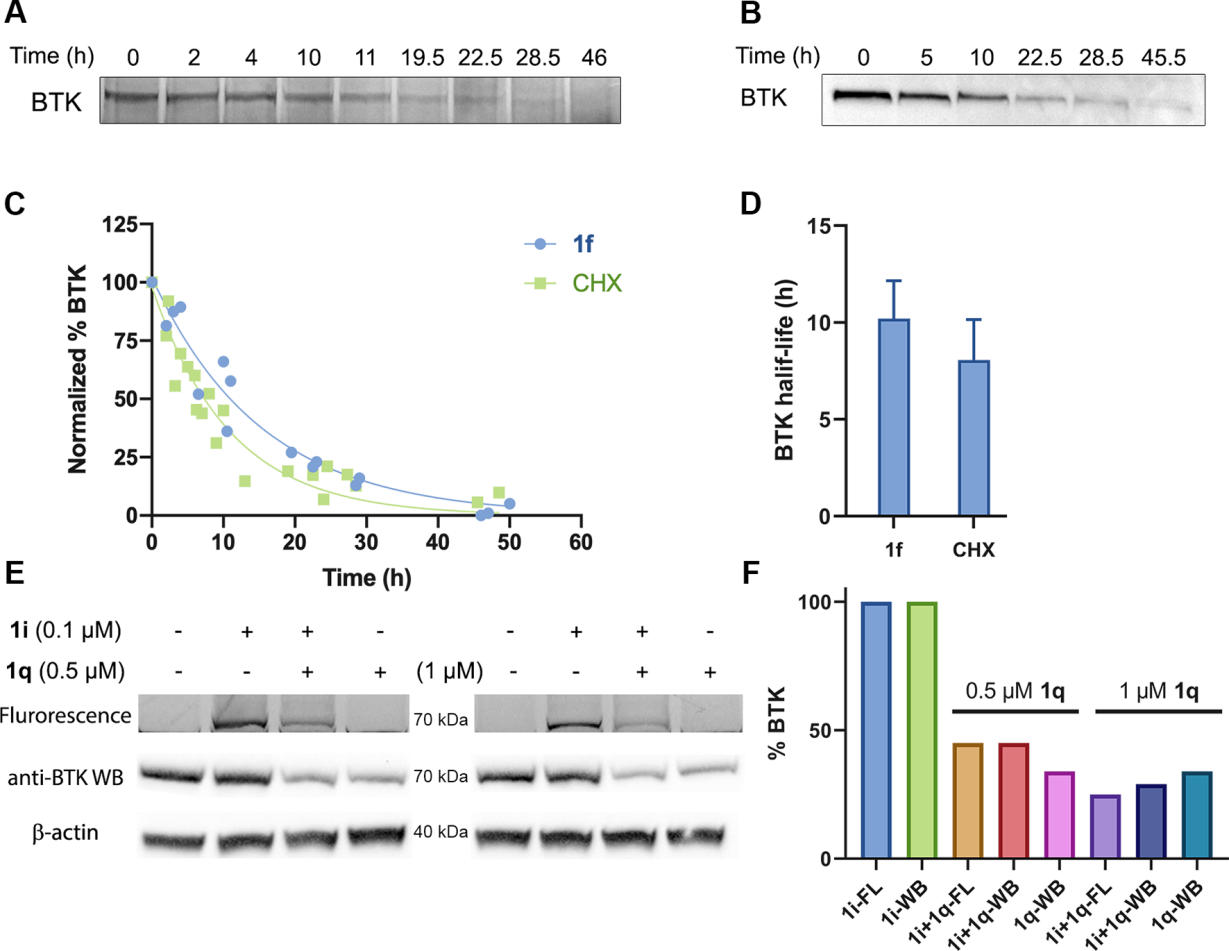
Measurement of BTK half-life. (A) Half-life measurement of BTK using **1f**. Mino cells were pulse-labeled with 100 nM **1f** for 1 h and were then washed to remove the excess probe. Cells were harvested at the indicated time points, and lysates were reacted with TAMRA-azide. The signal of BTK was quantified, and the half-life was calculated. (B) Half-life measurement of BTK with the cycloheximide (CHX) assay, using 20 μg/mL cycloheximide. (C) Quantification of BTK levels in A and B (by normalization to the protein concentration) in Mino cells (**1f**: *n* = 3, CHX: *n* = 4). (D) Calculated half-life by both methods, presented as mean ± SD. (E) Degradation of BTK labeled with **1i** using PROTAC **1q**. Mino cells were incubated with **1i** (100 nM), then washed to remove the excess probe, again incubated with PROTAC **1q** for 2 h at 0.5 and 1 μM, and then lysed. Samples are subjected to in-gel fluorescence (FL) and Western blot (WB). (F) Quantification of BTK levels in panel E (normalization to the β-actin has been done for Western blot).

As we have shown that labeling by **1f** does not inhibit the enzymatic activity and downstream signaling of BTK, we wanted to use this probe to measure the half-life of BTK in the native cellular environment. For this purpose, we incubated Mino cells for 1 h with **1f** to label BTK, followed by washing to ensure that newly synthesized BTK will not be labeled. Cells were then harvested at different time points, and the lysates were “clicked” using a Cu-free reaction by the addition of TAMRA-azide. We followed BTK abundance by in-gel fluorescence, which allowed quantification and half-life determination (Figure 5A). The average half-life of BTK measured with **1f** was 10.2 ± 2.0 h, which is similar to its half-life measured with the traditional cycloheximide (CHX) assay (Figure 5B, C, and D), but did not require an antibody or Western blotting and importantly did not perturb the cell translation machinery. We should note the loss of **1f** signal is due to a decrease in BTK protein levels and not, for example, probe decomposition, since several **1f** off-targets exhibited much longer half-lives, indicating the probe is stable over these time scales (Figure S15).

### BTK Tagging Does Not Interfere with PROTAC Binding and Ternary Complex Formation

Proteolysis targeting chimeras (PROTACs) are a popular modality to induce selective degradation of cellular proteins.^46−48^ We^49^ and others^50−57^ have previously reported both covalent and noncovalent PROTACs for BTK. We have shown that tagging BTK with an alkyne allowed us to follow its natural degradation in the cell. Now, we were curious to see if we can follow induced targeted degradation by a BTK PROTAC (Figure S16A). To do so, we incubated Mino cells with fluorescent probe **1i** (100 nM) for 1 h, then washed the cells, incubated them with a noncovalent BTK PROTAC **1q**(49) (Figure S16B) for 2 h, and measured BTK degradation using both in-gel fluorescence (Figures 5E and S16C) and Western blotting (Figure S16D). Interestingly, degradation of BTK quantified by gel fluorescence (75% at 1 μM, 55% at 0.5 μM) closely corresponds to the quantification by the Western blot (71% at 1 μM, 55% at 0.5 μM). This suggests the PROTAC-mediated degradation can be followed using in-gel fluorescence by prelabeling the target with a CoLDR fluorescent tag. Importantly, in the absence of **1i**, PROTAC **1q** degraded 65% of the protein at 0.5 and 1 μM. Both are similar to the degradation by **1q** in the presence of the fluorescence tag. Almost no degradation has been observed at lower concentrations of **1q** (50 and 100 nM) in both the presence and absence of **1i** (Figure S16E,F). Altogether these data suggest the fluorescent tag does not interfere with the binding of a noncovalent PROTAC nor with the formation of a ternary complex with CRBN E3 ligase.

### CoLDR Chemistry Allows the Installation of a Degradation Handle

**Figure 6:**
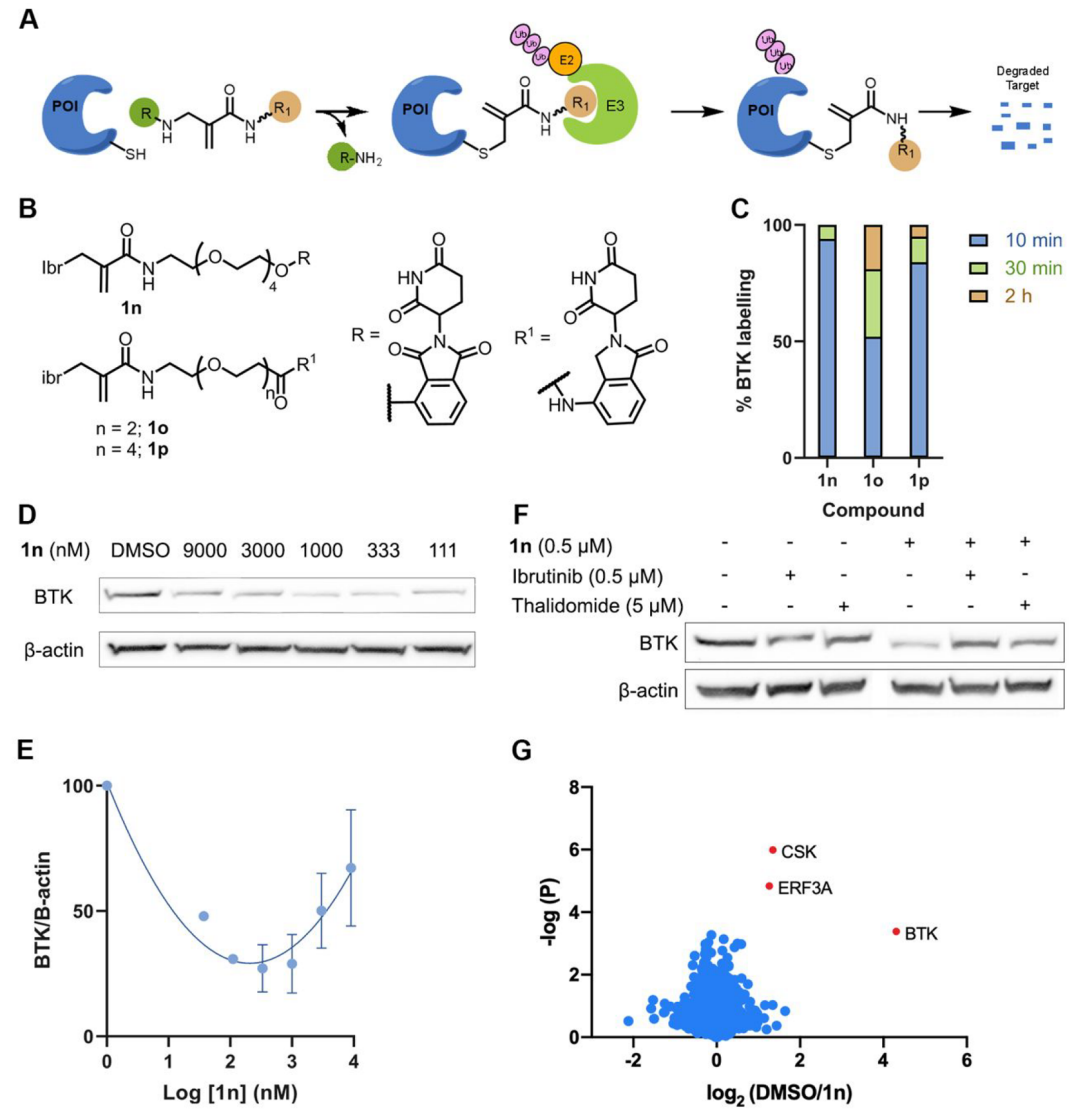
Measurement of induced degradation by CoLDR PROTACs. (A) Schematic representation of target degradation using CoLDR PROTACS. (B) Structure of CoLDR-based BTK PROTACS. (C) *In vitro* labeling of BTK (2 μM) with **1n**–**1p** (2 μM) in 20 mM Tris buffer at pH 8, 25 °C. (D) Western blot evaluation of BTK levels in Mino cells in response to various concentrations of **1n** after 24 h of incubation. (E) Quantification of BTK levels in D by normalization to the β-actin house-keeping gene in Mino cells. DC_50_ and *D*_max_ were calculated by fitting the data to a second-order polynomial using the Prism software. (F) Mino cells were pretreated for 2 h with either ibrutinib/thalidomide-OH or DMSO before treatment with a BTK PROTAC for 24 h (*n* = 2). Subsequently, BTK levels were measured via Western blot. (G) Mino cells were treated for 24 h with either 0.1% DMSO or **1n** (500 nM) in 4 replicates. Lysates were subjected to trypsin digestion and peptide identification by LC/MS/MS. The Log_2_(fold-ratio) of proteins enriched in the DMSO samples over **1n-**treated samples is plotted as a function of statistical significance. Significantly degraded proteins are indicated in red and defined as Log_2_(DMSO/**1n**) > 1 and *p*-value < 0.01.

Small-molecule binders are known to thermodynamically stabilize their target proteins,^58−61^ which may also translate to improved cellular stability to degradation.^62^ We wanted to assess whether the fact that we can bind BTK in its apo form will allow better degradation, even via single-turnover covalent PROTACs (Figure 6A).

We have designed three CoLDR PROTACs that utilize Ibr-H as a leaving group, to install a CRBN binder (thalidomide/lenalidomide) through a PEG linker onto BTK (Figure 6B). The synthesis of these compounds is straightforward, by coupling thalidomide/lenalidomide PEG amine with Ibr-carboxylic acid (Figure S17). We first assessed BTK labeling by these PROTACs (2 μM BTK, 2 μM PROTAC; pH 8, 25 °C). All three PROTACs labeled BTK by more than 80% within 30 min (Figure 6C, Figure S18). We then assessed if they can induce BTK degradation in Mino cells. **1n** proved to be the best degrader, with a DC_50_ < 100 nM (11.4 nM according to the polynomial fit; Figure 6D,E and Figure S19). To validate the degradation mechanism of **1n**, we pretreated Mino cells with either ibrutinib or thalidomide-OH before incubation with the PROTAC. Both were able to rescue the degradation, suggesting it is mediated by binding to BTK and to CRBN (Figure 6F).

Finally, we assessed the proteomic selectivity of **1n** by quantitative label-free proteomics (Figure 6G; Data set S4). Out of the proteins identified and quantified in both DMSO and **1n**-treated samples, only three proteins were depleted by more than 50% with a *p*-value < 0.01. The most prominent target was BTK, which was depleted more than 16-fold. A prominent off-target we observed was CSK, a noncovalent off-target of ibrutinib, which was depleted a little more than 50%. However, depletion of CSK was small relative to values observed for other BTK PROTACs that engaged their target purely noncovalently,^49^ indicating that covalent binding plays an important role in target recruitment. The second major off-target, Erf3A (also known as GSPT1), is a known target for IMiD-CRBN binders.^63−65^ None of the off-targets enriched by **1b** (Figure 4D) were detected as a degradation target of **1n**. Very few proteins were identified and quantified only in one set of the samples (Data set S4B), precluding their quantification. Three proteins were observed in DMSO-treated samples but were not detected in the **1n**-treated samples, among them the prominent ibrutinib off-target BLK.

## Discussion

In this work, we show how a reversal of the directionality of our previously reported CoLDR chemistry^37^ allows the site-specific cellular labeling of a native protein of interest while sparing its enzymatic activity.

The use of this chemistry offers several advantages for protein labeling reagents. The chemistry is straightforward (Figure 1) and can theoretically be applied to any reported acrylamide inhibitor, as we showed for two BTK inhibitors and a K-Ras^G12C^ inhibitor (Figure S1). The fact that we do not change the position of the electrophilic carbon minimizes the risk of interfering with covalent bond formation to the target. It also means that we know a priori which residue will be labeled with the newly installed tag (Figure S4) versus random labeling that is observed when using alternative chemistries.^66^ It is interesting to note that the parent PL^pro^ inhibitor is a chloroacetamide (Figure S1), suggesting that this strategy is not limited to α,β-unsaturated carbonyl binders. We should also note that nothing precludes adding a linker between the reversible recognition element and the electrophile to access target residues that are further away from the binding site.

We have shown that tags with a wide variety of functionalities could be installed (Figure 2A), indicating that the approach is versatile. It is interesting to note that in our previous study^37^ piperidine-substituted methacrylamides only partially reacted through an addition–elimination mechanism (40%) when binding both BTK and GSH, whereas here, all of the tested ibrutinib CoLDR analogues fully eliminated the Ibr-H ligand, despite its linkage through a piperidine moiety, when binding to both BTK and GSH. This suggests that the nature of the acrylamide in addition to the nature of the leaving group influences the propensity for addition–elimination versus traditional Michael addition; this can be tuned and would require further investigation.

We demonstrate a few applications for this labeling platform both *in vitro* and in cells. The installation of an environment-sensitive “turn-on” fluorescent probe for instance has advantages over our previously reported CoLDR fluorescent “turn-on” probes^37^ since now, in addition to the generation of fluorescence upon binding, we label the active protein and the dye can serve as a reporter for binding events in the protein (Figure S8) and perhaps for its conformation. Previous reports showed that environmentally sensitive probes could be useful to explore binding events to a target protein.^43,44^ The fact that our probes do not hinder binding to the active site can facilitate investigation of alternative ligand binding events. We should also note the fluorescence signal amplification with this probe was higher than what we previously reported (80-fold vs 30-fold) and its wavelength red-shifted (650 nm vs 435 nm), but these are likely consequences of fluorophore selection, rather than leaving group chemistry.

In cells, fluorescent labeling of apo-BTK enabled us to determine its half-life in a much more convenient and straightforward manner (Figure 5A), without perturbing the cellular translation machinery. While for this particular target the half-life corresponds well between the two methods (Figure 5B), it may well be that for other targets treatment with CHX distorts the real protein half-life. The quantification of targeted degradation of BTK using in-gel fluorescence (Figure 5E,F) is another alternative technique for antibody-based Western blot to measure targeted degradation of proteins. With improved specificity this may translate to a high-throughput method for evaluation of protein degraders.

The described CoLDR PROTACs (Figure 6) displayed potent degradation compared to previously reported irreversible BTK PROTACs,^50,57,67−69^ although we cannot rule out that they work through a reversible binding mechanism, as was shown in the past for other acrylamide-based BTK PROTACs.^49^ We should note that a recent attempt to use ligand-directed NASA chemistry with a similar concept was unsuccessful at degrading CDK2,^70^ which suggests that such degradation is likely protein and site dependent. These compounds also offer the interesting property of attenuating the half-life of BTK, while keeping it active, which may be useful for biological investigation of its function. More generally, we believe that by screening various tags to install on a protein target we could perhaps tune its half-life without affecting its activity. Such an application could be useful both for biological research and for instance to increase the half-lives of tumor suppressor proteins, as potential therapeutics.

Our approach also comes, of course, with several limitations. First, the generality of our approach depends, of course, on the availability of a selective and potent binder. This limits its scope compared to genetic approaches.

A potential liability is the fact that the released recognition element may in fact still bind reversibly to a target active site and show some inhibition. However, since the concentration of the released moiety can at most reach the concentration of the target protein in the cell, which is typically very low, we think this is not a prominent obstacle, as is demonstrated for Ibr-H (Figure 4E).

Another limitation is that this approach is only applicable to target cysteine residues, which are among the rarest amino acids in the proteome. Whereas previously reported labeling chemistries demonstrated targeting of various additional amino acids,^29−33^ out of about 200 cysteines in and around active sites of kinases, a cautious estimation based on manual inspection suggests that 64 are amenable to tagging with our approach while still retaining activity.^71,72^ In a PDB wide screen (over all proteins), we have recently identified ∼11 000 cysteines proximal to a ligand.^73^ If a similar proportion is located such that tagging will spare enzymatic activity, there are still thousands of opportunities for application of this approach. Finally, our strategy will only preserve the activity of the protein when targeting a noncatalytic cysteine residue.

Some of our probes showed increased reactivity compared to the parent ibrutinib. However, the increased reactivity did not translate to pronounced promiscuity in cells, as we showed that all probes at low concentrations label their target protein with very few off-targets (Figure 5A). Moreover, the intrinsic thiol reactivity of the resulting probes seems to be tunable quite significantly based on both the amine moiety and the CoLDR tag (Figure S9).

Notwithstanding these limitations, our approach now allows for a new generation of protein proximity inducers. While we demonstrated its application toward protein degradation, by installing an E3 ligase recruiter, it has not escaped our notice that due to the sparing of the enzymatic activity of the target, the approach could be used for various protein proximity applications, such as phosphorylation-inducing chimeras (PHICs^74^), by recruiting new substrates to a tagged active kinase or in general recruiting new targets for any active enzyme. These applications are the subject of ongoing research.

In summary, we present a new platform for site-specific labeling of proteins that is compatible with cellular conditions and spares the labeled protein’s activity. This approach joins very few such available strategies and thus makes an important addition to the toolbox of chemical biology.

## Methods

### LC/MS Measurements

LC/MS runs were performed on a Waters ACQUITY UPLC class H instrument in positive ion mode using electrospray ionization. UPLC separation for small molecules used a C18-CSH column of 1.7 μm, 2.1 mm × 100 mm, for all the LC/MS-based assays. The column was held at 40 °C, and the autosampler at 10 °C. Mobile phase A was 0.1% formic acid in water, and mobile phase B was 0.1% formic acid in acetonitrile. The run flow was 0.3 mL/min. The gradient used was 100% A for 2 min, increasing linearly to 90% B for 5 min, holding at 90% B for 1 min, changing to 0% B in 0.1 min, and holding at 0% for 1.9 min. UPLC separation for proteins used a C4 column (300 Å, 1.7 μm, 2.1 mm × 100 mm). The column was held at 40 °C, and the autosampler at 14 °C. Mobile solution A was 0.1% formic acid in water, and mobile phase B was 0.1% formic acid in acetonitrile. The run flow was 0.4 mL/min with a gradient of 20% B for 2 min, increasing linearly to 60% B for 3 min, holding at 60% B for 1.5 min, changing to 0% B in 0.1 min, and holding at 0% for 1.4 min (for the kinetic labeling experiment, the gradient used was 90% A for 0.5 min, 90–40% A for 0.50–2.30 min, 40–10% A for 2.60–3.20 min, 10% A for 0.2 min, 10–90% A for another 0.2 min, and 90% A for 0.6 min). The mass data were collected on a Waters SQD2 detector with an *m*/*z* range of 2–3071.98 at a range of *m*/*z* of 800–1500 Da for BTK and 750–1550 for K-RAS^G12C^.

### Labeling Experiments of Ibrutinib Derivatives with BTK

BTK kinase domain was expressed and purified as previously reported.^49^ Binding experiments were performed in 20 mM Tris pH 8.0 and 50 mM NaCl at room temperature. The BTK kinase domain was diluted to 2 μM in the buffer, and 2 μM ibrutinib derivatives (**1a**–**1p**, **2a**) were added by adding 1/100th volume from a 200 μM solution. The reaction mixtures, at room temperature for various times, were injected into the LC/MS. For data analysis, the raw spectra were deconvoluted using a 20 000:40 000 Da window and 1 Da resolution. The labeling percentage for a compound was determined as the labeling of a specific compound (alone or together with other compounds) divided by the overall detected protein species. For K-Ras^G12C^, 10 μM protein was incubated with 100 μM compound **3a** in 20 mM Tris pH 8.0 and 50 mM NaCl at 37 °C for 16 h. For PL^pro^, 2 μM protein was incubated with 10 μM **4a** in 300 mM NaCl, 50 mM Tris pH 8, and 1 mM TCEP at 25 °C for 16 h.

### Plate Reader Fluorescence Measurements

Plate reader measurements were performed on Tecan Spark Control 10M using black 384-well plates with clear bottoms. Excitation was measured with a 550 ± 35 nm filter and emission with a 620 ± 30 nm filter.

#### Fluorescence Intensity Measurements with **1h**

The BTK kinase domain was diluted to 2 μM in the buffer, and 2 μM **1h** was added by adding 1/100th volume from a 200 μM solution. Control measurements were performed without protein and BTK with preincubation with 4 μM Ibr-H/ibrutinib for 5 min. Each condition was done in quadruplicate in 20 mM Tris pH 8.0 and 50 mM NaCl for BTK. Fluorescent measurements were taken every 2 min for 1 h for BTK/K-Ras^G12C^. At the end of the measurements, samples were injected directly into the LC/MS for labeling quantification.

#### High-Throughput Screening with **1h**

High-throughput screening was performed with the Selleck compound collection at 200 μM for the initial screen in 384-well black plates (Thermo Fisher Scientific-Nunclon 384 Flat Black [NUN384fb]). The collection was composed of BTK binding compounds obtained in our previous luminescence screen^37^ (Data set S2). BTK (2 μM) was incubated with compound **1h** (4 μM) for 1 h. The resulting BTK-**1h** (50 μL) was added to the inhibitors. The screen was performed with 20 mM Tris pH 8.0 and 50 mM NaCl at 32 °C, and fluorescence was recorded after 10 min.

### GSH Reactivity Assay for Ibrutinib Derivatives

A 100 μM (0.5 μL of a 20 mM stock) sample of the electrophile (**1a**–**1m**) was incubated with 5 mM GSH (5 μL of a 100 mM stock, freshly dissolved), 5 mM NaOH (to counter the acidity imparted by GSH), and 100 μM 4-nitrocyanobenzene (0.5 μL of a 20 mM stock solution) as an internal standard in 100 mM potassium phosphate buffer pH 8.0 and DMF at a ratio of 9:1, respectively. All solvents were bubbled with argon. Reaction mixtures were kept at 14 °C. Every 1 h 5 μL from the reaction mixture was injected into the LC/MS. The reaction was followed by the peak area of the electrophile normalized by the area of the 4-nitrocyanobenzene (i.e., by the disappearance of the starting material). The natural logarithm of the results was fitted to linear regression, and *t*_1/2_ was calculated as *t*_1/2_ = ln 2/–slope.

### Buffer Stability Assay for Model Compounds

A sample of 100 μM of the electrophile (**1a**–**1p**) was incubated with 100 μM 4-nitrocyanobenzene as an internal standard in a 100 mM potassium phosphate buffer of pH 8.0. All solvents were bubbled with argon. Reaction mixtures were kept at 37 °C with shaking. After 4 days (unless otherwise mentioned), 5 μL from the reaction mixture was injected into the LC/MS to check the stability of the compounds.

### In-Gel Fluorescence Labeling Profile

Mino cells were cultured in RPMI medium supplemented with 15% fetal bovine serum and 1% penicillin/streptomycin, at 37 °C and 5% CO_2_. The cells were treated for 2 h with either 0.1% DMSO or the indicated concentrations of **1b**, **1f**, and **1i**. For the competition experiment the cells were preincubated for 30 min with 1 μM ibrutinib followed by 2 h of incubation with 200 nM **1b** and **1f** and 100 nM **1c**, **1d**, **1h**, and **1i**. The cells were lysed with RIPA buffer (Sigma, R0278), and protein concentration was determined using the BCA protein assay (Thermo Fisher Scientific, 23225). Lysates were then diluted to 2 mg/mL in PBS. Incubation with **1g** was performed in lysates for 2 h at 25 °C. Lysates with **1b**, **1c**, **1d**, and **1f** were clicked to TAMRA-azide (Lumiprobe). For **1b**, **1c**, and **1d** the “click” reaction was performed using a final concentration of 40 μM TAMRA-azide, 3 mM CuSO_4_, 3 mM tris(3-hydroxypropyltriazolylmethyl)amine (THPTA, Sigma), and 3.7 mM sodium l-ascorbate (Sigma) in a final volume of 60 μL. For **1f** the “click” reaction was performed by incubation with 40 μM TAMRA-azide. The samples were incubated at 25 °C for 2 h. A 20 μL amount of 4× LDS sample buffer (NuPAGE, Thermo Fisher Scientific, NP0007) was added followed by a 10 min incubation at 70 °C. For samples with **1c** and **1d**, precipitation has been done before the addition of a sample buffer.

#### Precipitation

The 1× chloroform, 4× methanol, and 3× water were added to the samples and vortexed thoroughly. The samples were spun down for 10 min at 4 °C. The top layer was aspirated, and the pellet was resuspended in 4× methanol. The sample was vortexed and spun down again for 10 min at 4 °C, the solution was removed, and the pellet was dried for 2 min. The pellet was dissolved in 42 μL of PBS followed by 14 μL of 4× sample buffer. The samples were then loaded on a 4–20% Bis-Tris gel (SurePAGE, GeneScript) and imaged using a Typhoon FLA 9500 scanner. **1b**, **1c**, **1d**, **1f**, and **1h** were scanned at 532 nm; **1g** and **1i** were scanned at 473 nm.

### Pull-Down Proteomics Experiments

Mino cells were incubated for 1 h with DMSO or ibrutinib followed by the incubation with 100 nM **1b**. The cells were lysed and “clicked” with biotin-azide, and the samples were incubated at room temperature for 1 h. The samples were then precipitated with methanol–chloroform (1 mL of methanol, 250 μL of chloroform, 750 μL of water), washed with 1 mL of methanol, and air-dried. The samples were solubilized, bound to streptavidin agarose beads in PBS for 3 h at 25 °C. The beads were washed, centrifuged, resuspended in Tris 50 mM pH 8, and transferred to a clean Eppendorf tube. After this, the bound proteins were eluted by boiling with 5% SDS then reduced with DTT, alkylated with iodoacetamide, and digested with trypsin. The samples were run on LC/MS/MS. The detailed procedure is available in the Supplementary Methods Section.

### Degradation Proteomic Analysis (Label-Free Quantitative Proteomics)

A total of 1 × 10^6^ Mino cells were treated in four replicates with either **1n** (500 nM) or DMSO (0.1%) for 24 h. Cells were then harvested and washed with ice cold PBS. Samples were then centrifuged at 200 rcf, 4 °C for 5 min; then the supernatant was removed and the samples were frozen at −80 °C. Samples were dispersed in 75 μL of 50 mM ammonium bicarbonate and transferred to 1.8 mL glass vials. A 75 μL amount of 10% SDS in 50 mM ammonium bicarbonate was added, and the samples were heated to 96 °C for 6 min. The samples were sonicated thoroughly in a sonication bath until the DNA was sheared, as indicated by reduction in viscosity to a level enabling easy pipettation. Total protein concentration was estimated using the BCA assay, and 30 μg from each sample was taken for LC/MS/MS analysis. The detailed procedure is available in the Supplementary Methods Section.

### BTK Activity in Cells

Following the indicated cell treatment, BTK phosphorylation was induced with 10 μg/mL anti-human IgM (Jackson ImmunoResearch, 109-006-129) for 10 min at 37 °C. The cells were harvested, and immunoblots of phospho-BTK, total-BTK, and β-actin were performed.

### B-Cell Response Experiment

Splenic cells from C57BL/6 mice were isolated by forcing spleen tissue through the mesh into PBS containing 2% fetal calf serum and 1 mM EDTA, and red blood cells were depleted by lysis buffer. Cells were cultured in 96-well U-bottom dishes (1 × 10^6^ cells/mL in RPMI 10% FCS) and incubated with ibrutinib, **1b**, and **1f** in different concentrations (1, 10, 100, 1000 nM) for 24 h at 37 °C in 5% humidified CO_2_. Following a 24 h incubation, cells were stimulated with anti-IgM overnight (5 μg/mL, Sigma-Aldrich). Subsequently, cells were stained with anti-B220 (clone RA3-6B2, Biolegend) and anti-CD86 (clone GL-1, Biolegend) antibodies (anti-mouse CD86 Biolegend 105008 1:400, anti-mouse/human CD45R/B220 Biolegend 103212 1:400) for 30 min at 4 °C. Single-cell suspensions were analyzed by a flow cytometer (CytoFlex, Beckman Coulter).

### Immunoblotting

Cell pellets were washed with ice-cold PBS and lysed using RIPA buffer (Sigma, R0278). Lysates were clarified at 21000*g* for 15 min at 4 °C, and protein concentration was determined using the BCA protein assay (Thermo Fisher Scientific, 23225). Samples containing 50 μg of total protein were prepared with 4× LDS sample buffer (NuPAGE, Thermo Fischer Scientific, NP0007) and were then resolved on a 4–20% Bis-Tris gel (GeneScript SurePAGE, M00657). Proteins were separated by electrophoresis and were then transferred to a nitrocellulose membrane (Bio-Rad, 1704158) using the Trans-Blot Turbo system (Bio-Rad). The membrane was blocked with 5% BSA in TBS-T (w/v) for 1 h at room temperature, washed four times for 5 min with TBS-T, and incubated with the following primary antibodies: rabbit anti-phospho-BTK (#87141s, Cell-Signaling, 1:1000, overnight at 4 °C), mouse anti-BTK (#56044s, Cell-Signaling, 1:1000, 1 h at room temperature), and mouse anti-β-actin (#3700, Cell-Signaling, 1:1000, 1 h at room temperature). Membrane was washed four times for 5 min with TBS-T (with 0.05% tween) and incubated with the corresponding HRP-linked secondary antibody (mouse #7076/rabbit #7074, Cell-Signaling) for 1 h at room temperature. The EZ-ECL kit (Biological Industries, 20-500-1000) was used to detect HRP activity. The membrane was stripped using Restore stripping buffer (Thermo Fisher Scientific, 21059) after each secondary antibody before blotting with the next one.

### Half-Life Determination

Measurements with **1f** were performed by pulse labeling of BTK in Mino cells with 100 nM **1f** for 1 h, followed by a wash with PBS three times to remove excess probe. The cells were incubated at 37 °C in a 5% humidified CO_2_ incubator and harvested at the indicated time points. Cell pellets were lysed with RIPA buffer and clicked with TAMRA-azide; proteins were separated by electrophoresis and imaged as described in detail in the in-gel fluorescence section. BTK’s bands were quantified using ImageJ software, and BTK levels at time-point zero were defined as 100%.

Measurements with CHX were performed by treating Mino cells with 20 μg/mL CHX. Cells were harvested at the indicated time points for subsequent analysis by immunoblotting of BTK and β-actin. Bands were quantified using ImageJ, BTK signal was normalized to protein concentration, and levels at time-point zero were defined as 100%. For both methods, BTK levels versus time points were plotted and the data were fitted to one-phase decay in Prism 8 to calculate the half-life.

#### Degradation of BTK-Labeled **1i**

Measurements with **1i** were performed by labeling of BTK in Mino cells with 100 nM **1i** for 1 h, followed by a wash with PBS three times to remove excess probes. The cells were incubated again with **1q** (0.5 and 1 μM) for 2 h at 37 °C in a 5% humidified CO_2_ incubator and harvested at the indicated time points. Cell pellets were lysed with RIPA buffer, proteins were separated by electrophoresis, and the gel was fixed using a fixing solution (45% methanol, 45% water, and 10% acetic acid) with 2 × 25 mL immediately. The gel was imaged as described in detail in the in-gel fluorescence section. BTK’s bands were quantified using ImageJ software, and BTK levels at only **1i** defined as 100%. The same samples were also analyzed by immunoblotting of BTK and β-actin. Bands were quantified using ImageJ, BTK signal was normalized to β-actin, and levels in DMSO were defined as 100%.

#### Degradation of BTK by a CoLDR PROTAC

Measurements with **1n**–**1p** were performed by treating Mino cells in various concentrations (9, 3, 1, 0.33, 0.11, 0.036 μM) of the compounds (**1n**–**1p**) and incubated for 24 h. Cells were harvested for subsequent analysis by immunoblotting of BTK and β-actin. Bands were quantified using ImageJ, BTK signal was normalized to β-actin, and levels in DMSO were defined as 100%. BTK levels versus concentrations were plotted, and the data were fitted to one-phase decay in Prism 8 to calculate the DC_50_.

## Supporting Information Available

Additional information including synthesis schemes of all CoLDR-based probes, intact protein mass spectrometry validation of covalent adducts, site labeling identification, determination of kinetic parameters of CoLDR probe **1b**, SPR binding measurements, stability of BTK labeling assessment, fluorescent detection of binding events to BTK, GSH reactivity rates, buffer stability measurements, fully scanned gels for all WB and in-gel fluorescence, and additional supplementary methods section (PDF)
Detailed synthetic protocols for preparation of compounds with high-resolution mass spectrometry and NMR analysis (PDF)
LC/MS/MS and all chemoproteomics results (XLSX)
