# Supplementary material for: Site-Specific Labeling of Endogenous Proteins Using CoLDR Chemistry
Source: J Am Chem Soc. 2021 Nov 24;143(48):20095–108. doi: 10.1021/jacs.1c06167 (PMC8662641; doi:10.1021/jacs.1c06167)
Supplement: Supplementary file 1 — ja1c06167_si_001.pdf [file ja1c06167_si_001.pdf]

# Site-Specific Labelling of Endogenous Proteins Using CoLDR Chemistry

Rambabu N. Reddi[1,*], Adi Rogel[1,*], Efrat Resnick[1], Ronen Gabizon[1], Pragati Kishore Prasad[1], Neta Gurwicz[2], Haim Barr[3], Ziv Shulman[2], Nir London[1,#]

[1] Dept. of Chemical and Structural Biology, The Weizmann Institute of Science, Rehovot, 7610001, Israel.

[2] Dept. of Immunology, The Weizmann Institute of Science, Rehovot, 7610001, Israel.

[3] Wohl Institute for Drug Discovery of the Nancy and Stephen Grand Israel National Center for Personalized Medicine, The Weizmann Institute of Science, Rehovot, 7610001, Israel

[*] Equal contribution

[#] To whom correspondence should be addressed, nir.london@weizmann.ac.il

Keywords: Bioconjugation, covalent inhibitors, CoLDR, protein half-life, PROTAC, Targeted protein degradation, Turn-on fluorescence, Ligand directed chemistry

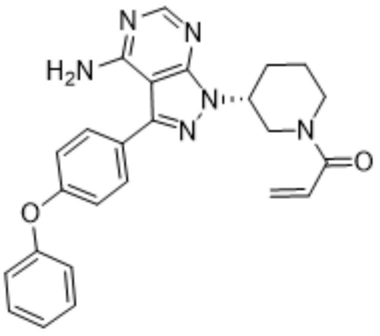

Ibrutinib

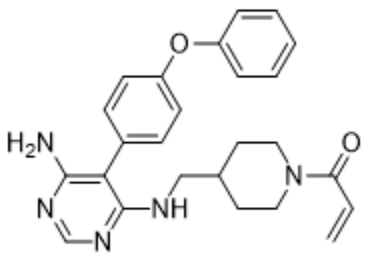

Evobrutinib

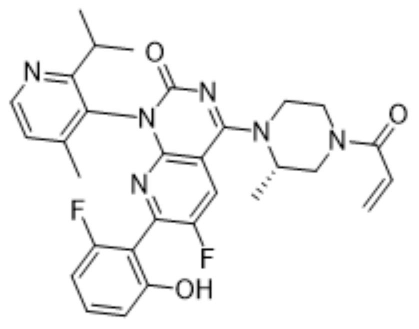

Amg-510

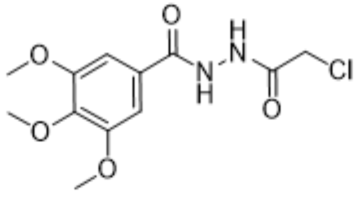

PL pro inhibitor

**Figure S1. Structures of Ibrutinib, Evobrutinib, AMG -510 and a SARS-CoV-2 PL$^{pro}$ inhibitor**

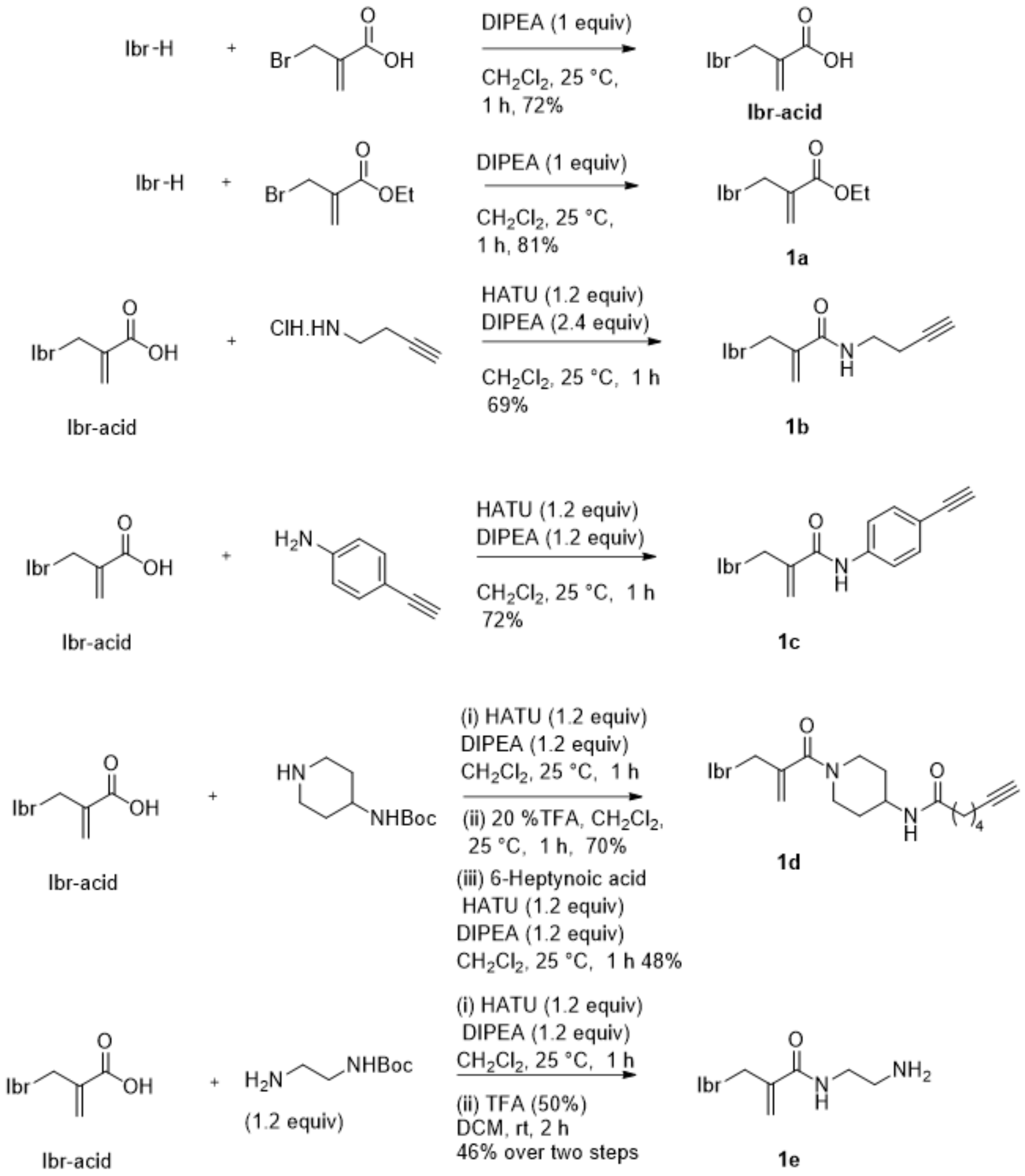
Ibr-H
Br
O
OH
DIPEA (1 equiv)
CH2Cl2, 25 °C,
1 h, 72%
Ibr
Ibr-acid
OEt
DIPEA (1 equiv)
CH2Cl2, 25 °C,
1 h, 81%
1a
ClH.HN
HATU (1.2 equiv)
DIPEA (2.4 equiv)
CH2Cl2, 25 °C, 1 h
69%
1b
H2N
HATU (1.2 equiv)
DIPEA (1.2 equiv)
CH2Cl2, 25 °C, 1 h
72%
1c
HN
NHBoc
(i) HATU (1.2 equiv)
DIPEA (1.2 equiv)
CH2Cl2, 25 °C, 1 h
(ii) 20 %TFA, CH2Cl2,
25 °C, 1 h, 70%
(iii) 6-Heptynoic acid
HATU (1.2 equiv)
DIPEA (1.2 equiv)
CH2Cl2, 25 °C, 1 h 48%
1d
H2N
NHBoc
(1.2 equiv)
(i) HATU (1.2 equiv)
DIPEA (1.2 equiv)
CH2Cl2, 25 °C, 1 h
(ii) TFA (50%)
DCM, rt, 2 h
46% over two steps
NH2
1e

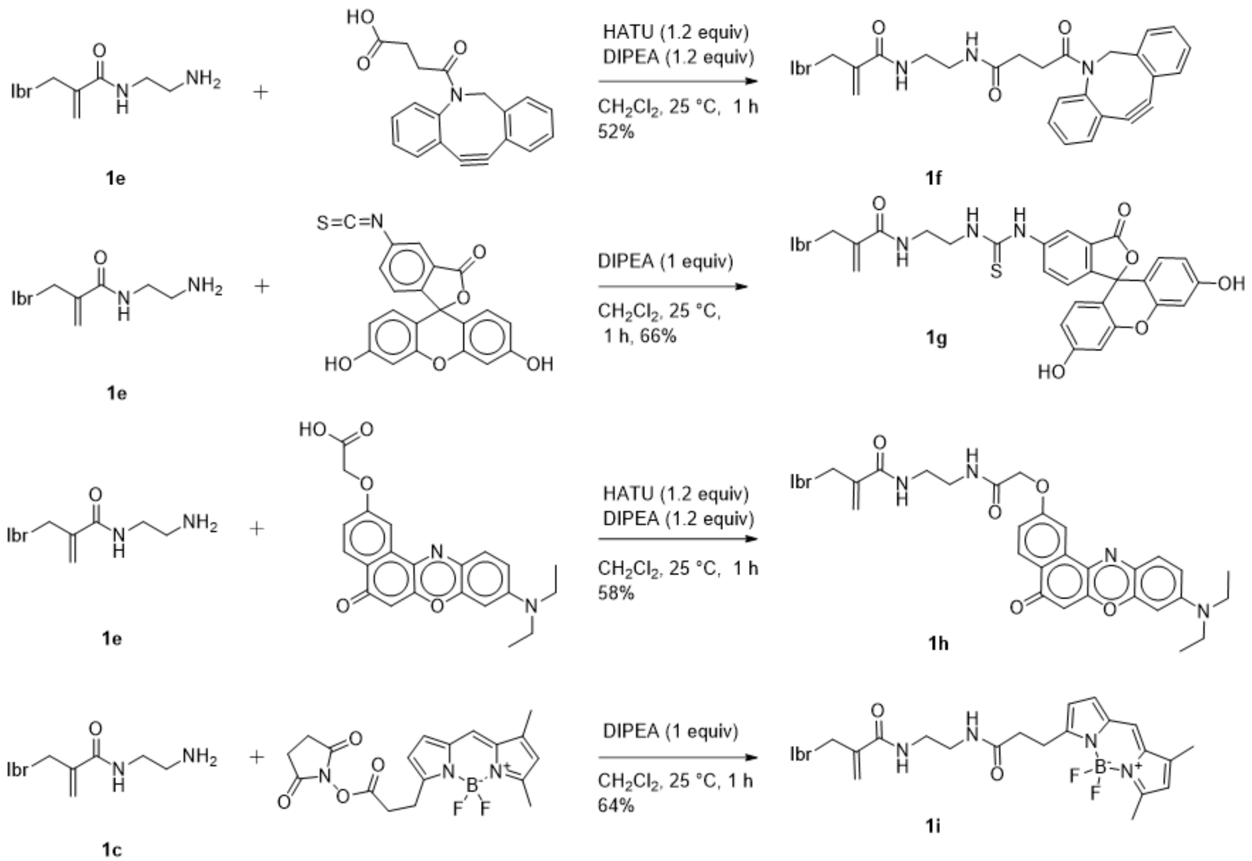
HATU (1.2 equiv)
DIPEA (1.2 equiv)
CH2Cl2, 25 °C, 1 h
52%
1e
1f
DIPEA (1 equiv)
CH2Cl2, 25 °C,
1 h, 66%
1e
1g
HATU (1.2 equiv)
DIPEA (1.2 equiv)
CH2Cl2, 25 °C, 1 h
58%
1e
1h
DIPEA (1 equiv)
CH2Cl2, 25 °C, 1 h
64%
1c
1i

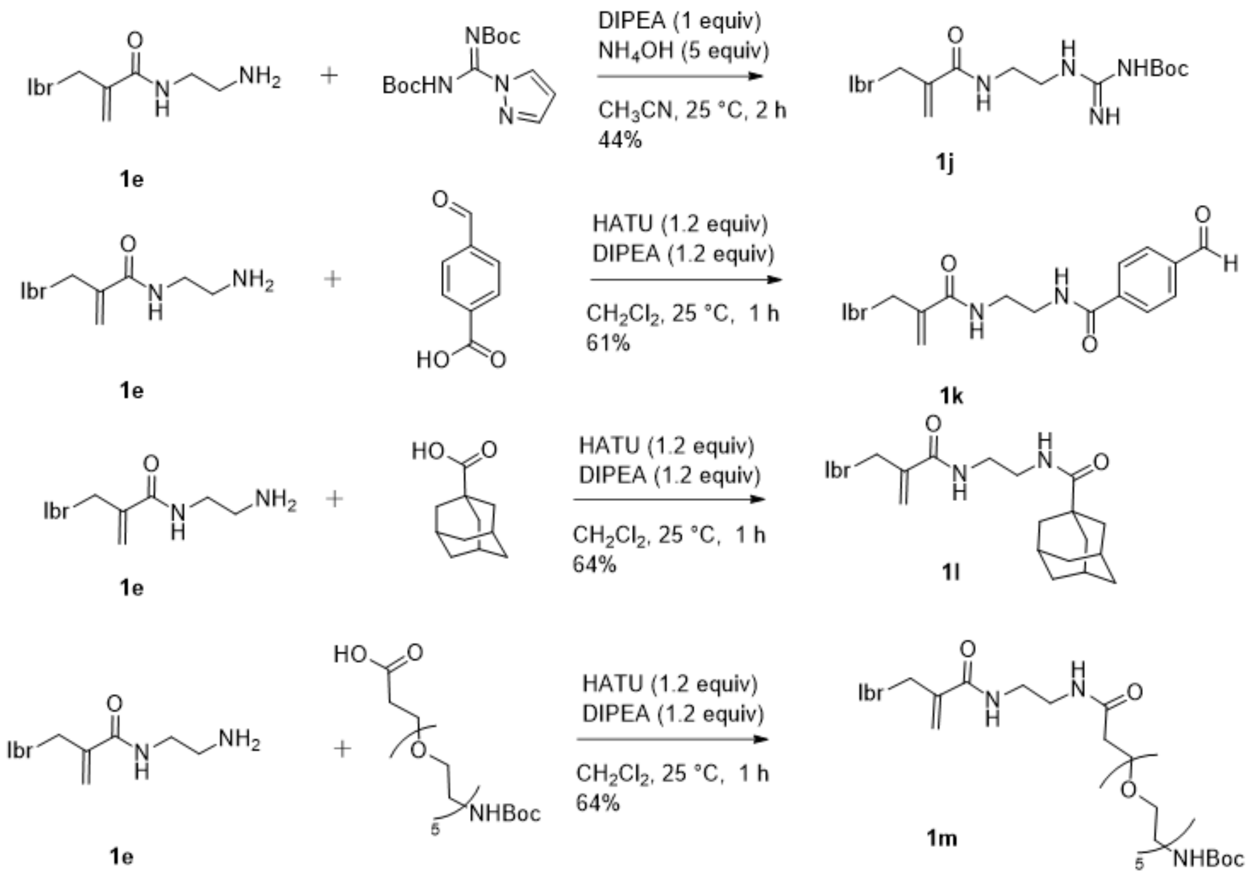


**Figure S2. Synthesis routes for Ibrutinib based BTK labelling probes.**

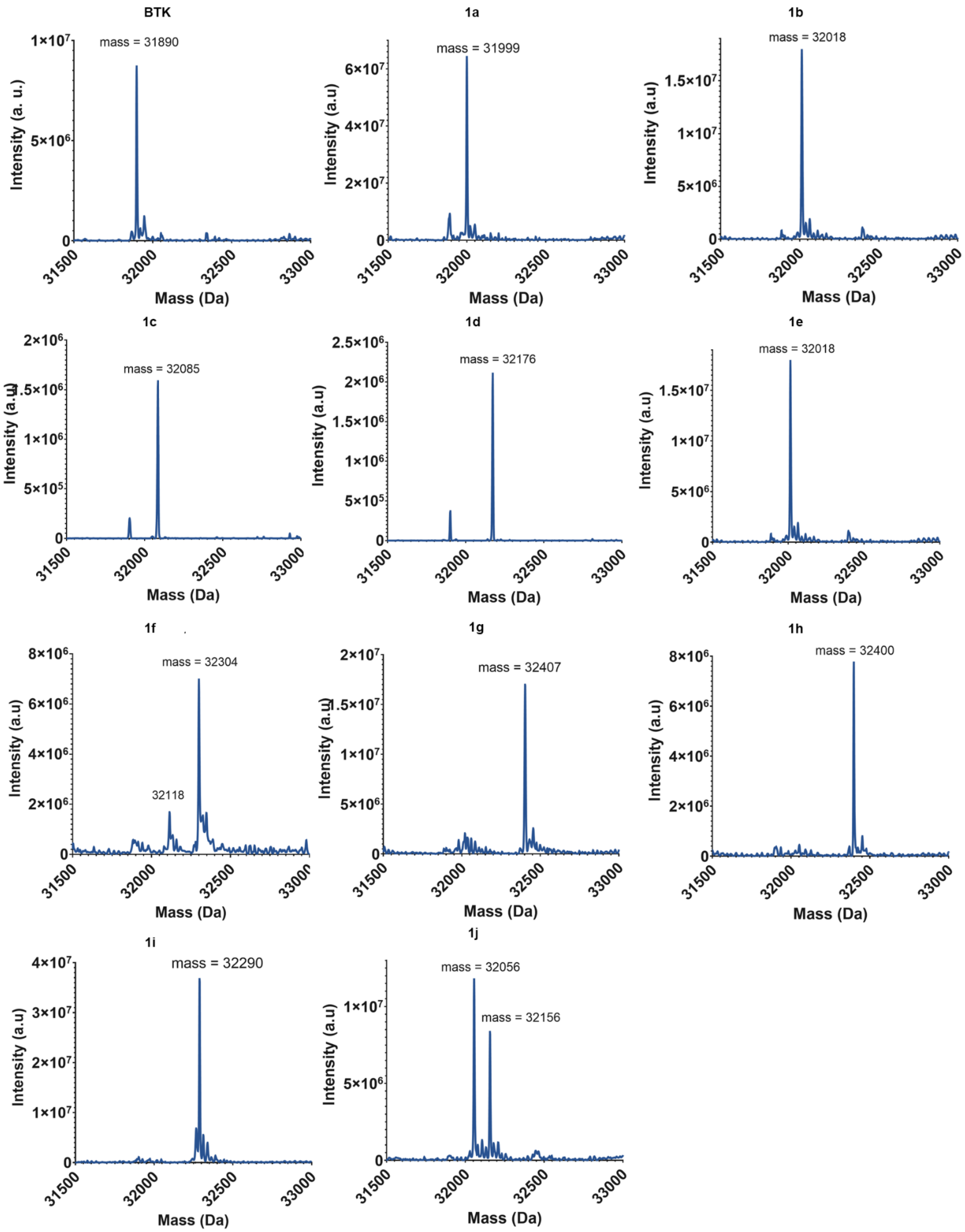

BTK
mass = 31890
1a
mass = 31999
1b
mass = 32018
1c
mass = 32085
1d
mass = 32176
1e
mass = 32018
1f
mass = 32304
32118
1g
mass = 32407
1h
mass = 32400
1i
mass = 32290
1j
mass = 32056
mass = 32156
Intensity (a. u.)
Intensity (a.u)
Mass (Da)
31500
32000
32500
33000

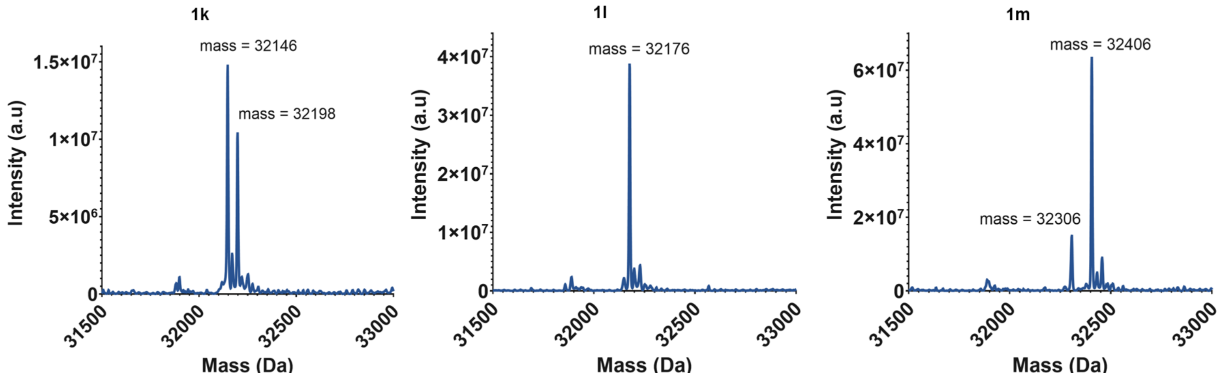


**Figure S3. Intact protein mass spectrometry validation of covalent adducts.** Deconvoluted mass spectra (intact protein LC/MS) of 2 µM BTK incubated with 2 µM Ibr-H derived probes **1a**-**1m** in 20 mM Tris buffer at pH 8.0, 25 $^{o}$C. Note that for **1j** and **1m**, an additional peak was observed with -100 Da due to removal of a Boc group, likely during the liquid chromatography, whereas for **1k** an $CD_3OD$ adduct was identified which may have bound during NMR analysis. Compound **1f** has a 15% hydrolysis product (obtained during HPLC) at the DBCO amide, which is also labeled BTK.

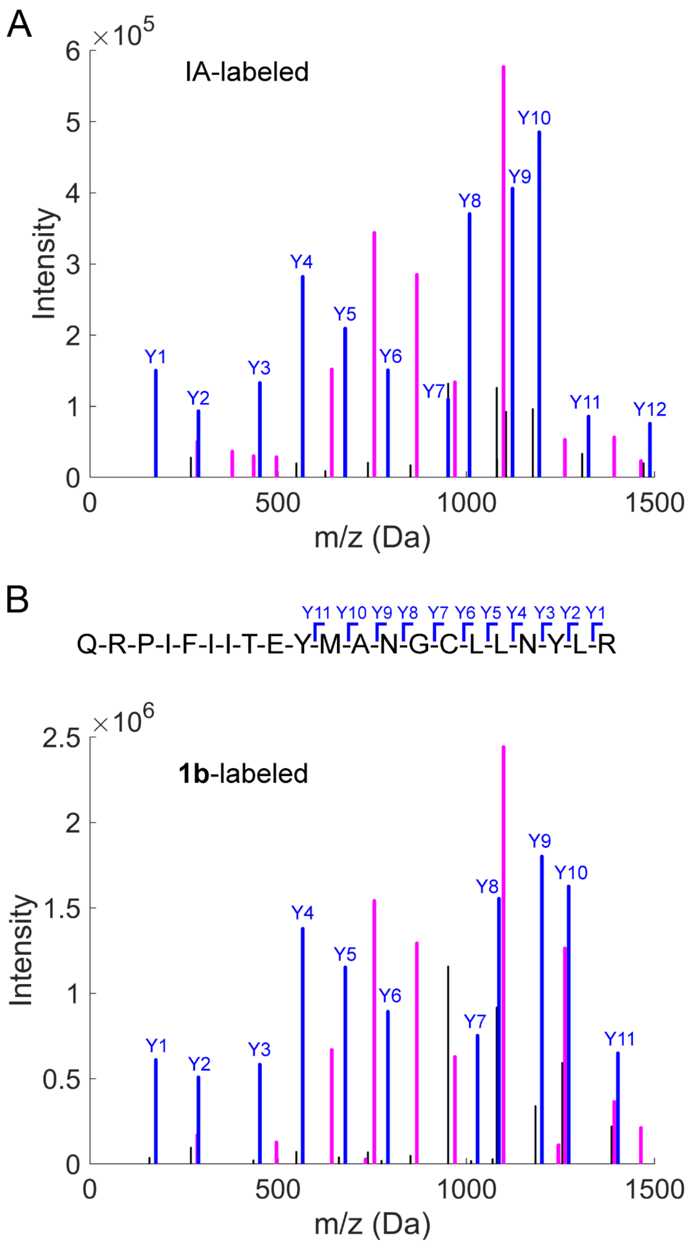


**Figure S4. Site specific labeling at BTK Cys481.** MS/MS spectra of the BTK (467-487) tryptic peptide. Following incubation with DMSO or **1b**, BTK was reduced, labeled with iodoacetamide (IA), digested with trypsin and analysed by LC-MSMS. **A.** MS/MS spectrum of the +3 charged precursor ion of the carbamidomethyl-modified 467-487 peptide, obtained after incubation of BTK with DMSO. **B.** MS/MS spectrum of the +3 charged precursor ion of the **1b**-modified 467-487 peptide, resulting from incubation of BTK with **1b**. Y ions are in blue, B ions in magenta, and other ions are in black. The gap between Y6 and Y7 corresponds to Cys481, and is noticeably larger for the **1b** modified peptide.

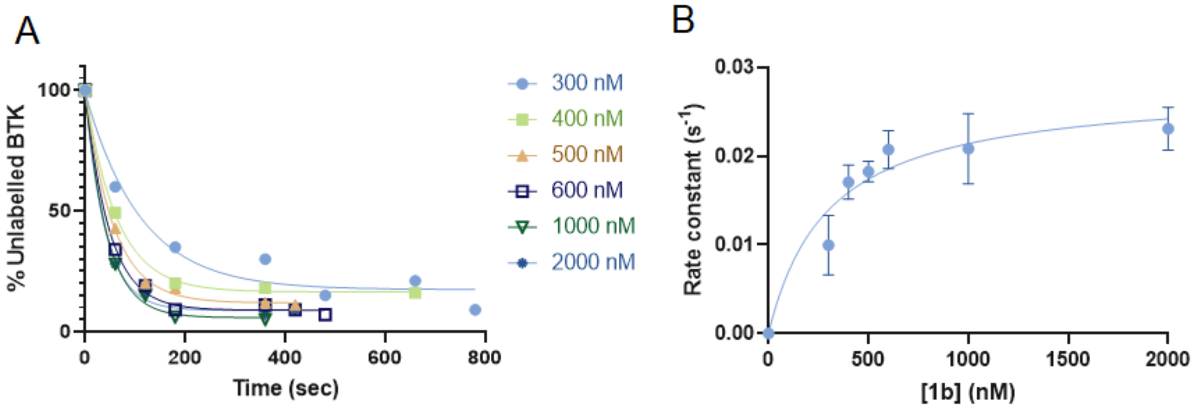


**Figure S5. Determination of $k_{inact}$ and $K_i$ parameters of CoLDR probe 1b. A**. Depletion of unlabelled BTK (200 nM) when incubated with **1b** (300, 400, 500, 600, 1000, 2000 nM) in 20 mM Tris buffer at pH 8, 14 °C over time. The reaction was monitored by LC-MS. **B.** The rate constants as extrapolated from A. as a function of the concentration of **1b**. Kinetic parameters were obtained by fitting this data to a Michaelis-Menten saturation curve. $k_{inact} = 2.78 \times 10^{-2}$ $s^{-1}$ and $K_i = 3.0 \times 10^{-7}$M. Error bars represent standard deviation. n = 2 (300, 1000, 2000 nM), n = 3 (400, 500, 600 nM).

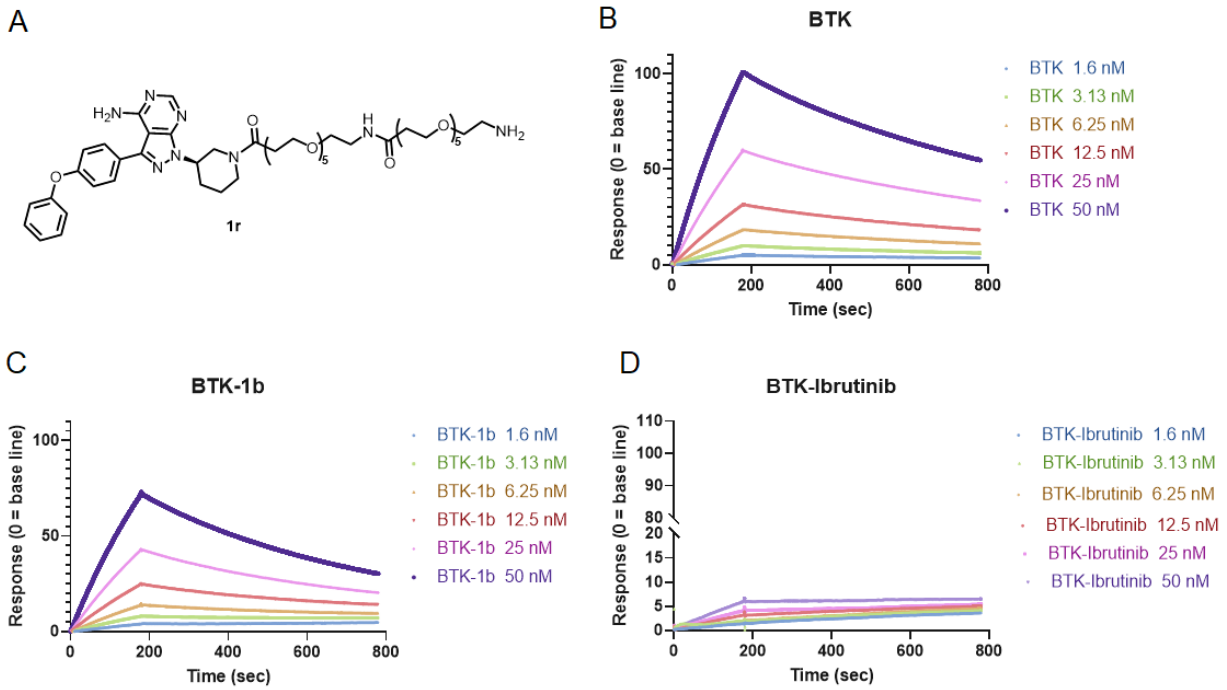


E

| | $k_a$ (1/Ms) | SE($k_a$) | $k_d$ (1/s) | SE($k_d$) | $K_D$ (M) | Chi² (RU²) |
|---|---|---|---|---|---|---|
| **BTK** | 9.04E+04 | 27 | 0.001361 | 2.60E-07 | 1.51E-08 | 0.0612 |
| **BTK-1b** | 1.76E+05 | 4.70E+02 | 0.003287 | 2.20E-06 | 1.87E-08 | 0.0568 |

**Figure S6. Labelling by CoLDR probes does not affect ligand binding. A.** Structure of the Ibrutinib based reversible compound used to label the SPR chip. **B-D.** Surface plasmon resonance (SPR) sensorgrams for (B) BTK, (C) BTK-**1b** and (D) BTK-ibrutinib at different concentrations. **E.** Kinetic parameters for association ($k_a$) and dissociation constants ($k_d$) for BTK and BTK-**1b.** SE = Standard Error.

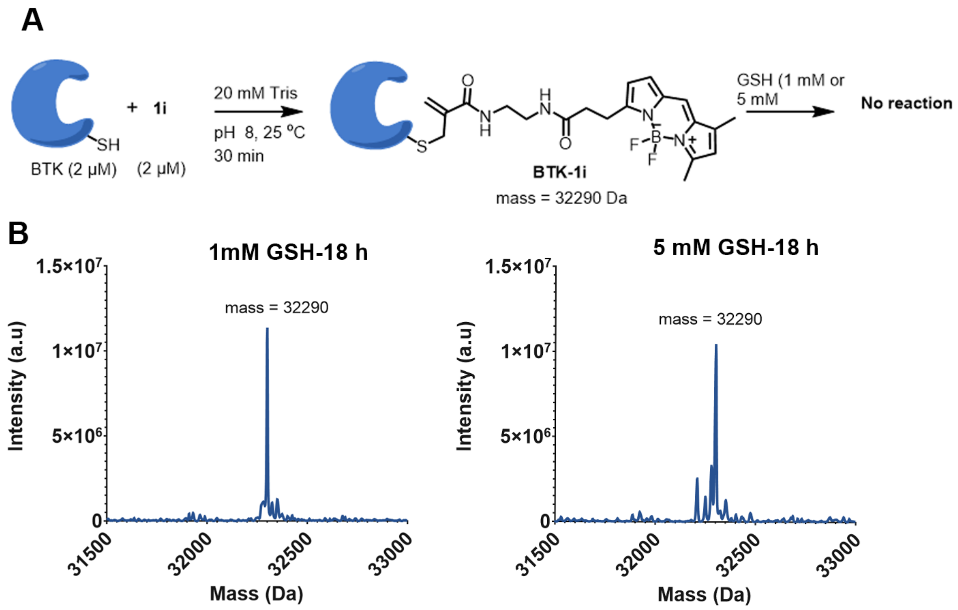


**Figure S7**. **Stability of BTK labelled by 1i**. **A**. BTK (2 μM) was incubated with **1i** (2 μM) for 30 min at pH 8, 25 °C. The labelled BTK was treated with GSH (1 mM or 5 mM) for 18 h at 25 °C. **B**. Deconvoluted mass spectra (intact protein LC/MS) of BTK-**1i** treated with GSH 1 mM and 5 mM.

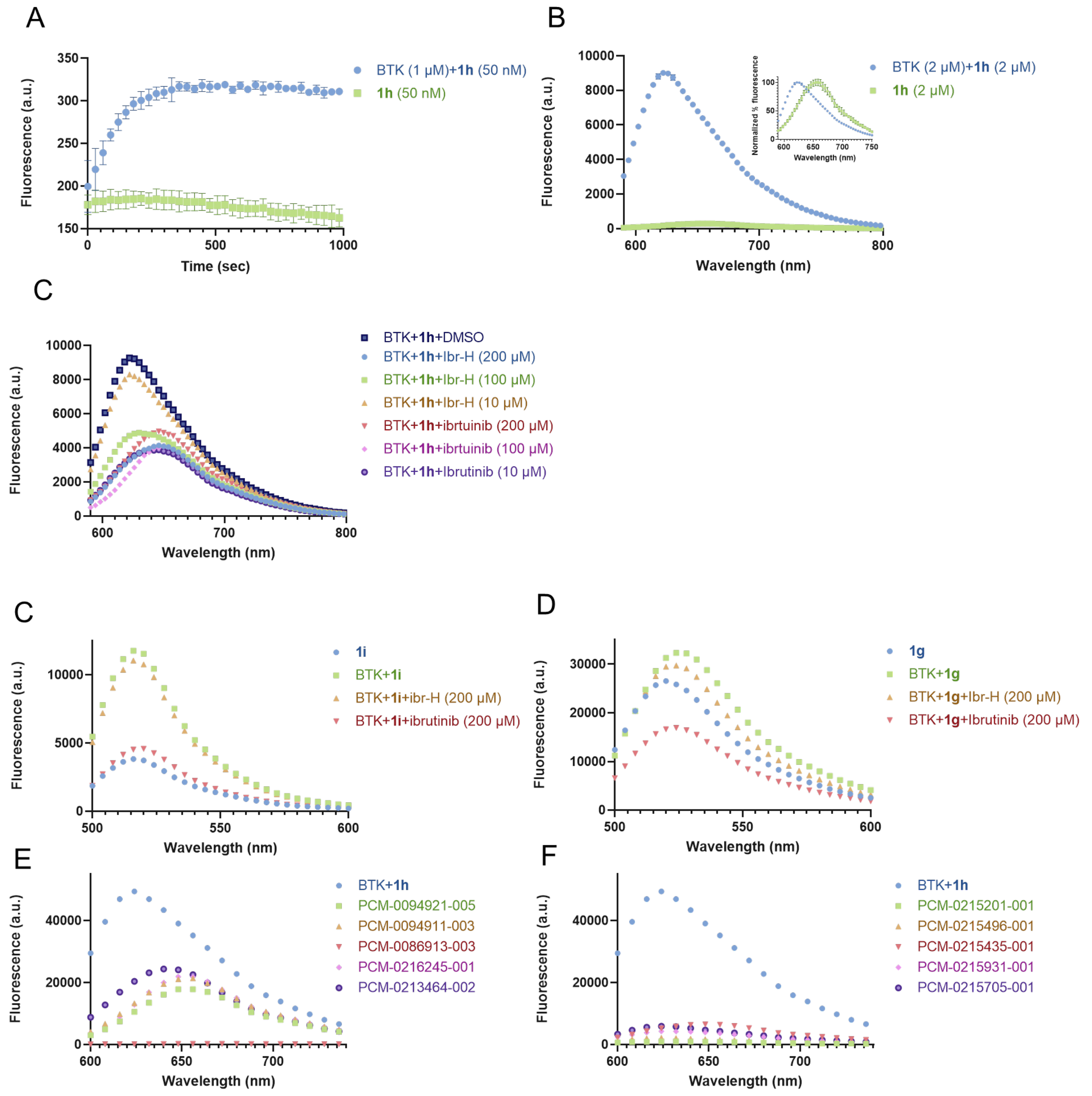


**Figure S8. Turn-on fluorescent environmental sensitive probe can detect binding events to BTK. A.** Incubation of **1h** with BTK at low equivalents (1 µM BTK; 50 nM **1h**; Ex/Em = 550/620 nm) still shows a detectable increase in fluorescence, but considerably slows down the reaction, to a point that the initial kinetics can be observed (n = 2). **B.** Fluorescence spectrum scan of **1h** (2 µM) in the presence/absence of BTK (2 µM). Inset shows the normalized fluorescence spectrum, where it is evident there is a shift in the peak upon protein binding. **C.** Dose dependent reduction of the fluorescence, and shift of the peak emission of BTK labelled **1h** after the addition of excess ligands (Ibrutinib and Ibr-H). **D.** Three- fold increase in the fluorescence intensity of **1i** (2 µM) when incubated with BTK (2 µM) and reduction of the fluorescence after the addition of excess ligand. **E.** Changes in the fluorescence intensity of **1g** (2 µM) after the addition of BTK (2 µM) followed by Ibrutinib and Ibr-H. **F.** Fluorescence scan of BTK labelled **1h** (2 µM) incubated with various BTK binders shows more than 2.5 fold change in the 650/620 emission ratio. **G.** BTK inhibitors caused significant quenching of fluorescence of BTK-**1h.**

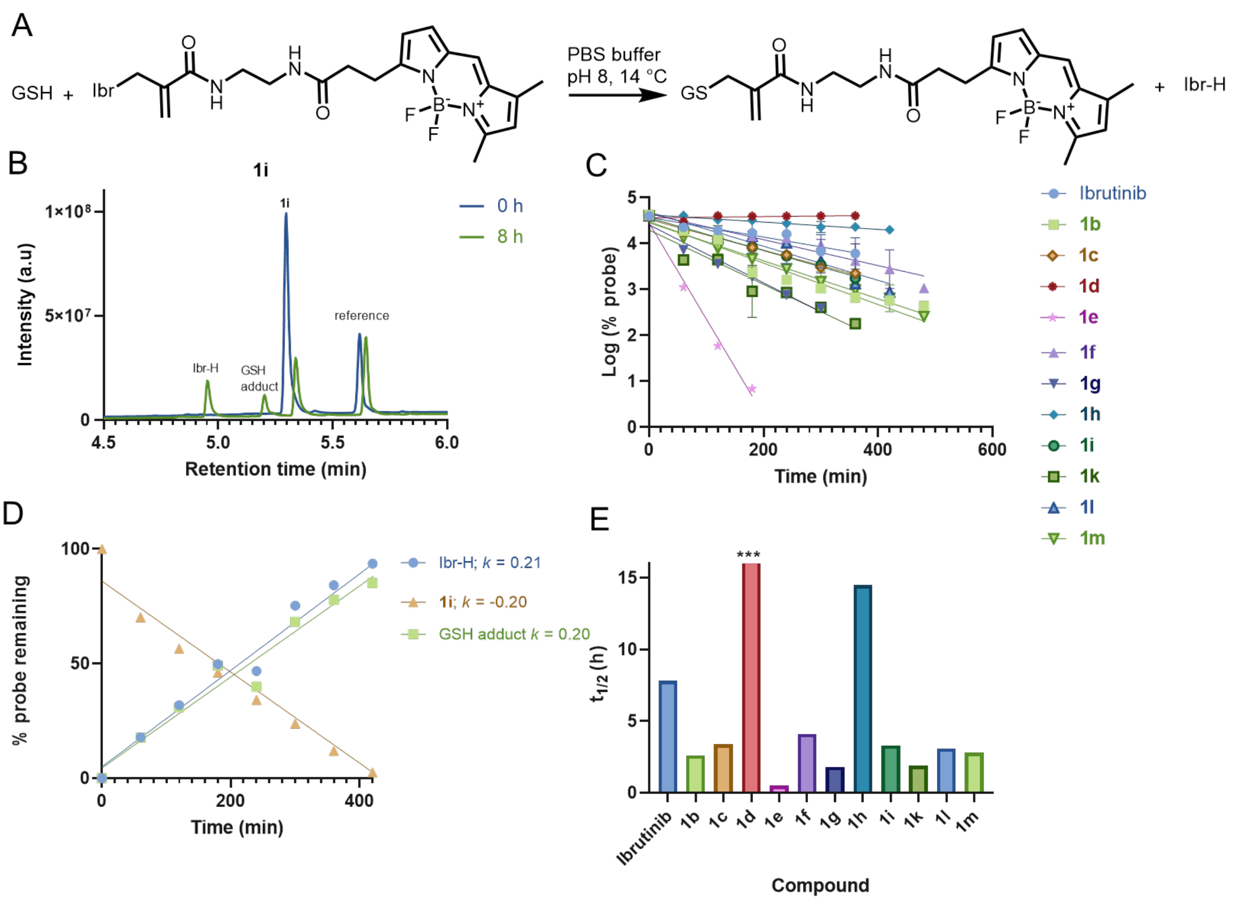


**Figure S9. Reaction with reduced GSH validates the elimination of ligands and demonstrates their intrinsic thiol reactivity is tunable. A.** A typical example of the reaction of GSH with **1i** in 100 mM PBS buffer at pH 8, 14 °C. **B**. An example LC chromatogram shows monitoring of the reaction of **1i** (100 µM) with GSH (5 mM) at 0 h (blue) and 8 h (green) GSH adduct: Retention time (RT) = 5.17 min, m/z = 707; Ibr-H: RT = 5.0 min; reference: RT = 5.60 min; **1i**; RT = 5.38 min; m/z = 786. UV absorption measured between 220-400 nm. **C.** Rates of depletion of Ibr-H derivatives (**1a**-**1m**) in a reaction between 100 µM compound and 5 mM GSH in PBS buffer at pH 8, 14 °C (n=2) for 8 h. **D.** Rates of formation in LC-MS (absorption 220-400 nm) of Ibr-H, GSH adduct and depletion of **1i** in a reaction between 100 µM **1i** and 5 mM GSH in PBS buffer, pH 8, 37 °C (n=2). **E**. GSH $t_{1/2}$ of all the probes and Ibrutinib. *** $t_{1/2}$ >100 h. We should note that compound **1a**, reacted completely within 10 min and is not shown.

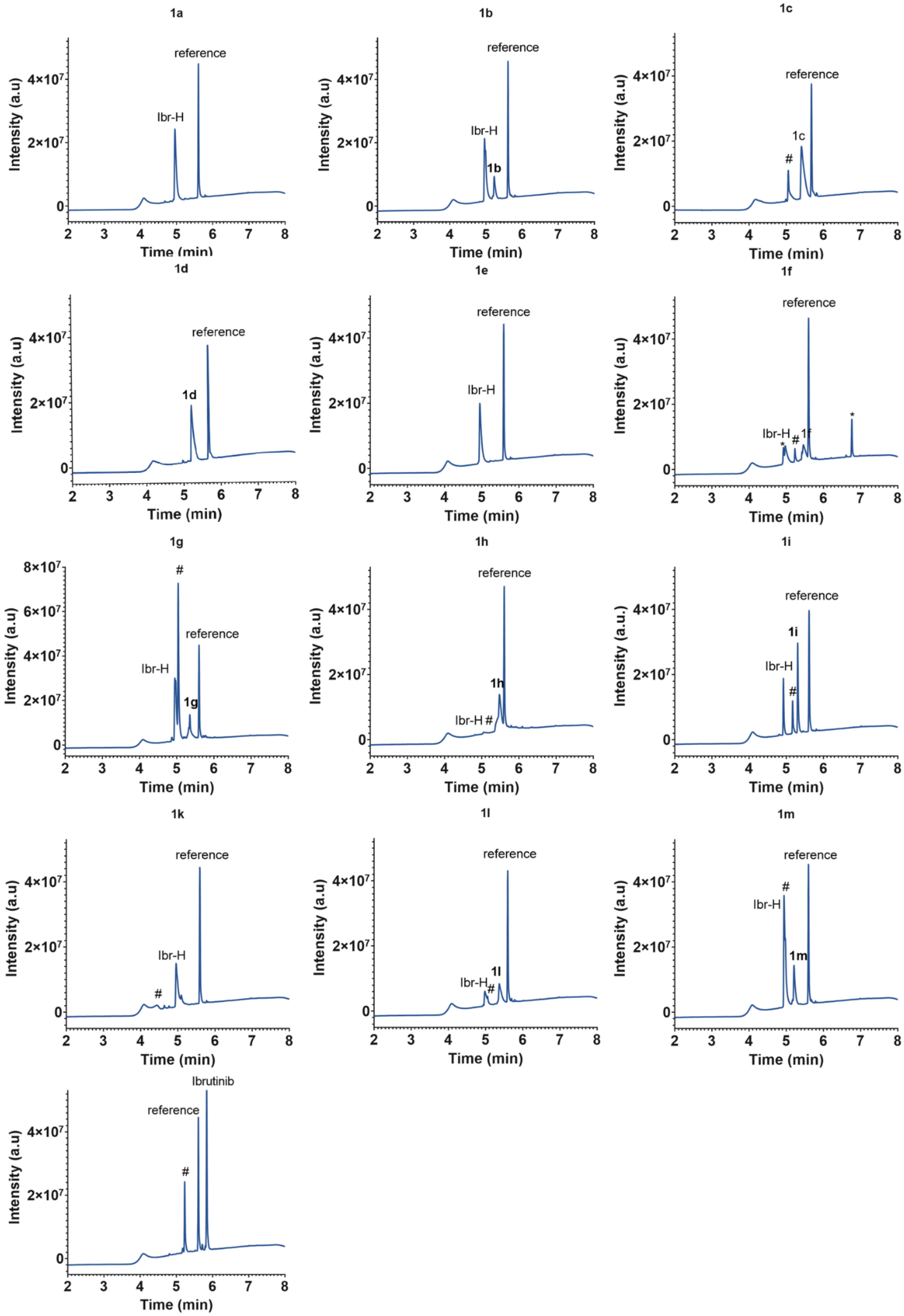
1a
reference
Ibr-H
Intensity (a.u)
Time (min)
1b
reference
Ibr-H
1b
Intensity (a.u)
Time (min)
1c
reference
1c
#
Intensity (a.u)
Time (min)
1d
reference
1d
Intensity (a.u)
Time (min)
1e
reference
Ibr-H
Intensity (a.u)
Time (min)
1f
reference
Ibr-H
*
#
1f
*
Intensity (a.u)
Time (min)
1g
#
reference
Ibr-H
1g
Intensity (a.u)
Time (min)
1h
reference
1h
Ibr-H
#
Intensity (a.u)
Time (min)
1i
reference
1i
Ibr-H
#
Intensity (a.u.)
Time (min)
1k
reference
Ibr-H
#
Intensity (a.u)
Time (min)
1l
reference
1l
Ibr-H
#
Intensity (a.u)
Time (min)
1m
reference
#
Ibr-H
1m
Intensity (a.u)
Time (min)
Ibrutinib
reference
#
Intensity (a.u)
Time (min)

**Figure S10. GSH reactivity assay with Ibrutinib analogues.** UV spectra (220-400 nm) of the LC/MS analysis of 5 mM GSH incubated with 100 µM of compounds (**1a**-**1m**) at the last measured point of the GSH $t_{1/2}$ experiment (Figure S9). The small peak at 4 min is contamination stuck on the column. * The hydrolyzed **1f** impurity before the GSH assay. # GSH adduct.

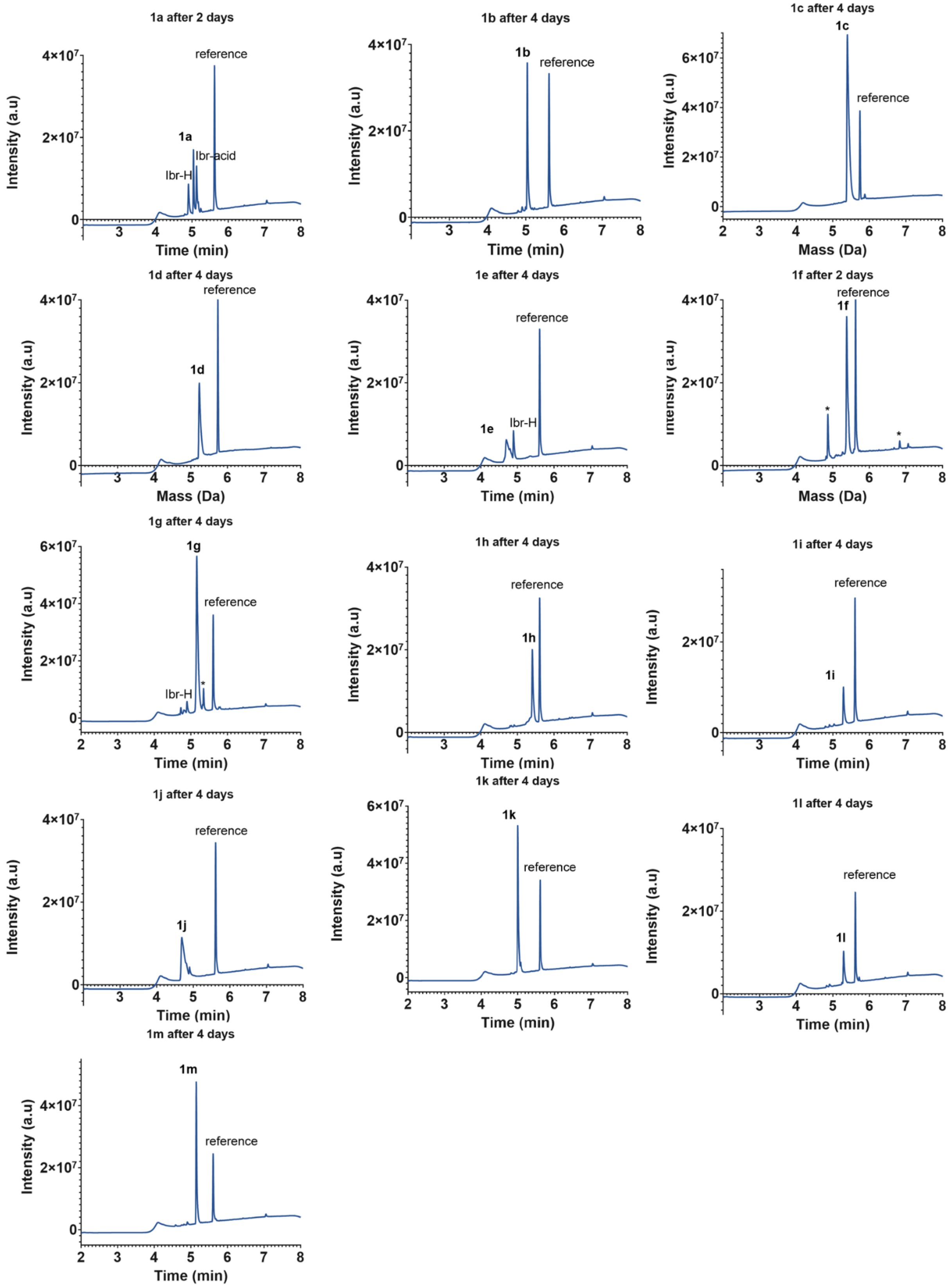
1a after 2 days
reference
1a
Ibr-acid
Ibr-H
Intensity (a.u)
Time (min)
1b after 4 days
1b
reference
Intensity (a.u)
Time (min)
1c after 4 days
1c
reference
Intensity (a.u)
Mass (Da)
1d after 4 days
reference
1d
Intensity (a.u)
Mass (Da)
1e after 4 days
reference
1e
Ibr-H
Intensity (a.u)
Time (min)
1f after 2 days
reference
1f
*
*
Intensity (a.u)
Mass (Da)
1g after 4 days
1g
reference
Ibr-H
*
Intensity (a.u)
Time (min)
1h after 4 days
reference
1h
Intensity (a.u)
Time (min)
1i after 4 days
reference
1i
Intensity (a.u)
Time (min)
1j after 4 days
reference
1j
Intensity (a.u)
Time (min)
1k after 4 days
1k
reference
Intensity (a.u)
Time (min)
1l after 4 days
reference
1l
Intensity (a.u)
Time (min)
1m after 4 days
1m
reference
Intensity (a.u)
Time (min)

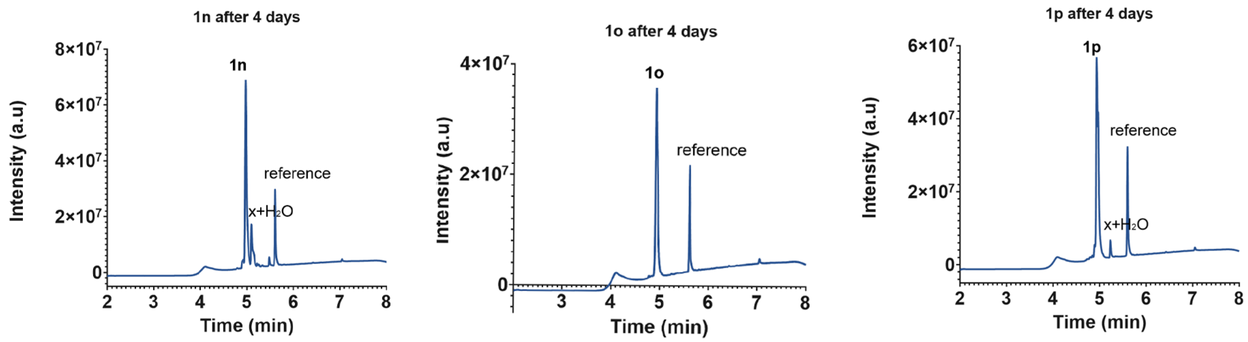


**Figure S11. Ibrutinib analogs show prolonged buffer stability.** UV spectra (220-400 nm) of the LC/MS analysis of 100 µM of compounds (**1a**-**1p**) incubated with reference (100 µM) in the PBS buffer at pH 8, 37 °C. The small peak at 4 min is contamination stuck to the column. **1e** and **1g** underwent 25% and 5% Ibr-H elimination respectively after 4 days whereas **1a** underwent 100% hydrolysis/elimination in two days. For compounds **1n** (15%) and **1p** (5%), a water adduct was formed, probably on thalidomide/lenalidomide. *Compound **1f** has a 15% hydrolysis product (obtained during HPLC) at the DBCO amide. No further hydrolysis has been observed under these buffer conditions.

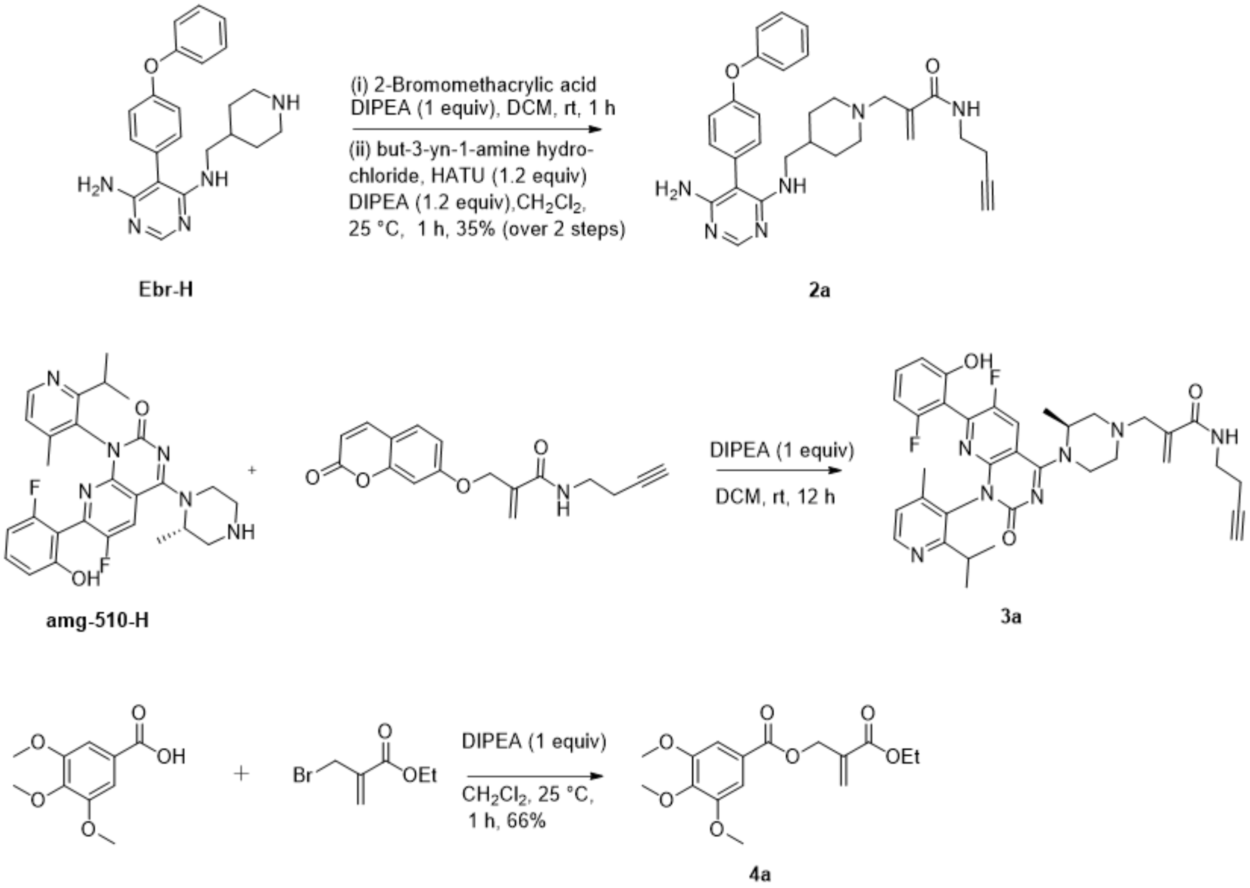


**Figure S12.** Synthesis routes for **2a**-**4a**.

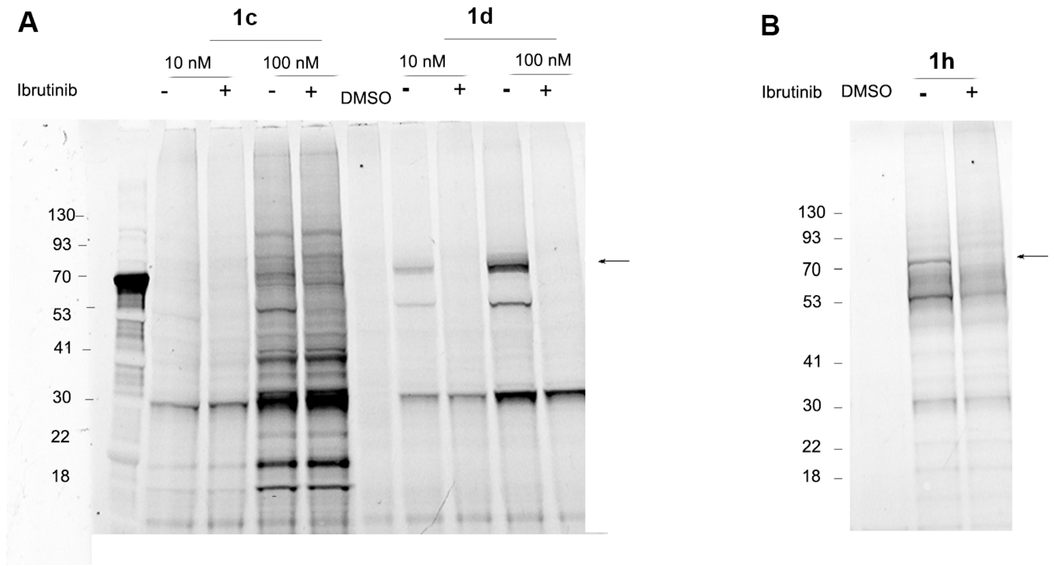


**Figure S13. Selectivity can be improved by modulating the CoLDR substituent A.** Cellular Labeling profile and competition with Ibrutinib for **1c**, **1d** and **B. 1h**. The cells were pre-incubated for 30 min with either 0.1% DMSO or 1 µM Ibrutinib, followed by 2 h incubation with 10 and 100 nM **1c** or **1d** or 100 nM of **1h**. after 2 h incubation with Mino cells. **1c** and **1d** samples were further reacted with TAMRA-azide in lysate before imaging. An arrow indicates BTK's MW.

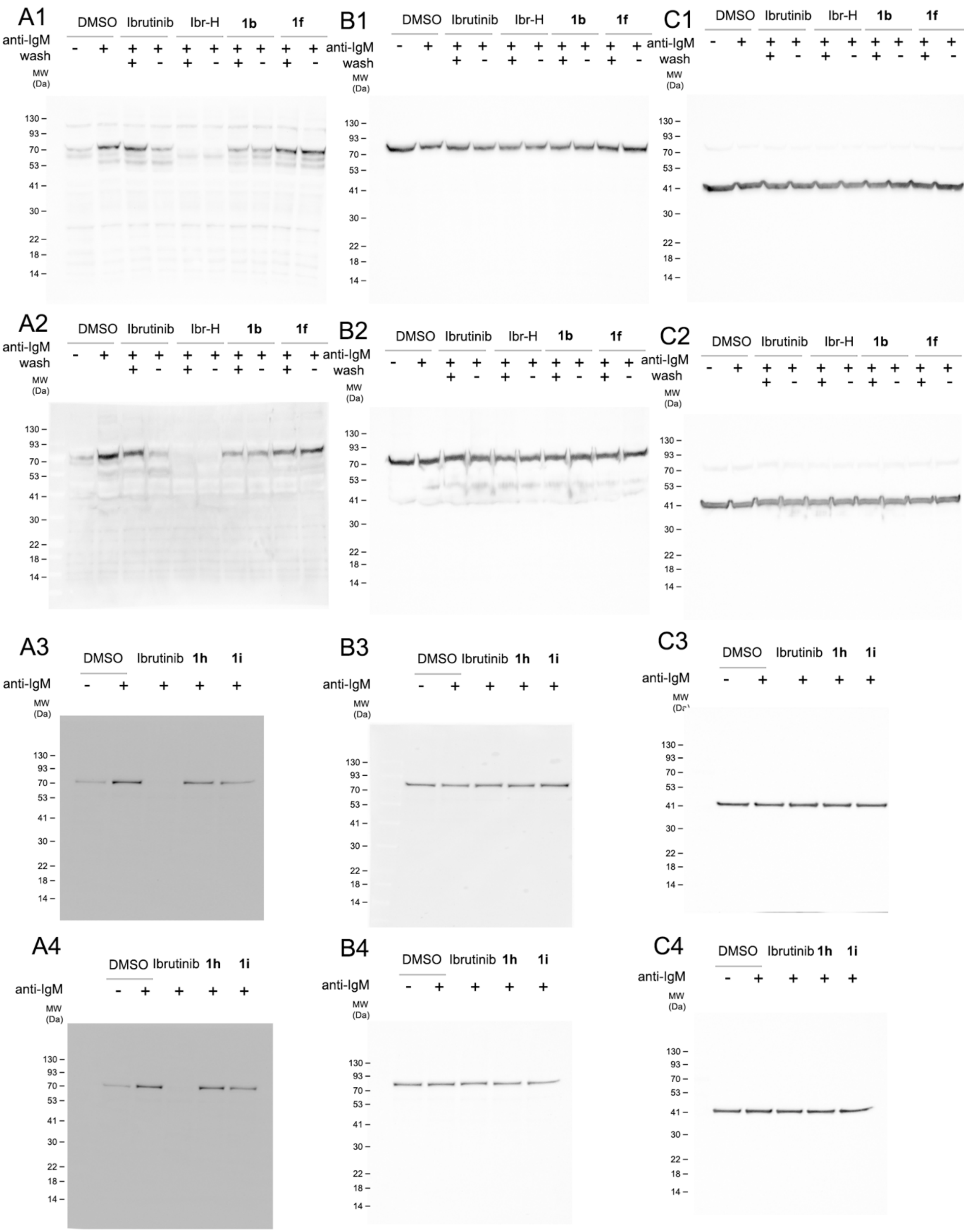
A1
DMSO Ibrutinib Ibr-H 1b 1f
anti-IgM
wash
MW (Da)
130 93 70 53 41 30 22 18 14
B1
DMSO Ibrutinib Ibr-H 1b 1f
anti-IgM
wash
MW (Da)
C1
DMSO Ibrutinib Ibr-H 1b 1f
anti-IgM
wash
MW (Da)
A2
DMSO Ibrutinib Ibr-H 1b 1f
anti-IgM
wash
MW (Da)
B2
DMSO Ibrutinib Ibr-H 1b 1f
anti-IgM
wash
MW (Da)
C2
DMSO Ibrutinib Ibr-H 1b 1f
anti-IgM
wash
MW (Da)
A3
DMSO Ibrutinib 1h 1i
anti-IgM
MW (Da)
B3
DMSO Ibrutinib 1h 1i
anti-IgM
MW (Da)
C3
DMSO Ibrutinib 1h 1i
anti-IgM
MW
A4
DMSO Ibrutinib 1h 1i
anti-IgM
MW (Da)
B4
DMSO Ibrutinib 1h 1i
anti-IgM
MW (Da)
C4
DMSO Ibrutinib 1h 1i
anti-IgM
MW (Da)

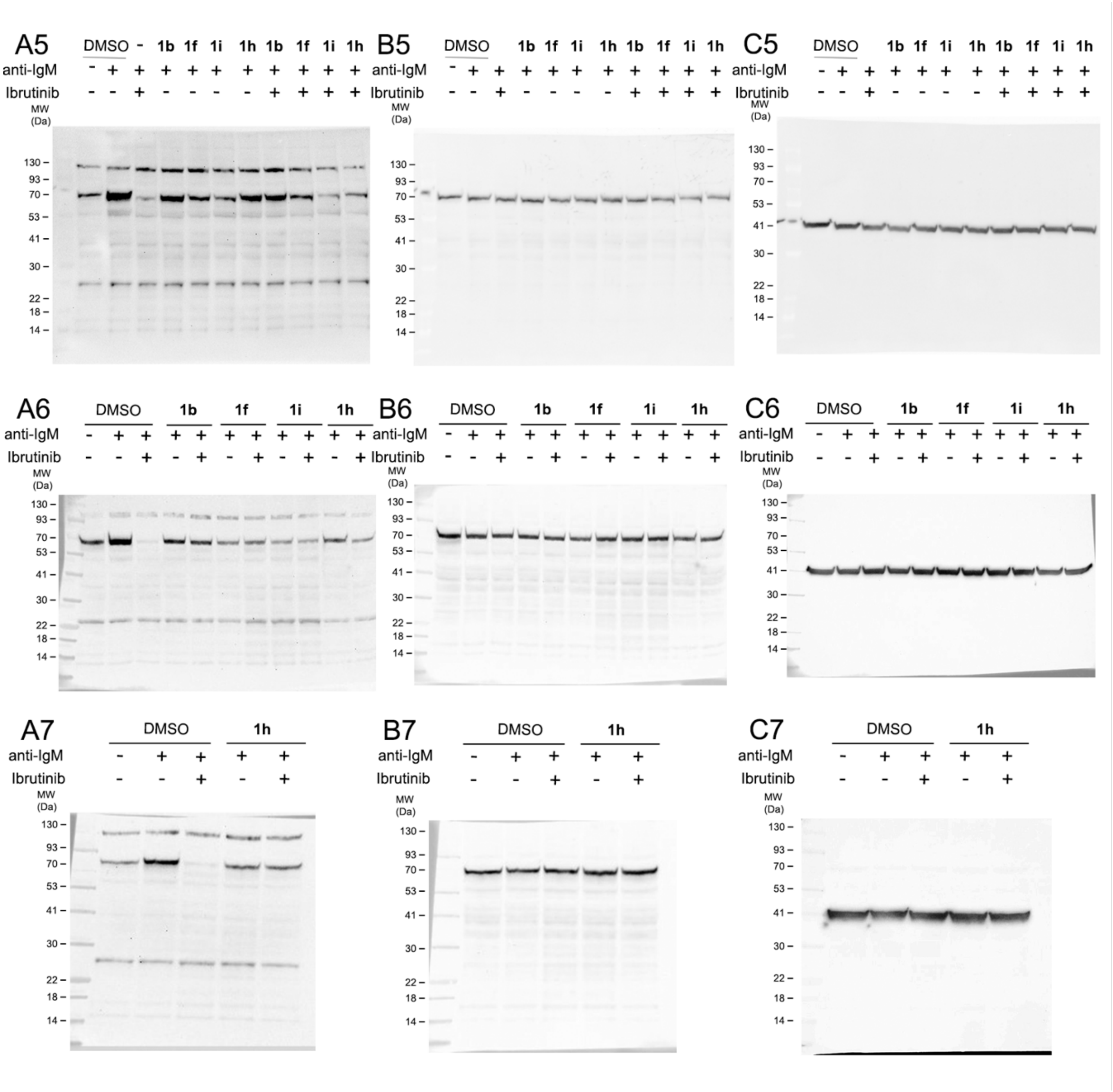


**Figure S14. Labeling BTK with CoLDR probes does not inhibit its activity in cells.** BTK activity assay in Mino cells as measured by BTK's autophosphorylation. The cells were incubated for 1 hour with either 0.1% DMSO, 1 µM Ibrutinib, 1 µM Ibr-H, or 100 nM **1b** and **1f**. The cells were either washed or not before induction of BTK's activity by anti-IgM. **A1** (phospho BTK), **B1** (total BTK) and **C1** (β-actin). **A2** (phospho BTK), **B2** (total BTK) and **C2** (β-actin) shows the western blots of a second repetition. **A3, A4** (phospho BTK), **B3, B4** (total BTK) and **C3**, **C4** (β –actin) shows western blots for BTK activity assay for **1h** and **1i** without wash. **A5, A6** (phospho BTK), **B5, B6** (total BTK), **C5, C6** (β-actin) shows the western blots for BTK activity assay: Mino cells were incubated for 2 h with either DMSO, 1 uM **1b**, **1f, 1i** and **1h**, washed and then incubated for 45 min with Ibrutinib (100 nM). The cells were washed again before induction of BTK activity by anti-IgM. **A7** (phospho BTK), **B7** (total BTK), **C7** (β-actin) shows western blots for BTK activity assay: Mino cells were incubated for 4 h with either DMSO or 5 uM **1h**, washed and then incubated for 45 min with Ibrutinib (100 nM). The cells were washed again before induction of BTK activity by anti-IgM.

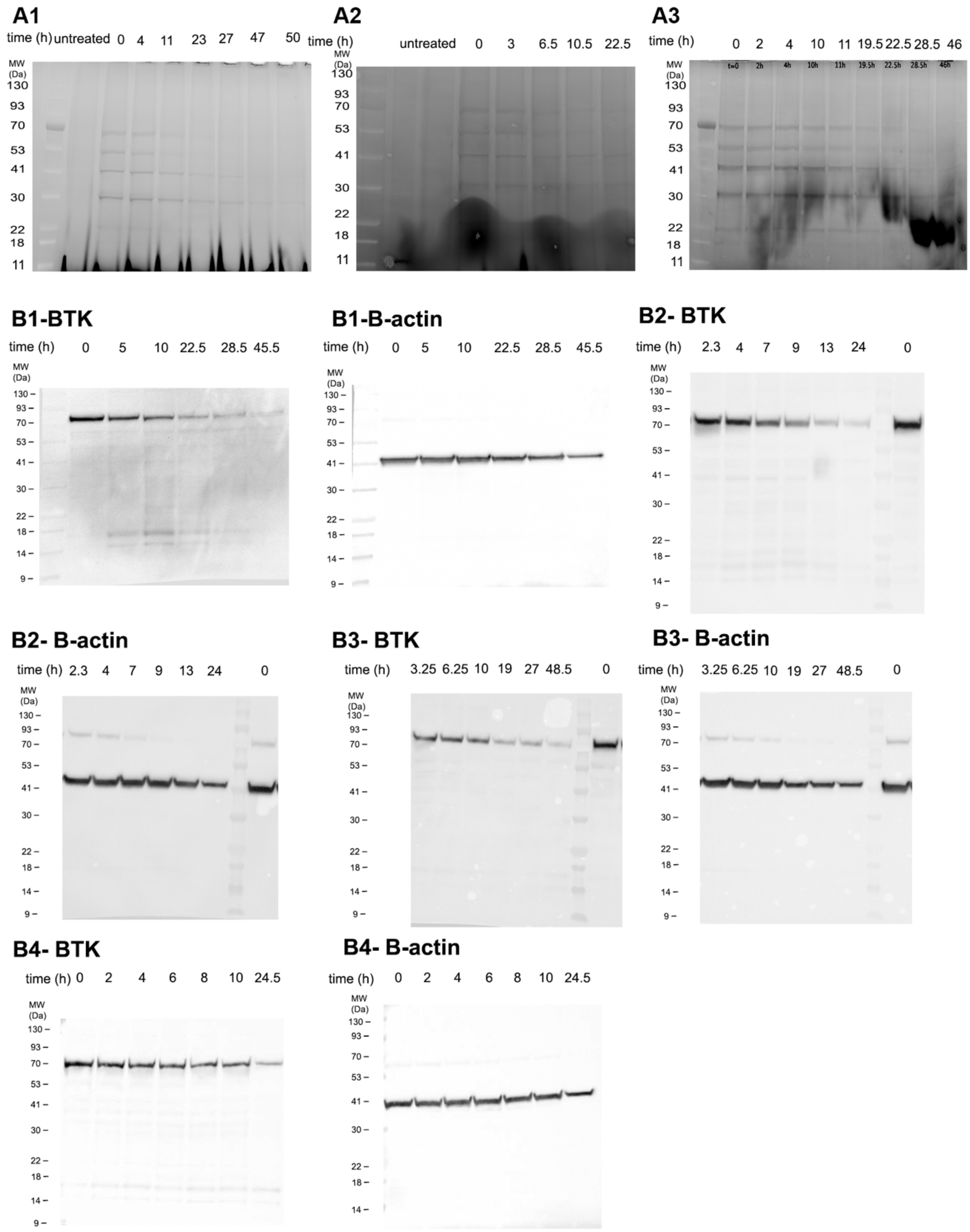


**Figure S15. Half-life measurements of BTK. A1, A2, A3** shows half-life measurement of BTK using **1f**. Mino cells were pulse-labeled with 100 nM **1f** for 1 hour and were then washed to remove excess compound. Cells were harvested at the indicated time-points and lysates were clicked with TAMRA-Azide. BTK's signal was quantified and the half-

life was calculated (n = 3). **B1, B2, B3, B4**. Half-life measurement of BTK with cycloheximide (CHX) assay, using 20 μg/ml cycloheximide (n=4).

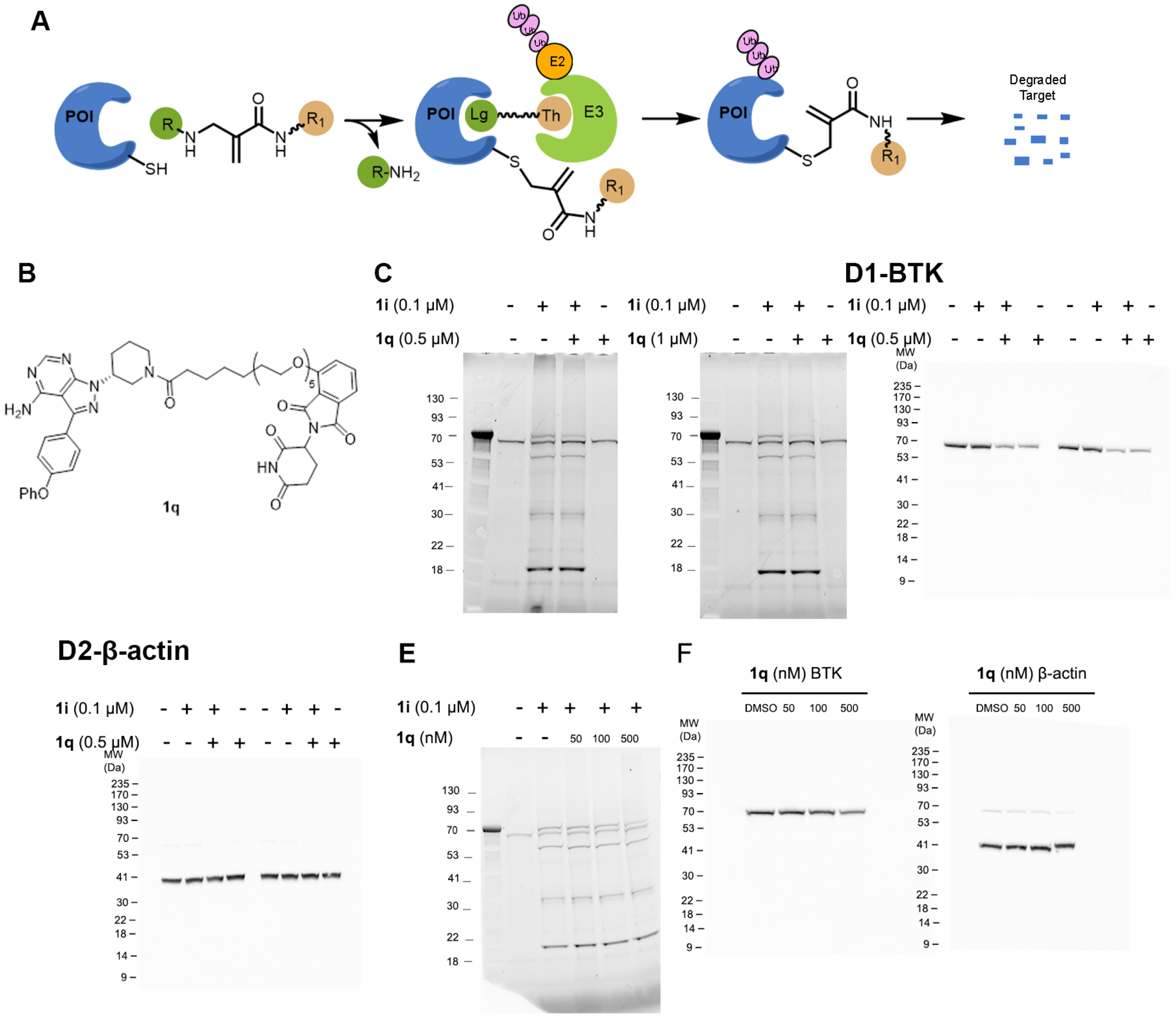


**Figure S16. Fluorescent Labelling does not inhibit active site binding and ternary complex formation. A.** Schematic representation of protein labelled with CoLDR probe followed degradation with PROTAC. **B.** Structure of reversible PROTAC **1q. C, D, E.** Mino cells were treated with **1i** for 1 h washed and incubated with **1q** at various concentrations. Degradation was measured using in-gel fluorescence (**C and E**) and Western blot (**D**). **F.** BTK degradation by **1q** at 50, 100, 500 nM measured using Western Blot.

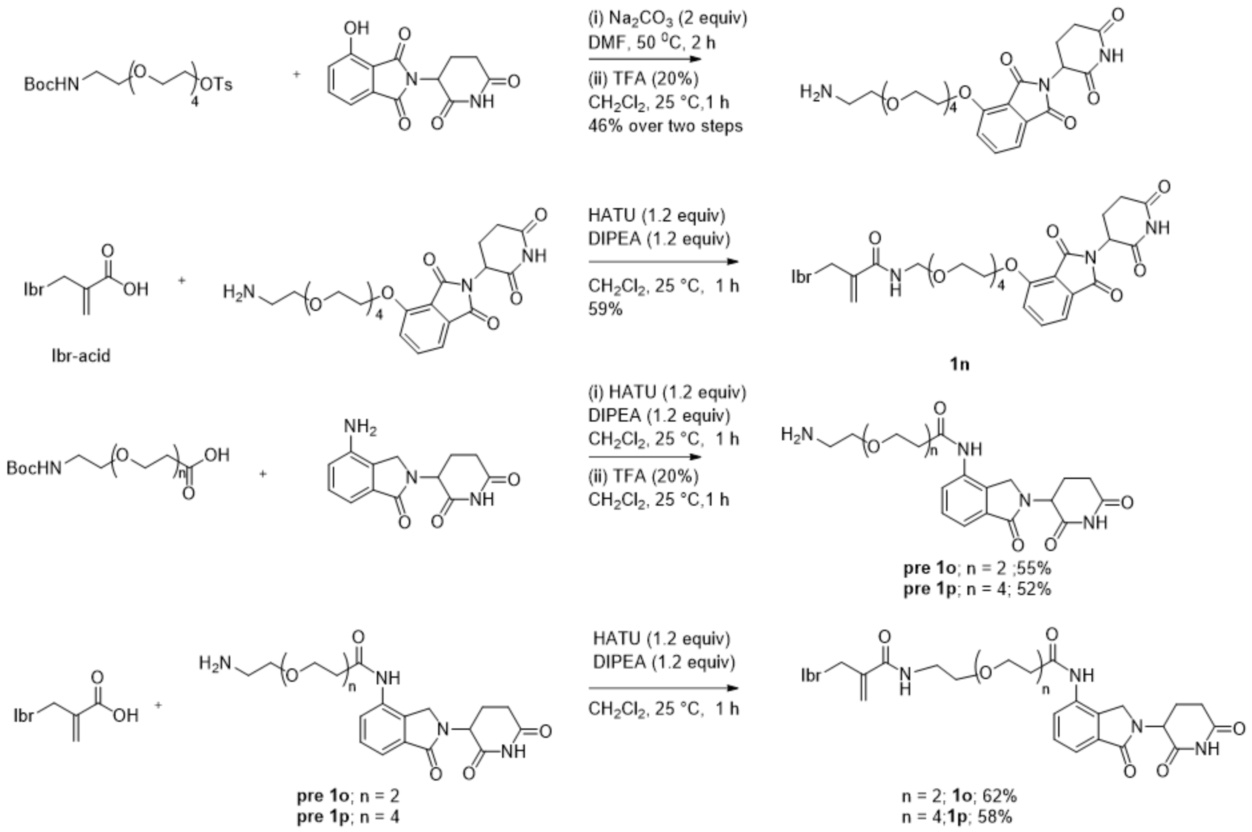


**Figure S17. Synthesis routes of CoLDR BTK PROTACs.**

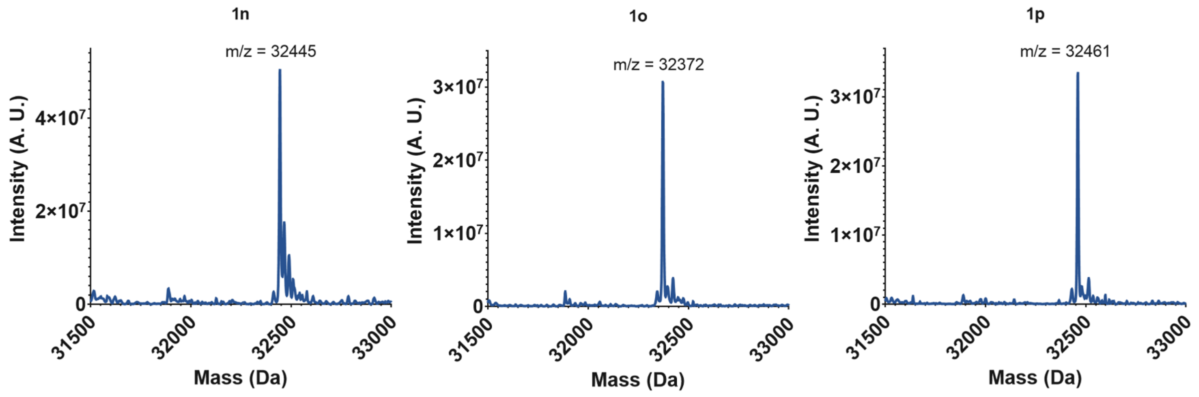


**Figure S18. CoLDR PROTACs label BTK with a degradation handle.** Deconvoluted MS spectra (intact protein LC/MS) of 2 µM BTK incubated with 2 µM ibr-H derived PROTACs **1n**-**1p** in 20 mM Tris buffer at pH 8.0, 25 $^o$C. The spectra were collected after 30 min incubation for **1n** and after 2 h for **1o** and **1p.**

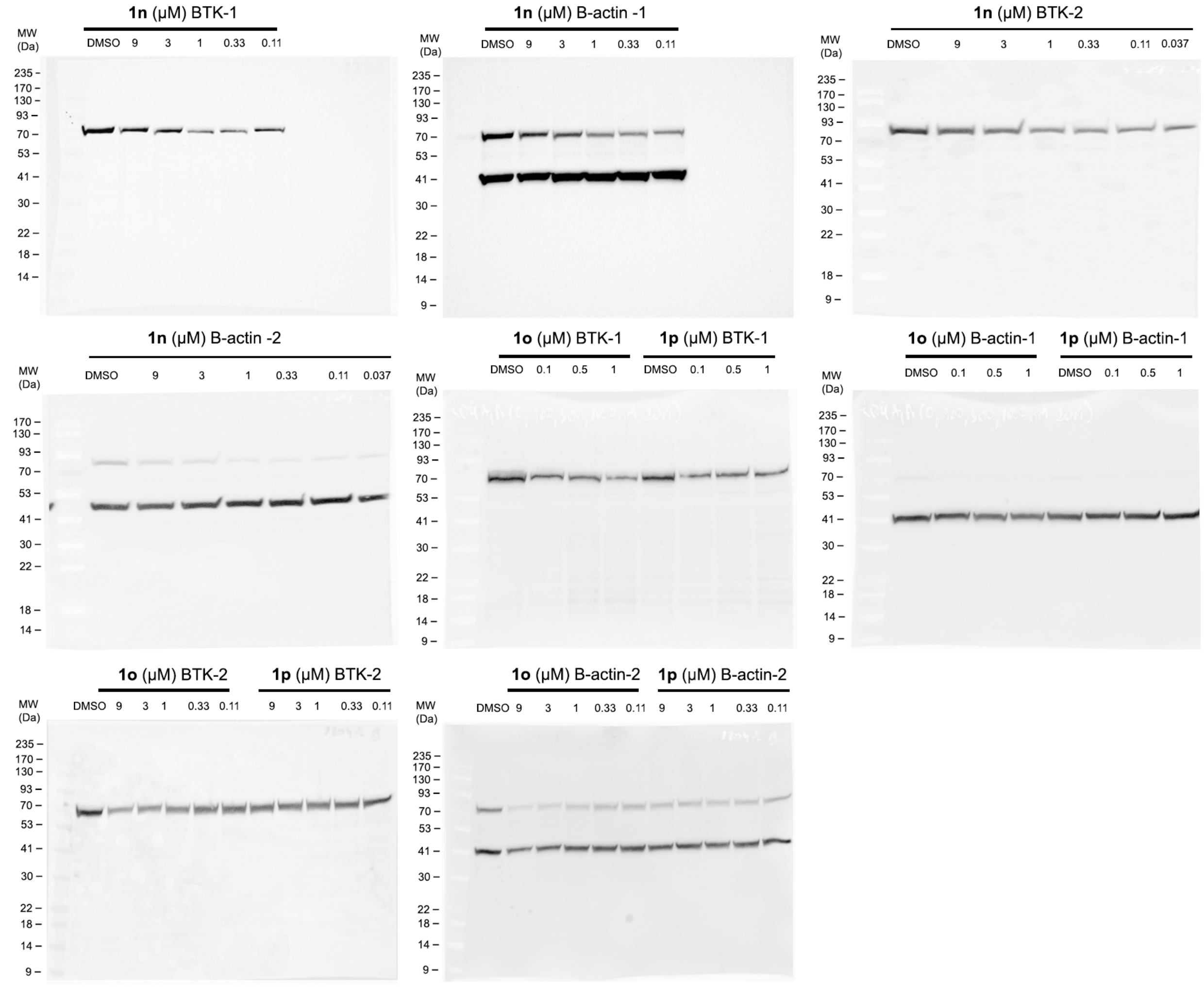


**Figure S19**. **CoLDR PROTACs mediated degradation of BTK.** Western blot evaluation of BTK levels in Mino cells in response to various concentrations of **1n**, **1o** and **1p** after 24 h of incubation (n=2).

## Supplementary Methods

### *MS/MS based proteomics*

100 µl of 5 µM Recombinant BTK kinase domain was incubated in 20 mM Tris with 50 µM of **1b** or DMSO. The compounds were then removed by methanol-chloroform (400 µL MeOH+100 µL $CHCl_3$+300 µL $H_2O$) precipitation of the protein. The dry pellet was dissolved in 50 µl of 50 mM Tris pH 8 + 5% SDS and heated to 95 °C for 6 min. The concentration of the protein was estimated using BCA assay (using BSA as the standard). 2 µg each sample were diluted to 15 µl with Tris 50 mM pH 8 + 5% SDS, reduced with DTT (0.75 µl of 0.1 M in 5% SDS/Tris 50 mM pH 8, 45 min 65 °C), cooled to room temperature, then alkylated with 0.75 µl of 0.2 M iodoacetamide in water (30 min room temperature in the dark). The protein was then isolated and trypsinized on s-traps (Protifi) according to the manufacturer's instructions. Triplicates were prepared for each molecule.

ULC/MS grade solvents were used for all chromatographic steps. Each sample was loaded using split-less nano-Ultra Performance Liquid Chromatography (10 kpsi nanoAcquity; Waters, Milford, MA, USA). The mobile phase was: A) $H_2O$ + 0.1% formic acid and B) acetonitrile + 0.1% formic acid. Desalting of the samples was performed online using a reversed-phase Symmetry C18 trapping column (180 µm internal diameter, 20 mm length, 5 µm particle size; Waters). The peptides were then separated using a T3 HSS nano-column (75 µm internal diameter, 250 mm length, 1.8 µm particle size; Waters) at 0.35 µL/min. Peptides were eluted from the column into the mass spectrometer using the following gradient: 4% to 30%B in 155 min, 35% to 90%B in 5 min, maintained at 90% for 5 min and then back to initial conditions.

The nanoUPLC was coupled online through a nanoESI emitter (10 µm tip; New Objective; Woburn, MA, USA) to a quadrupole orbitrap mass spectrometer (Q Exactive HFX, Thermo Scientific) using a FlexIon nanospray apparatus (Proxeon).

Data was acquired in data dependent acquisition (DDA) mode, using a Top10 method. MS1 resolution was set to 120,000 (at 200 m/z), mass range of 375-1650 m/z, AGC of 3e6 and maximum injection time was set to 60msec. MS2 resolution was set to 15,000, quadrupole isolation 1.7 m/z, AGC of 1e5, dynamic exclusion of 45 sec and maximum injection time of 60

msec.

***Proteomics analysis***

Analysis was done using MaxQuant 1.6.3.4. The sequence of BTK was used for the analysis. The digestion enzyme was set to Trypsin with a maximum number of missed cleavages of 0. Carbamidomethyl and the modification by the molecule were included as variable modifications on cysteine. The "Re-quantify" option was enabled. Contaminants were included. Peptides were searched with a minimum peptide length of 7 and a maximum peptide mass of 4,500 Da. "Second peptides" were enabled and "Dependent peptides" were disabled. The option "Match between runs" was enabled with a Match time window of 0.7 min and an alignment window of 20 min. An FDR of 0.01 was used for Protein FDR, PSM FDR and XPSM FDR. The triplicate measured for each compound (or for DMSO-treated protein) was analyzed separately.

Following MaxQuant analysis, only fully cleaved peptides were quantified and cysteine-containing peptides that were not modified by either iodoacetamide or compound were ignored. The intensity for each peptide was calculated as the average of the three triplicates. If the intensity was zero for one of the replicates the peptide was ignored. The intensities for the non-cysteine containing peptides were averaged for each data set and used to normalize the intensity of cysteine-containing peptides. Estimation of the extent of labeling of cysteine-containing peptides in the sequence was done by comparing the intensity of carbamidomethyl-modified peptides between the DMSO and molecule-treated samples. MS/MS spectra for the carbamidomethyl-modified and molecule modified peptides were extracted using Skyline.

***Identification of $K_d$ and $K_{inact}$ for 1b***

The BTK kinase domain was diluted to 200 nM in the buffer, and various concentrations (200, 300, 400, 500, 600, 1000 or 2000 nM) of **1b** were added separately by adding 1/250th volume from the stock solution. After the addition, the samples are put at 14 °C. The reaction mixtures, at various times, were injected into the LC/MS. For data analysis, the raw spectra were deconvoluted using a 20000:40000 Da window and 1 Da resolution. The labeling percentage for a compound was determined as the labeling of a specific compound (alone or together with other compounds) divided by the overall detected protein species. The % of unlabelled BTK was plotted against time and fitted to pseudo-first-order reaction kinetics formula (Fig. S5A). The obtained

rate constants were fitted to the Michaelis-Menten-type saturation curve to extrapolate $k_{inact}$ and $K_i$

***Surface Plasmon resonance of BTK labelled 1b***

Samples of 150 µl BTK (10.7 µM) were mixed with 1.5 µl of 2 mM Ibrutinib, 2 mM **1b**, or DMSO, giving 20 µM compound. After 1 hour labeling was tested using intact protein LC/MS and confirmed to be 100% for both compounds. The samples were then dialyzed against NaPi 25 mM pH 7.5, 100 mM NaCl, at 4 $^{o}$C as follows: Overnight against 50 ml- 7 hours against 50 ml- overnight against 45 ml. After samples were removed from dialysis, the amount of remaining compounds were estimated using the ratio between the MS signal of the compound relative to the MS signals of the 4 most intense protein peaks, before and after the dialysis. The results indicated that in the ibrutinib-treated sample, 95% of the excess ibrutinib was removed, and in the **1b**-treated sample, 90% of the excess **1b** was removed and 67% of the released Ibr-H was removed. To measure the protein concentration, the extinction coefficient of ibrutinib, IbrH and **1b** at 278 nm was estimated to be 4218 $M^{-1}cm^{-1}$ (based on measuring the absorbance of 400 µM ibrutinib), which was used to correct the extinction coefficients of the protein samples containing unremoved compounds. These values were used for measuring the concentrations of labeled BTK. The samples were diluted to 5 µM in NaPi 25 mM pH = 7.5, 100 mM NaCl and kept at 4 $^{o}$C for SPR.

Compound **1r** and control PEG linker (t-Boc-N-amido-PEG12-acid) was prepared at 10 µM in the Phosphate buffer (10 mM NaPi, pH 7) for immobilization. CM5 SPR chip was prepared on a Biacore T200 using standard coupling protocol (0.05 M NHS/0.2M EDC at a flow rate of 5 µl/min). amine-PEG linker was immobilized to flow channel 1 and **1r** was immobilized to other channels. Coupling was quenched with Ethanolamine and the chip was washed extensively with a running buffer (PBS+0.05% Tween 20). BTK binding experiments were then performed on a Biacore S200 instrument. Protein samples (BTK, BTK+**1b**, BTK+Ibrutinib) were diluted in running buffer to 50 nM, and then serial diluted 2-fold before injection on ligand chip. At the end of each cycle, the chip was regenerated with 2 injections of 4 mM NaOH to return to baseline sensogram. Specific

analyte binding was measured by RU (flow channel 4 - flow channel 1). Kinetic data was analyzed using Biacore S200 Evaluation software 1.1 (GE Healthcare).

***Pull-down proteomics experiments***

Mino cells were incubated for 1 h with DMSO, Ibrutinib followed by the incubation with 100 nM **1b** or DMSO. The cells were spun down, washed with cold PBS and frozen. Then, each sample was lysed in 200 ul of RIPA for 15 minutes on ice and centrifuged at 20000 g at 4 °C. The protein in the supernatant was quantified using BCA. For each sample, 250 °l of 1.65 mg/ml was prepared. At this point, 5 ul of 5 mM biotin azide and 9 ul of 100 mM CuSO4:THPTA complex were added. The click reaction was initiated by the addition of 7.5 ul of 150 mM sodium ascorbate, and the samples are incubated at room temperature for 1 hour. The samples were then precipitated with methanol: chloroform (1 ml methanol, 250 l chloroform, 750 l water), washed with 1 ml of methanol, and air dried. The dry pellet was resuspended in 1.2% SDS in PBS (250 l), sonicated as described before, and heated to 90 °C for 5 minutes. The samples were then diluted to 1.5 ml with PBS, and 50 ul of streptavidin agarose beads, prewashed with 0.2% SDS in PBS, were added, followed by 3 hour incubation at room temperature. Following the incubation, the beads were centrifuged 2 minutes 2000 g, and washed 4 times, with the following buffers (4 ml in each wash): 2% SDS; 0.1% sodium deoxycholate, 1% Triton X-100, 0.5 M NaCl, 1 mM EDTA, 50 mM HEPES pH = 7.5; 0.25 M NaCl, 0.5% IGEPAL, 0.5% sodium deoxycholate, 1 mM EDTA, 10 mM Tris pH = 8.1; 50 mM Tris pH = 7.4, 50 mM NaCl. The beads were then resuspended in Tris 50 mM pH = 8 and transferred to a clean Eppendorf tube.

At this point, the buffer was removed to leave a volume of 100 ul, and 100 ul of Tris 50 mM pH 8 + 10% SDS was added, and the samples were heated to 96 °C for 5 minutes. The samples were then centrifuged and the denatured, eluted proteins were transferred to new tubes. Then, 7.5 ul of 0.1 M DTT was added, and samples were incubated at 65 °C for 45 minutes. After the samples had cooled, 7.5 ul of iodoacetamide (0.2 M) were added, and the samples were incubated in the dark for 40 minutes at room temperature. At this point 1/10 volume of 12% phosphoric acid was added, and the samples were diluted 6-fold with 90% methanol + 50 mM ammonium bicarbonate. The samples were then loaded on s-trap micro columns (Protify) and the columns

were washed 3 times with 150 ul of 90% methanol + 50 mM ammonium bicarbonate. Then, 20 ul of 0.05 g/l of trypsin in 50 mM ammonium bicarbonate were added to the columns, and the samples were incubated at 47 °C for 90 minutes. Then 40 ul of 50 mM ammonium bicarbonate was added, followed by centrifugation and addition of 1 ul of 0.5 g/l trypsin to the eluate, which was incubated at 37C overnight. The column itself was then eluted using 40 ul of 0.2% formic acid and 40 ul 0.2% formic acid in 50% acetonitrile into a separate tube, which was kept at 4 °C. The two eluates were then combined and evaporated before running on LC/MS/MS.

Running samples on LC/MS/MS:

ULC/MS grade solvents were used for all chromatographic steps. Each sample was loaded using split-less nano-Ultra Performance Liquid Chromatography (10 kpsi nanoAcquity; Waters, Milford, MA, USA). The mobile phase was: A) $H_2O$ + 0.1% formic acid and B) acetonitrile + 0.1% formic acid. Desalting of the samples was performed online using a reversed-phase Symmetry C18 trapping column (180 µm internal diameter, 20 mm length, 5 µm particle size; Waters). The peptides were then separated using a T3 HSS nano-column (75 µm internal diameter, 250 mm length, 1.8 µm particle size; Waters) at 0.35 µL/min. Peptides were eluted from the column into the mass spectrometer using the following gradient: 4% to 30%B in 155 min, 35% to 90%B in 5 min, maintained at 90% for 5 min and then back to initial conditions. The nanoUPLC was coupled online through a nanoESI emitter (10 µm tip; New Objective; Woburn, MA, USA) to a quadrupole orbitrap mass spectrometer (Q Exactive HFX, Thermo Scientific) using a FlexIon nanospray apparatus (Proxeon). Data was acquired in data dependent acquisition (DDA) mode, using a Top10 method. MS1 resolution was set to 120,000 (at 200 m/z), mass range of 375-1650 m/z, AGC of $1e^6$ and maximum injection time was set to 60msec. MS2 resolution was set to 15,000, quadrupole isolation 1.7 m/z, AGC of $1e^5$, dynamic exclusion of 45 sec and maximum injection time of 60 msec.

Data analysis for pulldown:

The data were analyzed using MaxQuant 1.6.0.16. Human Proteome fasta file downloaded in January 2019 was used, and contaminants were included. The digestion enzyme was set to

Trypsin/P with a maximum number of missed cleavages of 2. Oxidation of methionine and N-terminal acetylation were included as variable modifications. The “Re-quantify” option was enabled. Carbamidomethyl (C2H3NO) was used as a fixed modification on cysteine. Contaminants were included. Peptides were searched with a minimum peptide length of 7 and a maximum peptide mass of 6500 Da. “Second peptides” was enabled, “Dependent peptides” were disabled and the option “Match between run” was enabled with a Match time window of 0.7 min and an alignment window of 20 min. An FDR of 0.01 was used for Protein FDR, PSM FDR and XPSM FDR. Proteins were identified and quantified based on the label-free quantification (LFQ) (REF: www.mcponline.org/content/13/9/2513.short) values reported by MaxQuant. Following MaxQuant analysis, proteins identified only through razor peptides or modified peptides, as well as common contaminants, were removed, and only proteins that gave at least 3 non-zero LFQ intensity values in at least one of the data sets were retained for analysis. Missing values were then replaced from a normal distribution.

***Degradation proteomics experiment (label free quantitative proteomics)***

Sample preparation: $10^6$ Mino cells were treated in four replicates with either compound or DMSO for 24 hours. Cells were then washed twice by centrifuge at 200 rcf, 4°C for 5 minutes, removing the supernatant and washed with ice cold PBS. Samples were then centrifuged at 200 rcf, 4°C for 5 minutes, then supernatant was removed and samples were frozen at -80°C. Samples were dispersed in 75 µl of 50 mM ammonium bicarbonate, and transferred to 1.8 mL glass vials. 75 µl of 10% SDS in 50 mM ammonium bicarbonate were added and the samples were heated to 96° C for 6 minutes. The samples were sonicated thoroughly in a sonication bath until the DNA was sheared as indicated by reduction in viscosity to level enabling easy pipettation. Total protein concentration was estimated using BCA assay and 30 μg from each sample was taken for the experiment.

The samples were reduced by the addition of 1/20 volume of 100 mM DTT and heating to 60° C for 45 minutes. The samples were cooled to room temperature, and 1/20 volume of 200 mM iodoacetamide was added and the reaction was performed in the dark for 30 minutes.

At this point, 1/10 volume of 12% phosphoric acid was added, followed by 6 volumes of 90% methanol in 50 mM ammonium bicarbonate. The samples were loaded on S-trap Micro columns (Protify), and washed 3 times with 90% methanol in 50 mM ammonium bicarbonate. The columns were spun without washing to dry the methanol. Then, 1 μg of trypsin (Promga) in 20 μl of 50 mM ammonium bicarbonate was added to each column, and they were incubated at 47° C for 1.5 hours. Then, 40 μl of 50 mM ammonium bicarbonate was added and the sample was eluted and another 0.5 μg of trypsin was added, followed by overnight incubation at 37° C. The columns themselves were further eluted with 40 μl 0.2% formic acid in water, followed by 40 μl of 0.2% formic acid in 50% acetonitrile:water, to elute strongly bound peptides. This elution was stored at 4° C and combined the next day with the first elution. The samples were then dried by SpeedVac and analyzed.

Liquid chromatography: ULC/MS grade solvents were used for all chromatographic steps. Each sample was analyzed using EASY-nLC 1200. The mobile phase was: A) H2O + 0.1% formic acid and B) 80% acetonitrile + 0.1% formic acid. The peptides were then separated using a PepMap RSLC C18 column (50 μm internal diameter, 150 mm length, 2.0 μm particle size, 100 Å pore size ; Thermo Scientific) at 0.3 μL/minute. Peptides were eluted from the column into the mass spectrometer using the following gradient: 5% to 38% B in 150 minutes, 38% to 100% B in 5 minutes, maintained at 100% for 5 minutes and then back to initial conditions.

Mass Spectrometry: The nanoUPLC was coupled online through a Nanospray Flex ion source to an Orbitrap Exploris 240 mass spectrometer (Thermo Scientific). Data was acquired in data dependent acquisition (DDA) mode, using a top-speed method with maximum cycle time of 3 sec. MS1 resolution was set to 120,000 (at 200 m/z), mass range of 375-1650 m/z, normalized AGC of 300% and maximum injection time was set to 20 msec. MS2 was performed by HCD in the Orbitrap with resolution set to 15,000, quadrupole isolation window of 1.4 m/z and normalized AGC of 50%.

Raw data were analyzed using the MaxQuant software suite 1.6.3.4 (www.maxquant.org) with the Andromeda search engine[1]. The higher-energy collisional dissociation (HCD) MS/MS spectra

were searched against an in silico tryptic digest of Homo sapiens proteins from the UniProt/Swiss-Prot sequence database (v. July 2019), including common contaminant proteins. All MS/MS spectra were searched with the following MaxQuant parameters: acetyl (protein N-terminus) and methionine oxidation as variable modifications; cysteine carbamidomethylation was set as fixed modification for all samples, except for the SDT samples in which case carbamidomethylation was set as variable modification; max 2 missed cleavages; and precursors were initially matched to 4.5 ppm tolerance and 20 ppm for fragment spectra. Peptide spectrum matches and proteins were automatically filtered to a 1% false discovery rate based on Andromeda score, peptide length, and individual peptide mass errors. Processing was conducted with a match between runs.

Proteins were identified and quantified based on at least two unique peptides and based on the label-free quantification (LFQ)[2] values reported by MaxQuant. 4 replicates were measured for each condition. Proteins were excluded from analysis if within all sets, there were less than three samples with directly detected and quantified protein. After this filtering step, the proteins were divided into 3 separate lists (Dataset S4): the first list included proteins that were identified and quantified in both DMSO treated and **1n**-treated samples. The second list included proteins identified and quantified only in DMSO-treated samples, while the third list included only proteins identified and quantified in **1n**-treated samples.

***Stability of BTK labelled 1i with GSH***

The BTK kinase domain was diluted to 2 µM in the buffer, and 2 µM of **1i** was added by adding 1/100th volume from a 200 µM solution to obtain BTK-**1i**. The resulting reaction mixture was added 1mM and 5 mM GSH and incubated for 18 h. The reaction mixtures, at room temperature for various times, were injected into the LC/MS. For data analysis, the raw spectra were deconvoluted using a 20000:40000 Da window and 1 Da resolution. The labeling percentage for a compound was determined as the labelling of a specific compound (alone or together with other compounds) divided by the overall detected protein species.
