# Supplementary material for: Site-Specific Labeling of Endogenous Proteins Using CoLDR Chemistry
Source: J Am Chem Soc. 2021 Nov 24;143(48):20095–108. doi: 10.1021/jacs.1c06167 (PMC8662641; doi:10.1021/jacs.1c06167)
Supplement: Supplementary file 2 — ja1c06167_si_002.pdf [file ja1c06167_si_002.pdf]

Site-Specific Labelling of Endogenous Proteins Using CoLDR Chemistry

Rambabu N. Reddi[1,*], Adi Rogel[1,*], Efrat Resnick[1], Ronen Gabizon[1], Pragati Kishore Prasad[1], Neta Gurwicz[2], Haim Barr[3], Ziv Shulman[2], Nir London[1,#]

[1] Dept. of Chemical and Structural Biology, The Weizmann Institute of Science, Rehovot, 7610001, Israel.

[2] Dept. of Immunology, The Weizmann Institute of Science, Rehovot, 7610001, Israel.

[3] Wohl Institute for Drug Discovery of the Nancy and Stephen Grand Israel National Center for Personalized Medicine, The Weizmann Institute of Science, Rehovot, 7610001, Israel

[*] Equal contribution

[#] To whom correspondence should be addressed, nir.london@weizmann.ac.il

Keywords: Bioconjugation, covalent inhibitors, CoLDR, protein half-life, PROTAC, Targeted protein degradation, Turn-on fluorescence, Ligand directed chemistry

Supporting Chemistry

1. **Materials and methods**: All reagents and solvents were obtained from commercial suppliers unless otherwise mentioned. Ibr-H (CAS 1022150-12-4) was purchased from BLD pharmatech. (5S)-4-(6-fluoro-7-(2-fluoro-6-hydroxyphenyl)-1-(2-isopropyl-4-methylpyridin-3-yl)-2-oxo-1,2-dihydropyrido[2,3-d]pyrimidin-4-yl)-5-methyl-2,3,4,5-tetrahydropyrazin-1-ium (AMG-510 amine) was purchased from Enamine. Compound **1q** was previously synthesized in our lab and used as such.[1] Deuterated solvents were purchased from Cambridge isotope laboratories and all other reagents are purchased from Sigma Aldrich and used as such without further purification.

   Aluminum-backed silica plates (Merck silica gel 60 F254) were used for thin layer chromatography (TLC) to monitor solution phase reactions. The purification of compounds was carried out on a combi flash chromatography and waters RP-HPLC with Prep C18 column. The ^{1}H-NMR and ^{13}CNMR spectra were recorded using a 400 MHz and 500 MHz Bruker advance spectrometers and were calibrated using residual undeuterated solvent as the internal references ($CDCl_3$: 7.26 ppm; DMSO-d6: 2.50 ppm; D_2O: 4.79 ppm and CD_3OD = 3.31 ppm). Chemical shifts are reported in ppm on a δ scale. The following abbreviations were used to explain NMR peak multiplicities: s = singlet, d = doublet, t = triplet, q = quartet, p = pentet, m = multiplet, br = broad. Most of the Ibrutinib derivatives appeared as a mixture of rotamers. The high-resolution mass spectra were recorded on Waters Xevo G2-XS QTof mass spectrometer using electrospray ionization time-of-flight (ESI-TOF) reflectron experiments.

2. **Abbreviations.** Acetonitrile (ACN), dichloromethane (DCM), N,N'-diisopropylethylamine (DIPEA), N,N'-dimethylformamide (DMF), Ethyl acetate (EtOAc), dimethyl sulfoxide (DMSO), 1-Ethyl-3-(3-dimethylaminopropyl) carbodiimide (EDC), 1-[bis(dimethylamino)methylene]-1H-1,2,3- triazolo[4,5-b]pyridinium3-oxid hexafluorophosphate (HATU), methanol (MeOH), Phosphate buffer saline (PBS), high-performance liquid chromatography (HPLC), trifluoroacetic acid (TFA), 1,4-diazabicyclo[2.2.2]octane (DABCO), diisopropylethyl amine (DIPEA)

(R)-2-((3-(4-amino-3-(4-phenoxyphenyl)-1H-pyrazolo[3,4-d]pyrimidin-1-yl)piperidin-1-yl)methyl)acrylic acid (Ibr-acid)

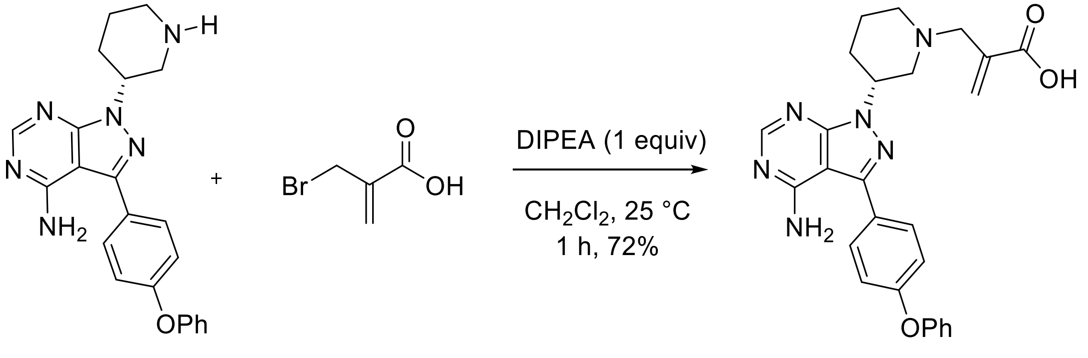

To a stirred solution of Ibr-H (387 mg, 1 mmol) in anhydrous DCM (6 mL), DIPEA (178 µL, 1 mmol) and 2-(bromomethyl)acrylic acid (162 mg, 1 mmol) were added at 25 °C. The reaction mixture was stirred at room temperature for 1 h. After completion of the reaction (as monitored by LC-MS), CH_2Cl_2 was concentrated *in vacuo* and the crude carboxylic acid was purified using combi flash column chromatography using MeOH:EtOAc (2:8) as eluent to give Ibr-acid in 335 mg (yield = 72%)

^{1}H NMR (500 MHz, CD_3OD): δ 1.99 (br. s., 2H), 2.19 (br. s., 1H), 2.24 (br. s., 1H), 3.74 (d, *J* = 16.2 Hz, 2H), 4.10-4.20 (d, *J* = 13.2 Hz, 3H), 5.33 (br. s., 1H), 6.29 (s, 1H), 6.68 (s, 1H), 7.10 (d, *J* = 7.7 Hz, 2H), 7.13-7.23 (m, 4H), 7.42 (t, *J* = 8.0 Hz, 2H), 7.74 (d, *J* = 8.4 Hz, 2H), 8.34 (s, 1H); **^{13}C NMR** (126 MHz, CD_3OD): δ ppm 21.6, 28.8, 52.0, 53.7, 55.3, 59.7, 99.2, 120.1, 120.8, 125.4, 127.9, 131.3, 131.5, 131.5, 136.5, 147.4, 154.7, 155.6, 157.9, 160.5, 168.7; ESI-MS (m/z): calculated for $C_{26}H_{27}N_6O_3$ [M+H]+: 471.21; found: [M+H]+:471.45.

Ethyl (R)-2-((3-(4-amino-3-(4-phenoxyphenyl)-1H-pyrazolo[3,4-d]pyrimidin-1-yl)piperidin-1-yl)methyl)acrylate (1a)

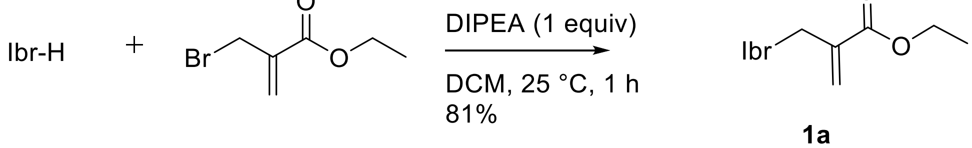

To a stirred solution of Ibr-H (38.7 mg, 0.1 mmol) in anhydrous DCM (1 mL), DIPEA (17.3 µL, 0.1

mmol) and ethyl bromo methacrylate (14.6 µL, 0.1 mmol) were added at 25 °C. The reaction mixture was stirred at room temperature for 1 h. After completion of the reaction (as monitored by LC-MS), the reaction mixture was evaporated under *vacuo* and the crude product was purified by preparative HPLC using water:ACN (0.1% TFA) solvent gradient to afford **1a** as colorless solid in 40 mg (yield = 81 %).

^{1}H NMR (500 MHz, CD_3OD): δ 1.28 (t, *J* = 6.7 Hz, 3H), 1.98-2.19 (m, 2H), 2.27 (br. s., 2H), 3.81 (d, *J* = 10.2 Hz, 1H), 4.08-4.18 (m, 2H), 4.18-4.35 (m, 3H), 5.44 (br. s., 1H), 6.33 (s, 1H), 6.68 (s, 1H), 7.11 (d, *J* = 8.0 Hz, 2H), 7.14-7.23 (m, 3H), 7.37-7.46 (m, 2H), 7.76 (br. s., 2H), 8.44 (s, 1H); **^{13}C NMR** (126 MHz, CD_3OD): δ 14.5, 28.6, 36.9, 52.4, 53.8, 55.5, 59.4, 63.3, 98.7, 120.1, 120.9, 125.5, 126.9, 131.3, 131.7, 136.8, 148.7, 153.8, 157.8, 160.9, 167.0; **HR-MS** (m/z): Calculated for $C_{28}H_{31}N_6O_3$ $[M+H]^+$: 499.2458; Found $[M+H]^+$: 499.2454.

(R)-N-(2-(2-((3-(4-amino-3-(4-phenoxyphenyl)-1H-pyrazolo[3,4-d]pyrimidin-1-yl)piperidin-1-yl)methyl)acrylamido)ethyl)-4-formylbenzamide (1b)

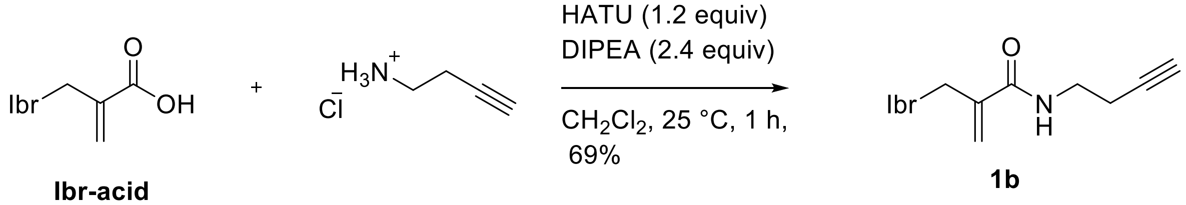

Ibr-acid **1b**

To a stirred solution of carboxylic acid (23.5 mg, 0.05 mmol) in CH_2Cl_2 (1 mL), HATU (23 mg, 0.06 mmol), DIPEA (20.4 µL, 0.12 mmol) and but-3-yn-1-amine hydrochloride (6.3 mg, 0.06 mmol) were added at 25 °C. The reaction mixture was stirred at room temperature for 1 h. After completion of the reaction (as monitored by LC-MS), water (1 mL) was added. The aqueous layer was extracted with CH_2Cl_2 (3 × 3 mL). The combined organic layer was concentrated *in vacuo* and the crude product was purified by preparative HPLC using water:ACN (0.1% formic acid) solvent gradient to afford white solid **1b** in 18 mg (69% yield).

^{1}H NMR (400 MHz, CD_3OD): δ 2.09 (br. s., 1H), 2.21 (br. s., 1H), 2.32 (br. s., 2H), 2.45 (br. s., 2H), 3.26 (br. s., 1H), 3.44 (br. s., 2H), 3.82 (d, *J* = 11.2 Hz, 2H), 4.12 (d, *J* = 12.8 Hz, 2H), 4.26 (d, *J* = 13.0 Hz, 1H), 5.56 (br. s., 1H), 6.14 (s, 1H), 6.35 (s, 1H), 7.21 (d, *J* = 7.7 Hz, 2H), 7.28 (s, 3H), 7.48-7.56 (m, 2H), 7.98 (br. s., 2H), 8.52 (s, 1H): **^{13}C NMR** (100 MHz, CD_3OD): δ 19.7, 28.4, 39.8, 52.0, 53.2, 55.6, 61.3, 71.2, 82.1, 98.8, 120.1, 120.9, 125.4, 127.1, 129.6, 131.3, 131.9, 133.7, 148.4, 152.0,

153.9, 156.8, 157.9, 160.8, 169.1; **HR-MS** (m/z): Calculated for $C_{30}H_{32}N_7O_2$ $[M+H]^+$: 522.2617; Found $[M+H]^+$: 522.2620.

(R)-2-((3-(4-amino-3-(4-phenoxyphenyl)-1H-pyrazolo[3,4-d]pyrimidin-1-yl)piperidin-1-yl)methyl)-N-(4-ethynylphenyl)acrylamide (1c):

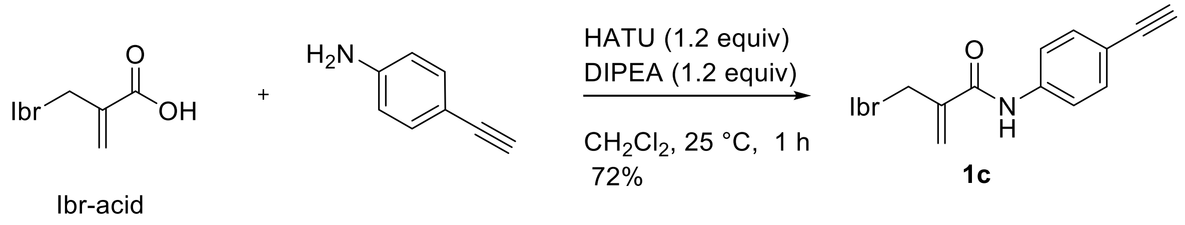

To a stirred solution of carboxylic acid (23.5 mg, 0.05 mmol) in CH_2Cl_2 (1 mL), HATU (23 mg, 0.06 mmol), DIPEA (10.4 µL, 0.06 mmol) and 4-ethynylaniline (7.02 mg, 0.06 mmol) were added at 25 °C. The reaction mixture was stirred at room temperature for 1 h. After completion of the reaction (as monitored by LC-MS), water (1 mL) was added. The aqueous layer was extracted with CH_2Cl_2 (3 × 3 mL). The combined organic layer was concentrated *in vacuo* and the crude product was purified by preparative HPLC using water:ACN (0.1% formic acid) solvent gradient to afford white solid **1c** in 20.4 mg (72% yield).

^{1}H NMR (500 MHz, CD_3OD): δ ppm 2.00 (br. s., 1H), 2.06-2.18 (m, 1H), 2.24 (br. s., 2H), 3.22 (br. s., 1H), 3.51 (s, 1H), 3.76 (d, *J* = 11.8 Hz, 1H), 3.85 (br. s., 1H), 4.10 (d, *J* = 12.8 Hz, 2H), 4.23 (d, *J* = 12.9 Hz, 1H), 5.48 (br. s., 1H), 6.19 (s, 1H), 6.47 (s, 1H), 7.13 (t, *J* = 8.2 Hz, 4H), 7.21 (t, *J* = 7.4 Hz, 1H), 7.36 (d, *J* = 7.8 Hz, 2H), 7.43 (t, *J* = 7.8 Hz, 2H), 7.53 (br. s., 2H), 7.82 (br. s., 2H), 8.39 (s, 1H); **^{13}C NMR** (125 MHz, CD_3OD): δ ppm 20.1, 28.4, 36.3, 51.8, 53.4, 55.8, 61.4, 78.9, 84.3, 98.9, 120.0, 121.0, 121.5, 125.6, 127.2, 131.1, 131.4, 131.8, 133.8, 139.7, 148.1, 153.0, 154.1, 157.7, 160.8, 167.6. **HR-MS** (m/z): Calculated for $C_{34}H_{32}N_7O_2$: $[M+H]^+$: 570.6770; Found $[M+H]^+$: 570.6775.

Synthesis of pre-1d:

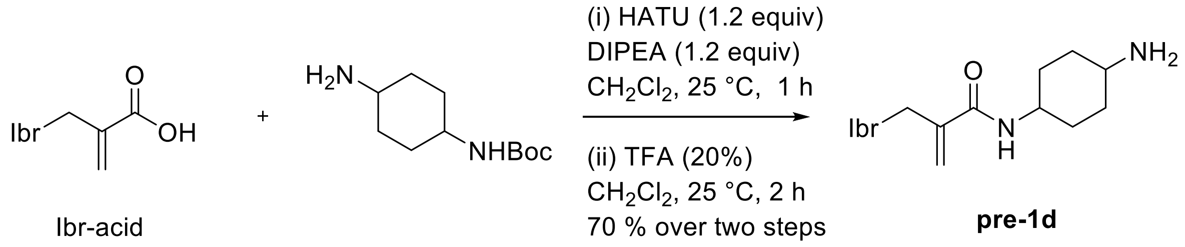

To a stirred solution of carboxylic acid (47 mg, 0.1 mmol) in CH_2Cl_2 (5 mL), HATU (45.6 mg, 0.12 mmol), DIPEA (20.9 µL, 0.12 mmol) and *tert*-butyl (4-aminocyclohexyl) carbamate (25.6 mg, 0.12

mmol) were added at 25 °C. The reaction mixture was stirred at room temperature for 1 h. After completion of the reaction (as monitored by LC-MS), 3 mL water was added. The aqueous layer was extracted with CH_2Cl_2 (3 × 5 mL). The combined organic layers were concentrated *in vacuo* and the crude product was dissolved in 20% TFA in DCM and allowed to stir at 25 °C for 2 h. The reaction mixture was concentrated and purified by using flash column chromatography in ethyl acetate:methanol (7:3) solvent system to get pure amine in 39.6 mg (yield = 70 %).

^{1}H NMR (400 MHz, CD_3OD): δ 1.64 (br. s., 3H), 1.94 - 2.06 (m, 2H), 2.10 (br. s., 3H), 2.25 (br. s., 3H), 2.82 (s, 1H), 2.94 - 3.04 (m, 2H), 3.08 (s, 1H), 3.41 (br. s., 1H), 3.47 (br. s., 1H), 3.77 (dd, *J* = 12.9, 3.6Hz, 1H), 3.97 (d, *J* = 13.2 Hz, 2H), 4.15 (d, *J* = 13.0Hz, 1H), 4.24 (br. s., 1H), 4.54 (br. s., 1H), 5.36 (br. s., 1H), 5.81 (s, 1H), 6.02 (s, 1H), 7.03-7.24 (m, 5H), 7.37-7.49 (m, 2H), 7.79 (d, *J* = 8.4 Hz, 2H), 8.32 (s, 1H); **^{13}C NMR** (100 MHz, CD_3OD): δ 28.8, 30.7, 39.0, 56.0, 61.7, 99.2, 119.9, 120.7, 122.1, 125.4, 128.2, 129.7, 131.3, 131.8, 136.4, 141.0, 146.9, 151.7, 154.8, 156.1, 158.0, 159.5, 160.3, 170.1.

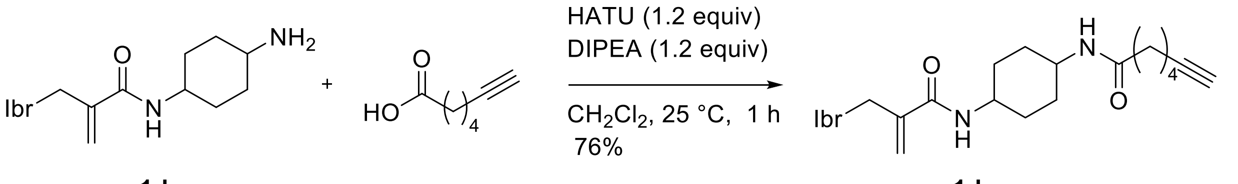

To a stirred solution of carboxylic acid (7.56 mg, 0.06 mmol) in CH_2Cl_2 (5 mL), HATU (23 mg, 0.06 mmol), DIPEA (10.4 µL, 0.06 mmol) and **pre-1d** (28 mg, 0.05 mmol) were added at 25 °C. The reaction mixture was stirred at room temperature for 1 h. After completion of the reaction (as monitored by LC-MS), 1 mL water was added. The aqueous layer was extracted with CH_2Cl_2 (3 × 3 mL). The combined organic layers were concentrated *in vacuo* and the crude product was purified by preparative HPLC using water:ACN (0.1% TFA) solvent gradient to afford **1d** in 25.6 mg (yield = 76 %).

^{1}H NMR (500 MHz, CD_3OD): δ 1.33-1.48 (m, 2H), 1.48-1.61 (m, 3H), 1.66-1.80 (m, 2H), 1.90 (br. s., 2H), 1.94-2.11 (m, 2H), 2.22 (br. s., 8H), 2.98 (d, *J* =14.4Hz, 1H), 3.13 (br. s., 1H), 3.18 (s, 1H), 3.62-3.79 (m, 1H), 3.79-4.00 (m, 3H), 4.09 (br. s., 2H), 4.12-4.22 (m, 1H), 4.37 (br. s., 1H), 5.43 (br. s., 1H), 5.82 (br. s., 1H), 6.01 (br. s., 1H), 7.12 (d, *J* = 8.4 Hz, 2H), 7.17 (d, *J* = 8.7 Hz, 2H), 7.23 (t,

J = 7.4 Hz, 1H), 7.39-7.50 (m, 2H), 7.86 (br. s., 2H), 8.37 (s, 1H); **^{13}C NMR** (125 MHz, CD_3OD): δ 17.4, 18.5, 24.7, 27.8, 30.8, 31.7, 35.1, 40.5, 46.3, 54.4, 60.4, 68.4, 83.2, 97.6, 102.7, 118.3, 118.6, 119.4, 124.0, 129.8, 130.4, 153.8, 156.4, 159.1, 168.3, 173.9. **HR-MS** (m/z): Calculated for $C_{39}H_{47}N_8O_3$: $[M+H]^+$: 675.3771; Found $[M+H]^+$: 675.3777.

(R)-2-((3-(4-amino-3-(4-phenoxyphenyl)-1H-pyrazolo[3,4-d]pyrimidin-1-yl)piperidin-1-yl)methyl)-N-(2-aminoethyl)acrylamide (1e):

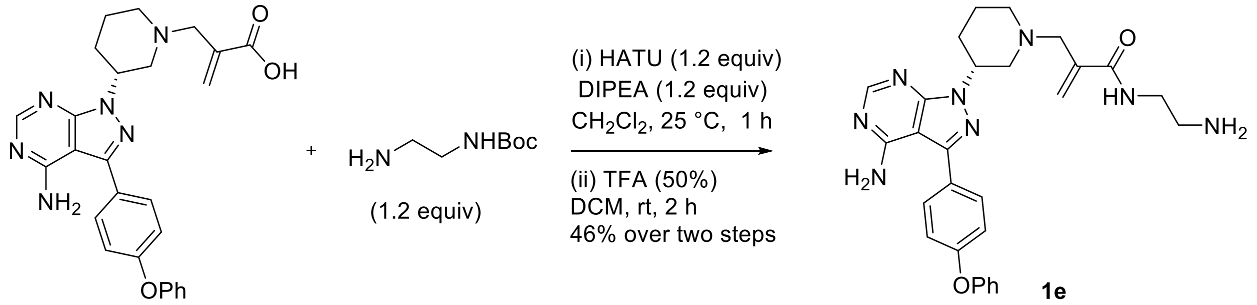

To a stirred solution of carboxylic acid (188 mg, 0.4 mmol) in CH_2Cl_2 (5 mL), HATU (182 mg, 0.48 mmol), DIPEA (85 µL, 0.48 mmol) and *N*-Boc ethelene diamine (77 mg, 0.48 mmol) were added at 25 °C. The reaction mixture was stirred at room temperature for 1 h. After completion of the reaction (as monitored by LC-MS), 3 mL water was added. The aqueous layer was extracted with CH_2Cl_2 (3 × 5 mL). The combined organic layers were concentrated *in vacuo* and the crude product was dissolved in 50% TFA in DCM and allowed to stir at 25 °C for 2 h. The reaction mixture was concentrated and purified by using flash column chromatography in ethyl acetate:methanol (7:3) solvent system to get pure amine **1e** in 94 mg (yield = 46.0%).

^{1}H NMR (400 MHz, CD_3OD): δ 2.12 (br. s., 2H), 2.35 (br. s., 2H), 3.10 (br. s., 0.5H), 3.16-3.24 (m, 2H), 3.34 (br. s., 0.5H), 3.59-3.66 (m, 2H), 3.70-3.72 (m, 1H), 3.85 (br. s., 2H), 4.16 (d, J = 12.8 Hz, 1H), 4.27 (d, J = 12.8 Hz, 1H), 5.50 (br. s., 1H), 6.22 (s, 1H), 6.43 (s, 1H), 7.21 (d, J = 7.7 Hz, 2H), 7.25-7.35 (m, 3H), 7.49-7.57 (m, 2H), 7.88 (br. s., 2H), 8.51 (s, 1H); ^{13}C NMR (101 MHz, CD_3OD) δ 28.6, 38.6, 40.5, 40.9, 52.7, 53.3, 55.4, 60.7, 98.9, 116.7, 119.6, 120.1, 120.8, 125.5, 127.5, 128.4, 130.8, 131.3, 131.7, 133.9, 135.5, 148.1, 154.2, 157.9, 160.7, 169.7. **HR-MS** (m/z): Calculated for $C_{28}H_{33}N_8O_2$ $[M+H]^+$: 513.2726; Found $[M+H]^+$: 513.2722.

Synthesis of 1f:

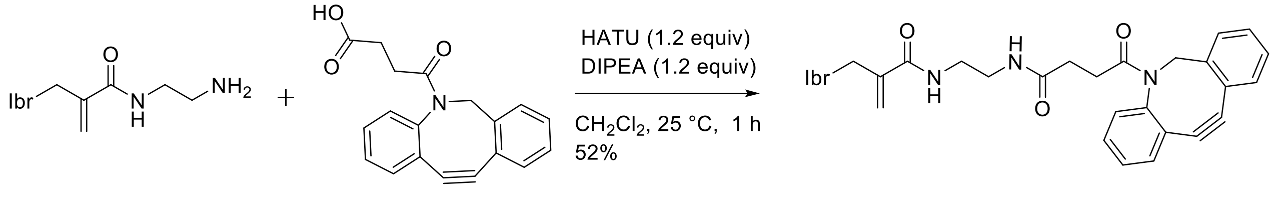

1e 1f

To a stirred solution of DBCO carboxylic acid (7.6 mg, 0.025 mmol) in CH_2Cl_2 (1 mL), HATU (11.5 mg, 0.03 mmol), DIPEA (5.2 µL, 0.03 mmol) and amine **1e** (13 mg, 0.05 mmol) were added at 25 °C. The reaction mixture was stirred at room temperature for 1 h. After completion of the reaction (as monitored by LC-MS), the reaction mixture was quenched with H_2O (1 mL). The aqueous layer was extracted with CH_2Cl_2 (3 × 1 mL) and the combined organic layers were evaporated under *vacuo*. The crude product was purified by preparative HPLC using water:ACN (0.1% TFA) solvent gradient to afford DBCO alkyne **1f** as colorless solid in 10.3 mg (yield = 52 %).

*15 % of the **1d** was hydrolyzed at DBCO amide site during HPLC purification.

^{1}H NMR (500 MHz, CD_3OD): δ 1.94 (br. s., 2H), 2.08 (br. s., 2H), 2.21 (br. s., 3H), 2.61-2.72 (m, 1H), 3.02-3.19 (m, 3H), 3.70 (d, *J* = 14.0 Hz, 2H), 3.76 (br. s., 1H), 3.99 (br. s., 1H), 4.07 (br. s., 1H), 4.15 (br. s., 1H), 5.09 (d, *J* = 14.0 Hz, 1H), 5.44 (br. s., 1H), 6.00 (br. s., 1H), 6.18 (s, 1H), 7.11 (d, *J* = 7.8 Hz, 2H), 7.16 (d, *J* = 8.5 Hz, 2H), 7.18-7.26 (m, 2H), 7.32 (br. s., 2H), 7.39-7.52 (m, 5H), 7.56 (d, *J* = 7.3 Hz, 1H), 7.61 (br. s., 1H), 7.86 (br. s., 1H), 8.41 (br. s., 1H); **^{13}C NMR** (126 MHz, CD_3OD): δ 20.0, 28.4, 31.4, 32.0, 40.6, 52.0, 53.1, 55.7, 56.9, 101.5, 108.9, 120.0, 120.9, 123.9, 124.5, 125.5, 126.7, 128.3, 129.0, 129.3, 129.9, 130.2, 130.7, 131.3, 132.0, 133.5, 149.7, 152.7, 157.9, 160.7, 174.2, 177.2; **HR-MS** (m/z): Calculated for $C_{47}H_{46}N_9O_4$ $[M+H]^+$: 800.3673; Found $[M+H]^+$: 800.3684.

(R)-2-((3-(4-amino-3-(4-phenoxyphenyl)-1H-pyrazolo[3,4-d]pyrimidin-1-yl)piperidin-1-yl)methyl)-N-(2-(3-(3',6'-dihydroxy-3-oxo-3H-spiro[isobenzofuran-1,9'-xanthen]-5-yl)thioureido)ethyl)acrylamide (1g):

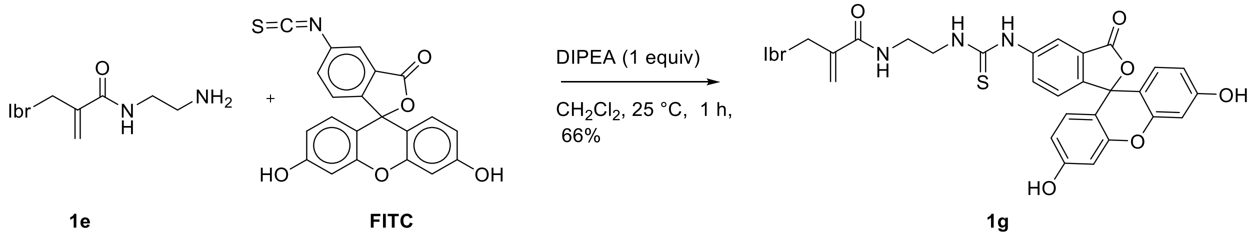

1e FITC 1g

To a stirred solution of **1e** (0.05 mmol, 26 mg) in anhydrous DCM (1 mL), DIPEA (9 µL, 0.05 mmol) and FITC (19 mg, 0.05 mmol) were added at 25 °C. The reaction mixture was stirred at room temperature for 1 h. After completion of the reaction (as monitored by LC-MS), the reaction mixture was quenched with H_2O (2 mL). The aqueous layer was extracted with CH_2Cl_2 (3 × 2 mL) evaporated under *vacuo*. The crude product was purified by preparative HPLC using water:ACN (0.1% TFA) solvent gradient to afford **1g** as yellow color solid in 29.1 mg (yield = 66 %).

^{1}H NMR (400 MHz, CD_3OD): δ 1.83 (br. s., 2H), 2.15 (br. s., 2H), 3.12 (d, *J* = 12.8 Hz, 1H), 3.39 (br. s., 1H), 3.54-3.78 (m, 4H), 4.00 (br. s., 3H), 4.19 (m, 1H), 5.42 (br. s., 1H), 6.07 (s, 1H), 6.35 (s, 1H), 6.47 (br. s., 1H), 6.51-6.59 (m, 2H), 6.64 (s, 1H), 6.67 (s, 1H), 6.72 (d, *J* = 2.2 Hz, 1H), 6.84 (br. s., 1H), 7.08 (t, *J* = 9.0 Hz, 4H), 7.19 (t, *J* = 7.5 Hz, 1H), 7.36-7.46 (m, 2H), 7.71 (d, *J* = 8.1 Hz, 1H), 7.78 (br. s., 2H), 7.95 (br. s., 1H), 8.41 (br. s., 1H); **^{13}C NMR** (100 MHz, CD_3OD): δ 19.9, 28.3, 40.8, 45.0, 51.9, 53.0, 55.8, 61.4, 98.7, 103.7, 111.9, 114.2, 114.3, 119.9, 120.0, 121.0, 125.5, 126.4, 129.4, 130.5, 130.7, 131.3, 132.0, 133.6, 142.1, 148.1, 152.6, 154.0, 154.6, 157.0, 157.7, 160.9, 162.2, 162.3, 169.2, 170.9, 183.2. **HR-MS** (m/z): Calculated for $C_{49}H_{44}N_9O_7S$ $[M+H]^+$: 902.3084; Found $[M+H]^+$: 902.3082.

(R)-2-((3-(4-amino-3-(4-phenoxyphenyl)-1H-pyrazolo[3,4-d]pyrimidin-1-yl)piperidin-1-yl)methyl)-N-(2-(2-((9-(diethylamino)-5-oxo-5H-benzo[a]phenoxazin-2-yl)oxy)acetamido)ethyl)acrylamide (1h):

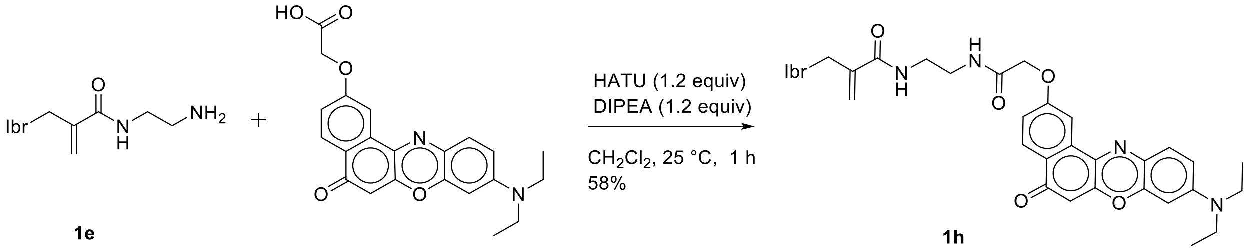

1e

1h

To a stirred solution of carboxylic acid (Nile Red carboxylic acid was synthesized using the literature report[2], 19 mg, 0.05 mmol) in CH_2Cl_2 (1 mL), HATU (23 mg, 0.06 mmol), DIPEA (10.4 µL, 0.06 mmol) and amine **1e** (13 mg, 0.05 mmol) were added at 25 °C. The reaction mixture was stirred at room temperature for 1 h. After completion of the reaction (as monitored by LC-MS), the reaction mixture was quenched with H_2O (2 mL). The aqueous layer was extracted with CH_2Cl_2 (3 × 3 mL) evaporated under *vacuo*. The crude product was purified by preparative HPLC using water:ACN (0.1% TFA) solvent gradient to afford **1h** as blue colour solid in 25.7 mg (yield = 58 %).

^{1}H NMR (500 MHz, CD_3OD): δ 1.29 (t, *J* = 6.6 Hz, 6H), 1.91 (br. s., 2H), 2.12 (br. s., 2H), 3.10 (br.

s., 1H), 3.47 (br. s., 4H), 3.57 (d, J =6.2 Hz, 5H), 3.71 (br. s., 2H), 3.92-4.10 (m, 2H), 4.19 (d, J = 12.5 Hz, 1H), 4.57 (br. s., 1H), 5.29 (br. s., 1H), 6.05 (br. s., 1H), 6.20 (d, J = 8.5 Hz, 1H), 6.24 (br. s., 1H), 6.62 (d, J = 8.3 Hz, 1H), 6.88 (br. s., 1H), 7.09 (d, J = 7.0 Hz, 4 H), 7.14-7.19 (m, 1H), 7.21 (br. s., 1H), 7.42 (t, J = 7.5 Hz, 2H), 7.56 (br. s., 2H), 7.75 (br. s., 2H), 7.91 (d, J = 12.9 Hz, 1H), 8.03 (br. s., 1H), 8.29 (br. s., 1 H); **^{13}C NMR** (126 MHz, CD3OD δ ppm 13.0, 19.9, 28.4, 40.5, 40.8, 46.4, 52.2, 52.2, 52.9, 55.6, 61.5, 68.4, 97.4, 98.7, 104.8, 108.2, 112.6, 119.0, 119.9, 120.9, 125.5, 126.8, 127.0, 128.7, 129.7, 131.3, 131.9, 132.7, 135.5, 138.6, 148.7, 153.6, 154.1, 157.8, 160.7, 161.7, 169.6, 184.0.; **HR-MS** (m/z): Calculated for $C_{50}H_{51}N_{10}O_6$ $[M+H]^+$: 887.3993; Found $[M+H]^+$: 887.3994.

(S)-2-((3-(4-amino-3-(4-phenoxyphenyl)-1H-pyrazolo[3,4-d]pyrimidin-1-yl)piperidin-1-yl)methyl)-N-(2-(3-(5,5-difluoro-7,9-dimethyl-5H-5l4,6l4-dipyrrolo[1,2-c:2',1'-f][1,3,2]diazaborinin-3-yl)propanamido)ethyl)acrylamide (1i)

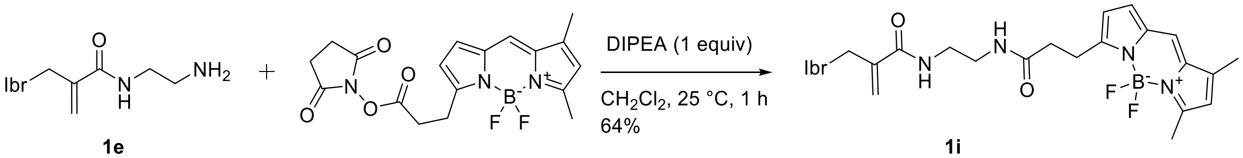

1e **1i**

To a stirred solution of **1e** (6.4 mg, 0.0125 mmol) in CH_2Cl_2 (0.5 mL), BODIPY NHS ester (4.9 mg, 0.0125 mmol), DIPEA (2.2 µL, 0.0125 mmol) were added at 25 °C. The reaction mixture was stirred at room temperature for 1 h. After completion of the reaction (as monitored by LC-MS), the reaction mixture was concentrated under *vacuo* and the crude product was purified by preparative HPLC using water:ACN (0.1% TFA) solvent gradient to afford **1i** as bright yellow colour solid in 6.2 mg (yield = 64 %).

^{1}H NMR (400 MHz, CD_3OD): δ 1.32-1.37 (m, 2H), 1.85-2.02 (br. s., 2H), 2.21 (d, J=7.5 Hz, 2H), 2.28 (s, 3H), 2.46 (br. s., 4H), 3.10 (t, J = 7.7 Hz, 2H), 3.14 (dt, J = 3.3, 1.7 Hz, 1H), 3.49 (dt, J = 3.2, 1.7 Hz, 1H), 3.58-3.73 (m, 3H), 3.77 (s, 1H), 3.95-4.03 (m, 1H), 4.03- 4.11 (m, 1H), 4.15 (d, J = 12.8 Hz, 1H), 5.36-5.46 (m, 1H), 6.02 (s, 1H), 6.21 (s, 2H), 6.25 (br. s., 1H), 6.96 (br. s., 1H), 7.11 (d, J = 7.7 Hz, 2H), 7.18 (d, J = 9.5 Hz, 2H), 7.20-7.25 (m, 1H), 7.35-7.50 (m, 3H), 7.52-7.64 (m, 1H), 7.80 - 7.92 (m, 1H), 8.38 (br. s., 1H); **^{13}C NMR** (100 MHz, CD_3OD): δ 11.3, 15.0, 20.0, 25.7, 28.4, 36.1, 41.0, 52.1, 53.2, 55.5, 61.2, 117.5, 120.0, 120.9, 121.6, 125.5, 125.9, 127.4, 129.7, 131.3, 132.0, 134.9, 136.7, 140.5, 146.2, 153.5, 154.2, 157.9, 158.4, 160.7, 161.6, 169.3, 175.2; **HR-MS** (m/z): Calculated for $C_{42}H_{46}BF_2N_{10}O_3$ $[M+H]^+$: 787.3815; Found $[M+H]^+$: 787.3828.

Synthesis of **1j:**

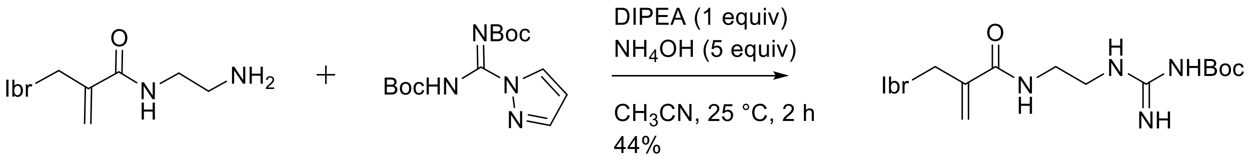

To a stirred solution of amine (26 mg, 0.05 mmol) in anhydrous ACN (1 mL), DIPEA (8.5 µL, 0.05 mmol) and N, N'-Di Boc-1H-pyrazole-1-carboxamidine (18.6 mg, 0.06 mmol) and NH_4OH (28% in water, 31 µL) were added at 25 °C. The reaction mixture was stirred at room temperature for 2 h. After completion of the reaction (as monitored by LC-MS), the reaction mixture was evaporated under *vacuo*. The crude product was purified by preparative HPLC using water:ACN (0.1% TFA) solvent gradient to afford methacrylamide (**1j**) as colorless solid in 14.4 mg (yield = 44 %). One of the Boc group was hydrolyzed in HPLC during purification.

^{1}H NMR (400 MHz, CD_3OD): δ 1.56 (br. s., 9H), 2.09 (br. s., 2H), 2.34 (br. s., 2H), 3.20-3.25 (m, 1H), 3.47-3.65 (m, 5H), 3.82 (d, *J* = 9.2 Hz, 2H), 4.09-4.18 (m, 1H), 4.27 (d, *J* = 12.3 Hz, 1H), 5.49 (br. s., 1H), 6.20 (br. s., 1H), 6.39 (s, 1H), 7.21 (d, *J* = 7.7 Hz, 2H), 7.24-7.34 (m, 3H), 7.47-7.59 (m, 2H), 7.91 (br. s., 2H), 8.48 (s, 1H). **^{13}C NMR** (100 MHz, CD_3OD): δ 26.7, 27.1, 37.4, 38.3, 40.2, 40.7, 51.0, 51.7, 54.1, 59.3, 84.5, 97.5, 118.5, 119.2, 123.9, 126.3, 128.9, 129.8, 130.3, 148.8, 152.1, 153.0, 154.4, 156.4, 159.0, 167.9, 168.2; **HR-MS** (m/z): Calculated for $C_{34}H_{43}N_{10}O_4$ $[M+H]^+$: 655.3469; Found $[M+H]^+$: 655.3473.

(R)-N-(2-(2-((3-(4-amino-3-(4-phenoxyphenyl)-1H-pyrazolo[3,4-d]pyrimidin-1-yl)piperidin-1-yl)methyl)acrylamido)ethyl)-4-formylbenzamide (1k)

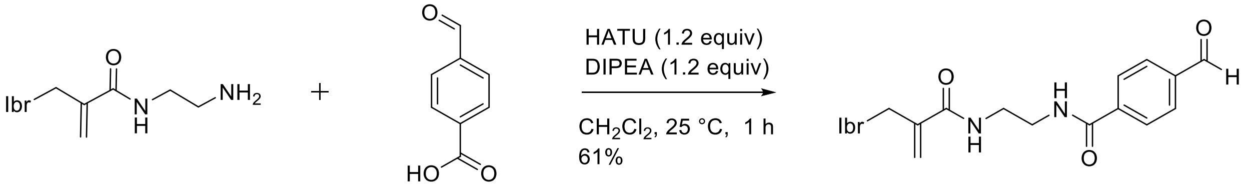

To a stirred solution of carboxylic acid (7.5 mg, 0.05 mmol) in CH_2Cl_2 (1 mL), HATU (23 mg, 0.06 mmol), DIPEA (10.4 µL, 0.06 mmol) and amine **1e** (26 mg, 0.05 mmol) were added at 25 °C. The reaction mixture was stirred at room temperature for 1 h. After completion of the reaction (as monitored by LC-MS), the reaction mixture was quenched with H_2O (2 mL). The aqueous layer was extracted with CH_2Cl_2 (3 × 3 mL) evaporated under *vacuo*. The crude product was purified by

preparative HPLC using water:ACN (0.1% TFA) solvent gradient to afford **1k** as colorless solid in 19.64 mg (yield = 61 %). *Compound **1k** formed an adduct with CD_3OD (40%) during the NMR acquisition.

^{1}H NMR (500 MHz, CD_3OD): δ 1.24-1.44 (m, 2H), 1.89 (br. s., 2H), 2.20 (br. s., 3H), 3.10 (br. s., 1H), 3.19 (br. s., 1H), 3.47 (br. s., 1H), 3.52 (br. s., 3H), 3.60-3.78 (m, 2H), 3.96 (d, *J* = 12.4 Hz, 1H), 4.07 (d, *J* = 11.0 Hz, 1H), 4.18 (d, *J* = 12.4 Hz, 1H), 5.35 (br. s., 1H), 5.41 (br. s., 1H), 6.04 (br. s., 1H), 6.25 (br. s., 1H), 7.11 (br. s., 2H), 7.19 (br. s., 3H), 7.34-7.52 (m, 4H), 7.72 (br. s., 2H), 7.86 (br. s., 2H), 8.40 (br. s., 1H); **^{13}C NMR** (126 MHz, CD_3OD): δ 18.4, 26.8, 39.3, 50.6, 51.3, 54.1, 59.7, 97.4, 102.5, 118.5, 119.3, 123.9, 125.9, 126.5, 126.8, 127.6, 128.1, 129.2, 129.7, 130.5, 134.2, 141.8, 151.9, 152.7, 156.4, 159.2, 167.9, 168.7, 192.0; **HR-MS** (m/z): Calculated for $C_{36}H_{37}N_8O_4$ $[M+H]^+$: 645.2938; Found $[M+H]^+$: 645.2941.

(3R,5R,7R)-N-(2-(2-(((R)-3-(4-amino-3-(4-phenoxyphenyl)-1H-pyrazolo[3,4-d]pyrimidin-1-yl)piperidin-1-yl)methyl)acrylamido)ethyl)adamantane-1-carboxamide (1l)

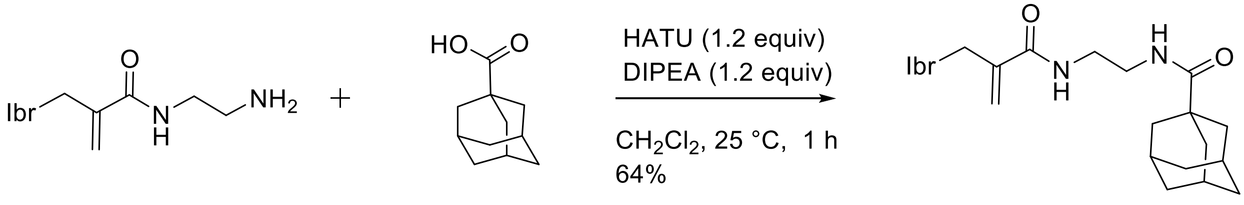

1e **1l**

To a stirred solution of adamantane carboxylic acid (9 mg, 0.05 mmol) in CH_2Cl_2 (1 mL), HATU (23 mg, 0.06 mmol), DIPEA (10.4 µL, 0.06 mmol) and amine **1c** (26 mg, 0.05 mmol) were added at 25 °C. The reaction mixture was stirred at room temperature for 1 h. After completion of the reaction (as monitored by LC-MS), the reaction mixture was quenched with H_2O (2 mL). The aqueous layer was extracted with CH_2Cl_2 (3 × 3 mL) evaporated under *vacuo*. The crude product was purified by preparative HPLC using water:ACN (0.1% TFA) solvent gradient to afford **1l** as colorless solid in 21.5 mg (yield = 64 %).

^{1}H NMR (400 MHz, CD_3OD) δ 1.31 (s, 3H), 1.57-1.69 (m, 3H), 1.72 (br. s., 2H), 1.77 (br. s., 7H), 1.89-2.00 (m, 3H), 2.16-2.25 (m, 2H), 2.28 (br. s., 1H), 3.08-3.21 (m, 1H), 3.38-3.46 (m, 2H), 3.70 (d, *J* = 10.1 Hz, 1H), 3.79 (d, *J* = 11.2 Hz, 1H), 3.96-4.13 (m, 2H), 4.18 (d, *J* = 13.0 Hz, 1H), 5.38-5.49 (m, 1H), 6.06 (br. s., 1H), 6.23 (s, 1H), 7.12 (d, *J* = 8.8 Hz, 1H), 7.16-7.28 (m, 2H), 7.39-7.49 (m, 2H), 7.69 (d, *J* = 8.6 Hz, 1H), 7.90 (d, *J* = 8.1 Hz, 1H), 8.37 (s, 1H); **^{13}C NMR** (126 MHz, CDCl3):

δ 28.0, 36.3, 39.0, 40.7, 41.5, 50.9, 97.2, 114.2, 116.5, 119.1, 120.0, 124.4, 124.7, 130.2, 146.6, 147.9, 151.8, 153.4, 155.5, 160.1, 160.8, 161.1, 181.0. **HR-MS** (m/z): Calculated for $C_{39}H_{47}N_8O_3$ $[M+H]^+$: 675.3771; Found $[M+H]^+$: 675.3775.

tert-butyl (R)-(15-((3-(4-amino-3-(4-phenoxyphenyl)-1H-pyrazolo[3,4-d]pyrimidin-1-yl)piperidin-1-yl)methyl)-9,14-dioxo-3,6-dioxa-10,13-diazahexadec-15-en-1-yl)carbamate (1m)

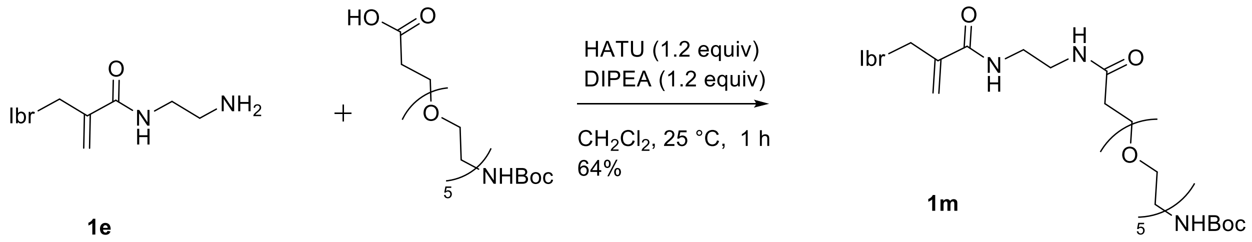

To a stirred solution of carboxylic acid (10.4 mg, 0.05mmol) in CH_2Cl_2 (1 mL), HATU (23 mg, 0.06 mmol), DIPEA (10.4 µL, 0.06 mmol) and amine **1e** (26 mg, 0.05 mmol) were added at 25 °C. The reaction mixture was stirred at room temperature for 1 h. After completion of the reaction (as monitored by LC-MS), the reaction mixture was quenched with H_2O (2 mL). The aqueous layer was extracted with CH_2Cl_2 (3 × 2 mL) evaporated under *vacuo* and the crude product was purified by preparative HPLC using water:ACN (0.1% TFA) solvent gradient to afford **1m** as colorless solid in 25.2 mg (yield = 64 %).

^{1}H NMR (500 MHz, CD_3OD): δ 1.44 (s, 11H), 1.99 (br. s., 1H), 2.12 (br. s., 1H), 2.24 (br. s., 2H), 2.39 (br. s., 2H), 3.19-3.23 (m, 3H), 3.49 (t, *J* = 5.6 Hz, 3H), 3.57-3.63 (m, 17H), 3.65-3.70 (m, 4H), 3.82 (br. s., 1H), 4.04 (br. s., 1H), 4.07-4.14 (m, 1H), 4.18 (d, *J* = 12.9 Hz, 1H), 5.48 (br. s., 1H), 6.04 (br. s., 1H), 6.25 (s, 1H), 7.14 (d, *J* = 8.0 Hz, 2H), 7.18-7.25 (m, 3H), 7.45 (t, *J* = 7.8 Hz, 2H), 7.92 (br. s., 1H), 8.42 (s, 1H). **^{13}C NMR** (126 MHz, CD_3OD): δ 20.1, 28.4, 28.9, 37.8, 39.6, 41.1, 41.4, 52.0, 53.2, 55.7, 61.3, 68.3, 71.2, 71.3, 71.4, 71.6, 71.6, 71.7, 71.7, 80.2, 99.0, 120.1, 120.9, 125.5, 127.6, 129.6, 131.3, 132.1, 133.9, 153.9, 154.5, 158.0, 158.6, 160.7, 169.1, 174.6; **HR-MS** (m/z): Calculated for $C_{41}H_{57}N_8O_8$ $[M+H]^+$: 789.4299; Found $[M+H]^+$: 789.4295.

4-((14-amino-3,6,9,12-tetraoxatetradecyl)amino)-2-(2,6-dioxopiperidin-3-yl)isoindoline-1,3-dione (pre-1n):

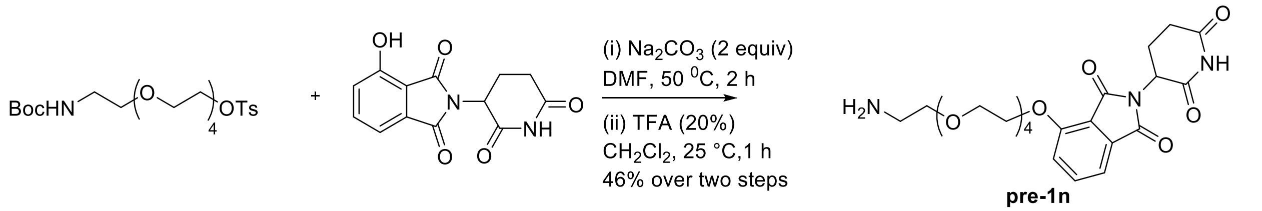

pre-1n

To a stirred solution of hydroxyl thalidomide (54.8 mg, 0.2 mmol) in dry DMF (1 mL), Na_2CO_3 (42 mg, 0.4 mmol) and tosyl compound (78 mg, 0.2 mmol) were added at 25 °C. The reaction mixture was stirred at 50 °C for 3 h. After completion of the reaction (as monitored by LC-MS), water was added. The aqueous layer was extracted with EtOAc (3 × 3 mL). The combined organic layers were concentrated *in vacuo* and the crude product was dissolved in 20% TFA in CH_2Cl_2 and stirred for another 1 h at room temperature. After completion of the reaction (as monitored by LC-MS), the reaction mixture was concentrated *in vacuo* and the crude product was purified by HPLC using water:ACN (0.1% formic acid) solvent gradient to afford thalidomide amine as white solid in 45.3 mg (46 % yield).

ESI-MS (m/z): calculated for $C_{23}H_{32}N_3O_9$ [M+H]+: 494.21; found: [M+H]+:494.54.

2-(((R)-3-(4-amino-3-(4-phenoxyphenyl)-1H-pyrazolo[3,4-d]pyrimidin-1-yl)piperidin-1-yl)methyl)-N-(14-((2-(2,6-dioxopiperidin-3-yl)-1,3-dioxoisoindolin-4-yl)amino)-3,6,9,12-tetraoxatetradecyl)acrylamide (1n)

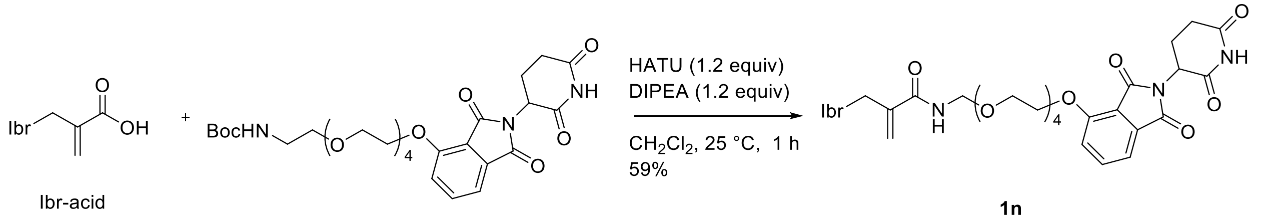

1n

To a stirred solution of carboxylic acid (23.5 mg, 0.05 mmol) in CH_2Cl_2 (1 mL), HATU (23 mg, 0.06 mmol), DIPEA (10.4 µL, 0.06 mmol) and amine **pre-1n** (24.5 mg, 0.05 mmol) were added at 25 °C. The reaction mixture was stirred at room temperature for 1 h. After completion of the reaction (as monitored by LC-MS), the reaction mixture was quenched with H_2O (2 mL) at 0 °C. The aqueous layer was extracted with CH_2Cl_2 (3 × 4 mL) evaporated under *vacuo*. The crude product was purified by preparative HPLC using water:ACN (0.1% TFA) solvent gradient to afford methacrylamide (**1n**) as colorless solid in 15.1 mg (yield = 59 %).

^{1}**H NMR** (500 MHz, CD_3OD): δ 1.97 (d, *J* = 15.3 Hz, 1H), 2.06-2.15 (m, 2H), 2.23 (br. s., 2H), 2.64-2.77 (m, 2H), 2.80-2.90 (m, 1H), 3.16 (br. s., 1H), 3.39 (br. s., 2H), 3.44-3.66 (m, 15 H), 3.67-3.82 (m, 4H), 3.89 (br. s., 2H), 4.04 (br. s., 2H), 4.16 (d, *J* = 12.9 Hz, 1H), 4.35 (br. s., 2H), 5.08 (dd,

J = 12.7, 5.4 Hz, 1H), 5.49 (br. s., 1H), 6.03 (s, 1H), 6.27 (s, 1H), 7.12 (d, *J* = 8.0 Hz, 2H), 7.18 (d, *J* = 8.4 Hz, 2H), 7.21 (t, *J* = 7.4 Hz, 1H), 7.39-7.52 (m, 4H), 7.75 (t, *J* = 7.9 Hz, 1H), 7.91 (br. s., 2H), 8.45 (br. s., 1 H); **^{13}C NMR** (126 MHz, CD_3OD): δ 18.5, 22.2, 26.7, 30.8, 39.2, 42.5, 49.0, 50.4, 51.7, 54.0, 60.0, 68.8, 69.0, 69.1, 69.8, 70.1, 70.1, 70.5, 97.1, 115.3, 116.8, 118.5, 119.4, 119.5, 124.0, 125.4, 128.0, 129.8, 130.5, 132.0, 133.7, 136.6, 147.1, 149.6, 152.2, 156.3, 159.3, 160.6, 165.9, 167.1, 167.5, 170.1, 173.1. **HR-MS** (m/z): Calculated for $C_{49}H_{56}N_9O_{11}$ $[M+H]^+$: 946.4099; Found $[M+H]^+$: 946.4092.

3-(2-(2-aminoethoxy)ethoxy)-N-(2-(2,6-dioxopiperidin-3-yl)-1-oxoisoindolin-4-yl)propanamide (pre-1o):

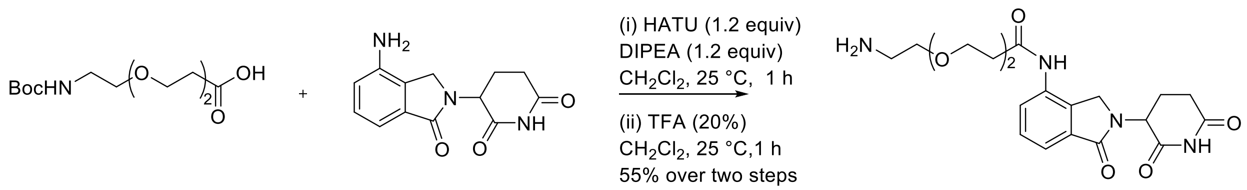

To a stirred solution of carboxylic acid (27.7 mg, 0.1 mmol) in CH_2Cl_2 (1 mL), HATU (46 mg, 0.12 mmol), DIPEA (20.9 µL, 0.12 mmol) and lenalidomide (26 mg, 0.1 mmol) were added at 25 °C. The reaction mixture was stirred at room temperature for 1 h. After completion of the reaction (as monitored by LC-MS), water was added and the aqueous layer was extracted with CH_2Cl_2 (3 × 3 mL). The combined organic layer was concentrated *in vacuo* and the crude product was dissolved 20%TFA in DCM and allowed to stir at room temperature for 1 h. After completion, the reaction mixture was evaporated under vacuo and the crude product was purified by HPLC using water:ACN (0.1% formic acid) solvent gradient to afford thalidomide amine (**pre-1o**) as white solid in 23 mg (55% yield).

ESI-MS (m/z): calculated for $C_{19}H_{25}N_4O_6$ [M+H]+: 405.13; found: [M+H]+:405.65.

3-(2-(2-((R)-3-(4-amino-3-(4-phenoxyphenyl)-1H-pyrazolo[3,4-d]pyrimidin-1-yl)piperidin-1-yl)ethoxy)ethoxy)-N-(2-(2,6-dioxopiperidin-3-yl)-1-oxoisoindolin-4-yl)propanamide (1o)

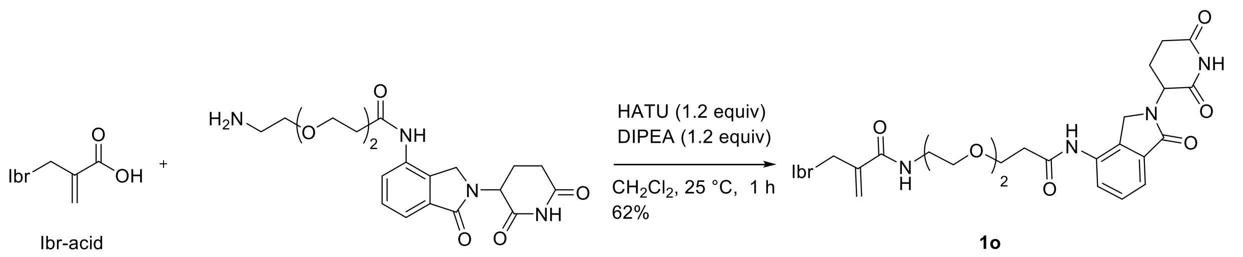

To a stirred solution of carboxylic acid (23.5 mg, 0.05 mmol) in CH_2Cl_2 (1 mL), HATU (23 mg, 0.06 mmol), DIPEA (10.4 µL, 0.06 mmol) and amine **pre-1o** (21 mg, 0.1 mmol) were added at 25 °C. The reaction mixture was stirred at room temperature for 1 h. After completion of the reaction (as monitored by LC-MS), water was added and the aqueous layer was extracted with CH_2Cl_2 (3 × 3 mL). The organic layer was concentrated *in vacuo* and the crude product was purified by HPLC using water:ACN (0.1% formic acid) solvent gradient to afford white solid **1o** in 26.9 mg (62% yield).

^{1}H NMR (400 MHz, CD_3OD δ 1.39 (s, 2H), 2.02 (br. s., 2H), 2.17-2.38 (m, 4H), 2.53 (qd, *J* = 13.2, 4.7 Hz, 1H), 2.74 (t, *J* = 5.1 Hz, 2H), 2.81-2.92 (m, 1H), 2.93-3.04 (m, 1H), 3.13-3.26 (m, 1H), 3.53-3.63 (m, 3H), 3.63-3.77 (m, 6H), 3.81 (br. s., 1H), 3.89 (t, *J* = 5.7 Hz, 2H), 4.01 (d, *J* = 12.8 Hz, 1H), 4.09-4.21 (m, 2H), 4.55 (s, 2H), 5.24 (dd, *J* = 13.3, 5.2 Hz, 1H), 5.48 (br. s., 1H), 5.98 (d, *J* = 5.9 Hz, 1H), 6.22-6.28 (m, 1H), 7.20 (d, *J* = 7.7 Hz, 2H), 7.23-7.33 (m, 3H), 7.47-7.61 (m, 3H), 7.69 (d, *J* = 5.9 Hz, 1H), 7.78 (dd, *J* = 7.5, 4.8 Hz, 1H), 7.88-8.00 (m, 2H), 8.46 (s, 1 H); **^{13}C NMR** (101 MHz, CD3OD δ 18.5, 22.8, 26.9, 29.4, 30.9, 36.4, 39.1, 50.6, 51.6, 52.2, 54.0, 59.7, 66.6, 68.9, 69.8, 69.9, 97.5, 118.5, 119.3, 120.1, 123.9, 126.4, 127.9, 128.7, 129.8, 130.4, 132.5, 133.2, 135.0, 156.4, 159.0, 167.3, 169.6, 170.9, 173.2; **HR-MS** (m/z): Calculated for $C_{42}H_{46}N_9O_7$ $[M+H]^+$: 788.3520; Found $[M+H]^+$: 788.3524.

1-amino-N-(2-(2,6-dioxopiperidin-3-yl)-1-oxoisoindolin-4-yl)-3,6,9,12-tetraoxapentadecan-15-amide (pre-1p)

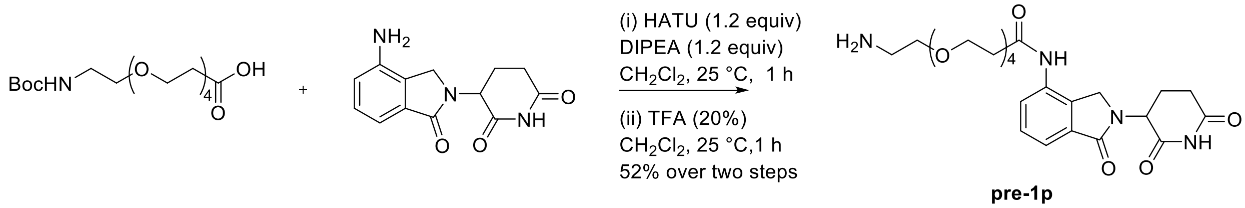

To a stirred solution of carboxylic acid (36.5 mg, 0.1 mmol) in CH_2Cl_2 (1 mL), HATU (46 mg, 0.12 mmol), DIPEA (21 µL, 0.12 mmol) and lenalidomide (26 mg, 0.1 mmol) were added at 25 °C. The reaction mixture was stirred at room temperature for 1 h. After completion of the reaction (as monitored by LC-MS), water was added. The aqueous layer was extracted with CH_2Cl_2 (3 × 3 mL).

The organic layer was concentrated *in vacuo* and the crude product was dissolved in 20% TFA in DCM and allowed to stir at 25 °C for 2 h. The reaction mixture was concentrated and purified HPLC using water:ACN (0.1% formic acid) solvent gradient to afford **pre-1p** white solid in 26.3 mg (52% yield).

ESI-MS (m/z): calculated for $C_{24}H_{35}N_4O_8$ [M+H]+: 507.24; found: [M+H]+:507.34.

1-(2-(((R)-3-(4-amino-3-(4-phenoxyphenyl)-1H-pyrazolo[3,4-d]pyrimidin-1-yl)piperidin-1-yl)methyl)acrylamido)-N-(2-(2,6-dioxopiperidin-3-yl)-1-oxoisoindolin-4-yl)-3,6,9,12-tetraoxapentadecan-15-amide (1p)

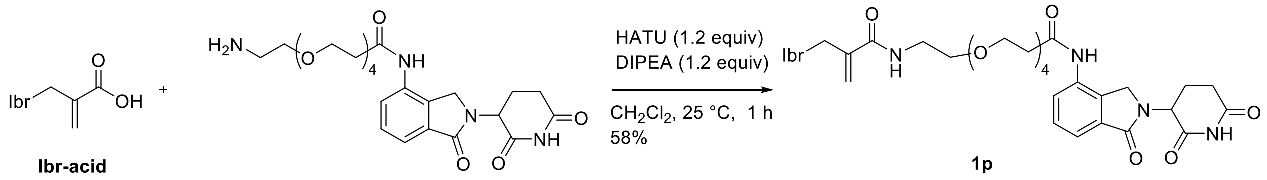

To a stirred solution of carboxylic acid (23.5 mg, 0.05 mmol) in CH_2Cl_2 (1 mL), HATU (23 mg, 0.06 mmol), DIPEA (10.4 µL, 0.06 mmol) and **pre 1p** amine (25 mg, 0.1 mmol) were added at 25 °C. The reaction mixture was stirred at room temperature for 1 h. After completion of the reaction (as monitored by LC-MS), water was added. the aqueous layer was extracted with CH_2Cl_2 (3 × 3 mL). The organic layer was concentrated *in vacuo* and the crude product was purified by HPLC using water:ACN (0.1% formic acid) solvent gradient to afford white solid **1p** in 27.84 mg (58% yield).

^{1}H NMR (500 MHz, CD_3OD δ 0.83-0.94 (m, 1H), 1.27-1.39 (m, 7H), 1.63 (br. s., 1H), 1.88-2.00 (m, 1H), 2.05 (br. s., 1H), 2.20 (br. s., 4H), 2.28 (br. s., 1H), 2.40-2.52 (m, 1H), 2.68 (br. s., 2H), 2.75-2.84 (m, 1H), 2.89 (d, J = 14.3 Hz, 1H), 3.13 (br. s., 1H), 3.18 (br. s., 1H), 3.50 (s, 6H), 3.47 (s, 5 H), 3.57 (br. s., 4H), 3.63 (br. s., 7H), 3.75 (br. s., 2H), 3.83 (br. s., 3H), 3.99 (br. s., 1H), 4.06 (br. s., 1H), 4.13 (d, J = 11.4 Hz, 1H), 4.49 (br. s., 2H), 5.12-5.24 (m, 1H), 5.42 (br. s., 1H), 6.00 (br. s., 1H), 6.24 (br. s., 1H), 7.13 (br. s., 2H), 7.15-7.26 (m, 4H), 7.44 (br. s., 2H), 7.50 (br. s., 1H), 7.62 (br. s., 1H), 7.73 (br. s., 2H), 7.88 (br. s., 2H), 8.39 (br. s., 1 H); **^{13}C NMR** (125 MHz, CD3OD δ 14.6, 20.1, 23.9, 24.3, 28.4, 30.9, 32.5, 38.1, 40.7, 52.1, 53.8, 55.6, 61.4, 68.2, 70.4, 71.3, 71.5, 71.6, 71.6, 120.0, 120.9, 121.6, 125.5, 127.9, 129.5, 130.2, 131.3, 132.0, 134.1, 134.7, 142.0, 143.0, 154.5, 157.3, 160.6, 168.9, 171.2, 174.8; **HR-MS** (m/z): Calculated for $C_{49}H_{57}N_{10}O_{10}$ [M+H]$^+$: 945.4259; Found [M+H]$^+$: 945.4256.

(R)-1-amino-N-(3-(3-(3-(4-amino-3-(4-phenoxyphenyl)-1H-pyrazolo[3,4-d]pyrimidin-1-yl)piperidin-1-yl)-3-oxopropyl)-14-(l1-oxidaneyl)-3l3,6,9,12-tetraoxatetradecyl)-3,6,9,12,15-pentaoxaoctadecan-18-amide (1r):

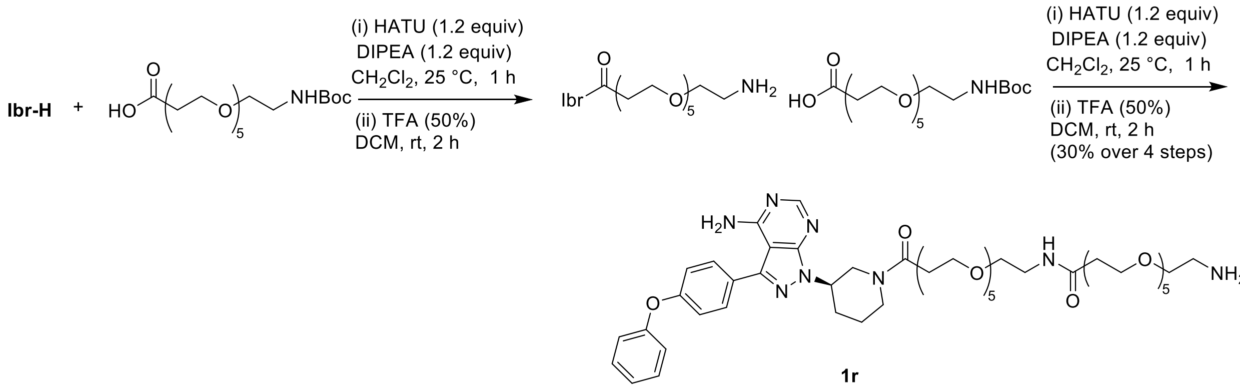

1r

To a stirred solution of carboxylic acid (40.9 mg, 0.1 mmol) in CH_2Cl_2 (5 mL), HATU (45.6 mg, 0.12 mmol), DIPEA (20.9 µL, 0.12 mmol) and ibr-H (38.7 mg, 0.1 mmol) were added at 25 °C. The reaction mixture was stirred at room temperature for 1 h. After completion of the reaction (as monitored by LC-MS), 3 mL water was added. The aqueous layer was extracted with CH_2Cl_2 (3 × 5 mL). The combined organic layers were concentrated *in vacuo* and the crude product was dissolved in 20% TFA in DCM and allowed to stir at 25 °C for 2 h. The reaction mixture was concentrated to give the crude amine which was used as such for the next reaction.

To a stirred solution of carboxylic acid (40.9 mg, 0.1 mmol) in CH_2Cl_2 (5 mL), HATU (45.6 mg, 0.12 mmol), DIPEA (20.9 µL, 0.12 mmol) and crude amine (0.1 mmol) (obtained in previous step) were added at 25 °C. The reaction mixture was stirred at room temperature for 1 h. After completion of the reaction (as monitored by LC-MS), 3 mL water was added. The aqueous layer was extracted with CH_2Cl_2 (3 × 5 mL). The combined organic layers were concentrated *in vacuo* and the crude product was dissolved in 20% TFA in DCM and allowed to stir at 25 °C for 2 h. The reaction mixture was concentrated and purified HPLC using water:ACN (0.1% formic acid) solvent gradient to afford white solid **1r** in 29 mg (30% yield).

ESI-MS (m/z): calculated for $C_{48}H_{73}N_8O_{13}$ [M+H]+: 969.53; found: [M+H]+:969.89.

2-((4-(((6-amino-5-(4-phenoxyphenyl)pyrimidin-4-yl)amino)methyl)piperidin-1-yl)methyl)-N-(but-3-yn-1-yl)acrylamide (2a)

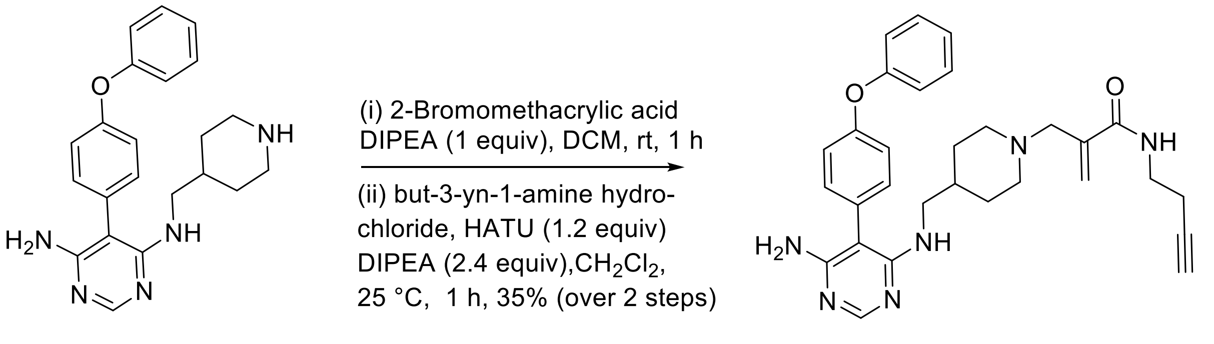

2a

To a stirred solution of evoburtinib amine (37.5 mg, 0.1 mmol) in anhydrous DCM (1 mL), DIPEA (17.8 μL, 0.1 mmol) and 2-(bromomethyl)acrylic acid (16.1 mg, 0.1 mmol) were added at 25 °C. The reaction mixture was stirred at room temperature for 1 h. After completion of the reaction (as monitored by LC-MS), 1 mL of H_2O was added and the aqueous layer was extracted with CH_2Cl_2 (3 × 2 mL). The combined organic layers was concentrated *in vacuo* and the crude product was used for the next reaction without further purification.

To a solution of crude evabrutinib carboxylic acid in CH_2Cl_2 (1 mL), HATU (46 mg, 0.12 mmol), DIPEA (41.8 μL, 0.24 mmol) and but-3-yn-1-aminehydrochloride (12.6 mg, 0.12 mmol) were added at 25 °C. The reaction mixture was stirred at room temperature for 1 h. After completion of the reaction (as monitored by LC-MS), water (2 mL) was added and the aqueous layer was extracted with CH_2Cl_2 (3 × 3 mL). The combined organic layer was concentrated *in vacuo* and the crude product was purified by preparative HPLC using water:ACN (0.1% formic acid) solvent gradient to afford white solid **2a** in 17.8 mg (35% yield).

^{1}H NMR (500 MHz, CD_3OD): δ 1.44-1.56 (m, 2H), 1.95 (d, *J* = 12.2 Hz, 3H), 2.33 (t, *J* = 2.6 Hz, 1H), 2.47 (td, *J* = 6.9, 2.6 Hz, 2H), 2.94 (t, *J* = 12.3 Hz, 2H), 3.43 (t, *J* = 6.9 Hz, 4H), 3.57 (d, *J* = 11.8 Hz, 2H), 3.97 (s, 2H), 6.01 (s, 1H), 6.25 (s, 1H), 7.14 (d, *J* = 8.7 Hz, 2H), 7.18-7.23 (m, 3H), 7.32 (d, *J* = 8.7 Hz, 2H), 7.43 (t, *J* = 8.0 Hz, 2H), 8.27 (s, 1H); **^{13}C NMR** (126 MHz, CD_3OD): δ 18.1, 27.0, 33.9, 38.3, 45.2, 52.4, 58.2, 69.5, 80.7, 95.6, 119.3, 119.8, 122.9, 123.8, 128.3, 129.7, 132.0, 133.3, 148.1, 156.4, 158.9, 167.5. **HR-MS** (m/z): Calculated for $C_{30}H_{35}N_6O_2$ $[M+H]^+$: 511.2821; Found $[M+H]^+$: 511.2816.

N-(but-3-yn-1-yl)-2-(((3S)-4-(6-fluoro-7-(2-fluoro-6-hydroxyphenyl)-1-(2-isopropyl-4-methylpyridin-3-yl)-2-oxo-1,2-dihydropyrido[2,3-d]pyrimidin-4-yl)-3-methylpiperazin-1-yl)methyl)acrylamide (3a)

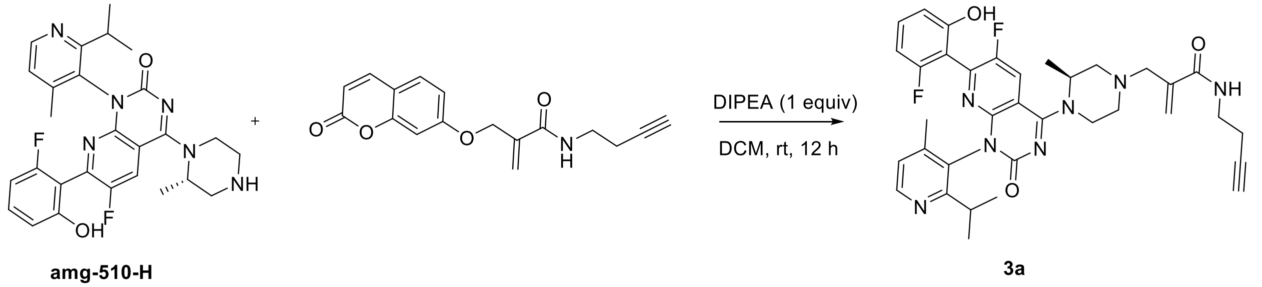

To a stirred solution of amg-510 amine (2.5 mg, 0.005 mmol) in anhydrous DCM (1 mL), DIPEA (1.73 µL, 0.01 mmol) and compound **3a**[2] (2.9 mg, 0.01 mmol) were added at 25 °C. The reaction mixture was stirred at room temperature for 12 h. After completion of the reaction (as monitored by LC-MS), CH_2Cl_2 was concentrated *in vacuo* to obtain the crude carboxylic acid. The crude product was purified by preparative HPLC using water:ACN (0.1% formic acid) solvent gradient to afford white solid **3a** in 1 mg (31.0% yield).

^{1}H NMR (400 MHz, CD_3COCD_3) δ 1.09-1.18 (m, 3H), 1.27 (d, *J* = 6.6 Hz, 3H), 1.64 (dd, *J* = 6.4, 4.2 Hz, 3H), 2.52-2.61 (m, 3H), 2.93-2.98 (m, 1H), 3.27 (d, *J* = 9.7 Hz, 1H), 3.49-3.67 (m, 4H), 3.88 (d, *J* = 12.8 Hz, 1H), 3.95-4.05 (m, 1H), 4.08-4.23 (m, 1H), 4.37 (dd, *J* = 13.1, 3.9 Hz, 1H), 4.54-4.68 (m, 2H), 6.09 (s, 1H), 6.37 (s, 1H), 6.78 (t, *J* = 9.0 Hz, 1H), 6.85 (d, *J* = 8.4 Hz, 1H), 7.34-7.45 (m, 2H), 8.50 (d, *J* = 9.0 Hz, 1H), 8.62 (d, *J* = 5.1 Hz, 1H). **^{13}C NMR** (101 MHz, CD_3COCD_3) δ 14.5, 18.0, 19.6, 22.0, 22.3, 31.0, 39.2, 47.7, 53.3, 55.0, 58.3, 71.4, 82.7, 106.7, 107.2, 107.4, 113.0, 124.1, 124.3, 124.8, 128.2, 132.4, 133.0, 133.2, 148.5, 148.7, 150.8, 154.8, 158.0, 162.9, 164.3, 164.8, 164.9, 167.6; **HR-MS** (m/z): Calculated for $C_{35}H_{38}F_2N_7O_3$ $[M+H]^+$: 642.3004; Found $[M+H]^+$: 642.3009.

2-(ethoxycarbonyl)allyl 3,4,5-trimethoxybenzoate (4a)

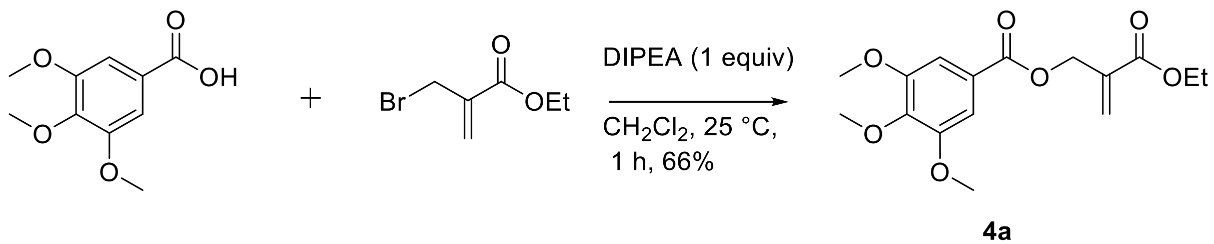

To a stirred solution of 3,4,5 trimethoxy benzoic acid (21.7 mg, 0.1 mmol) in anhydrous DCM (1 mL), DIPEA (17 µL, 0.1 mmol) and ethyl bromo methacrylate (14.6 µL, 0.1 mmol) were added at 25 °C. The reaction mixture was stirred at room temperature for 1 h. After completion of the reaction (as monitored by LC-MS), CH_2Cl_2 was concentrated *in vacuo* to obtain the crude carboxylic acid. The crude product was purified by HPLC using water:ACN (0.1% formic acid) solvent gradient to afford white solid **4a** in 10.7 mg (31.0% yield).

^{1}H NMR (500 MHz, $CDCl_3$) δ 1.31 (t, J = 7.1 Hz, 3H), 3.90 (s, 10 H), 4.26 (q, J = 7.2 Hz, 2H), 5.06 (s, 2H), 5.89 (s, 1H), 6.41 (s, 1H), 7.31 (s, 2 H); **^{13}C NMR** (126 MHz, $CDCl_3$) δ 14.1, 56.2, 60.9, 61.0, 62.9, 106.9, 124.8, 127.1, 135.6, 142.4, 152.9, 165.2, 165.5; **HR-MS** (m/z): Calculated for $C_{16}H_{20}NaO_7$ $[M+Na]^+$: 347.1107; Found $[M+Na]^+$: 347.1103.

References:

(1) Gabizon, R.; Shraga, A.; Gehrtz, P.; Livnah, E.; Shorer, Y.; Gurwicz, N.; Avram, L.; Unger, T.; Aharoni, H.; Albeck, S.; Brandis, A.; Shulman, Z.; Katz, B.-Z.; Herishanu, Y.; London, N. Efficient Targeted Degradation via Reversible and Irreversible Covalent PROTACs. *J. Am. Chem. Soc.* **2020**, *142 (*27), 11734–11742. https://doi.org/10.1021/jacs.9b13907.

(2) Pode, Z.; Peri-Naor, R.; Georgeson, J. M.; Ilani, T.; Kiss, V.; Unger, T.; Markus, B.; Barr, H. M.; Motiei, L.; Margulies, D. Protein Recognition by a Pattern-Generating Fluorescent Molecular Probe. *Nat. Nanotechnol.* **2017**, *12* (12), 1161–1168.

(3) Reddi, R. N.; Resnick, E.; Rogel, A.; Rao, B. V.; Gabizon, R.; Goldenberg, K.; Gurwicz, N.; Zaidman, D.; Plotnikov, A.; Barr, H.; Shulman, Z.; London, N. Tunable Methacrylamides for Covalent Ligand Directed Release Chemistry. *J. Am. Chem. Soc.* **2021**, *143* (13), 4979–4992.

NMR Spectra

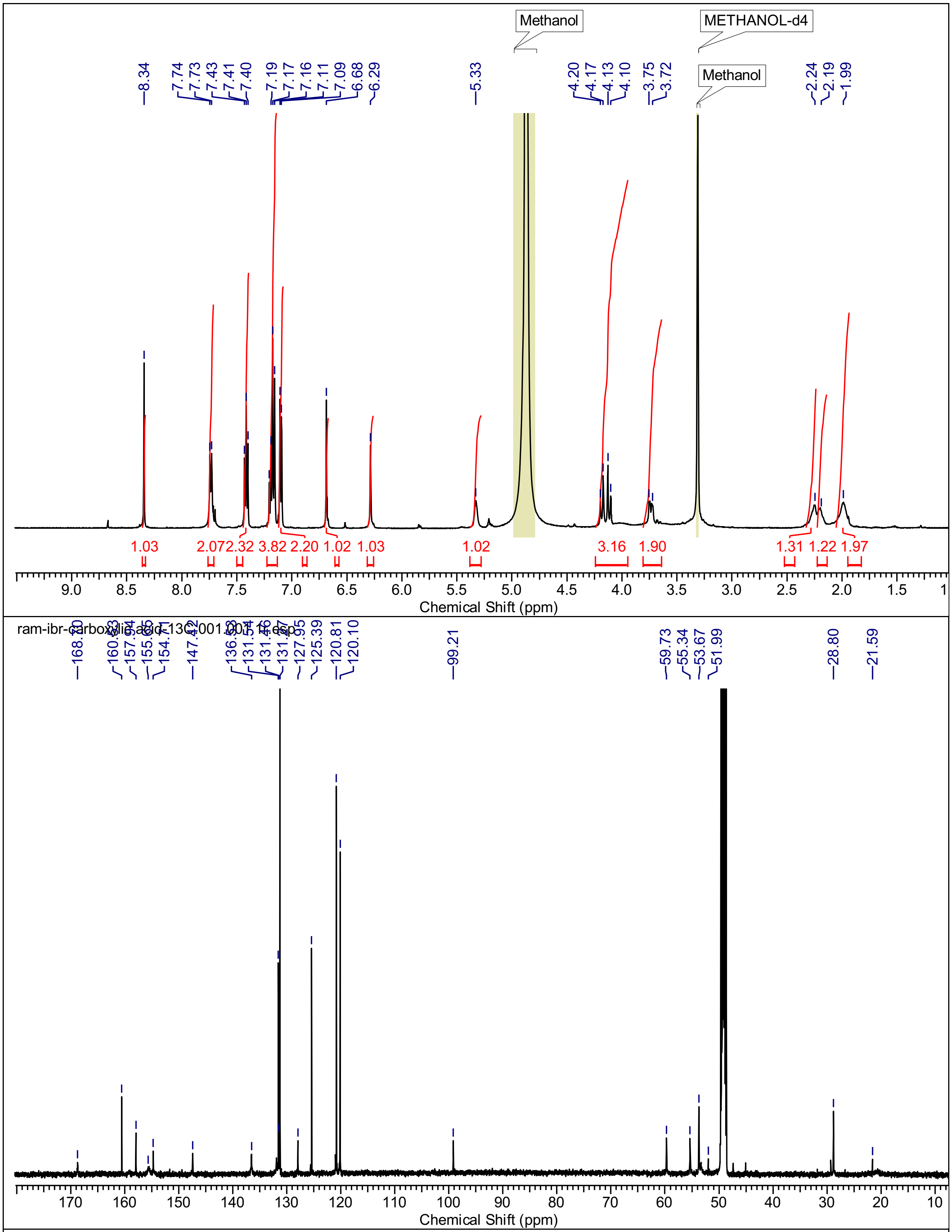

^{1}H and ^{13}C NMR spectra of Ibr-carboxylic acid

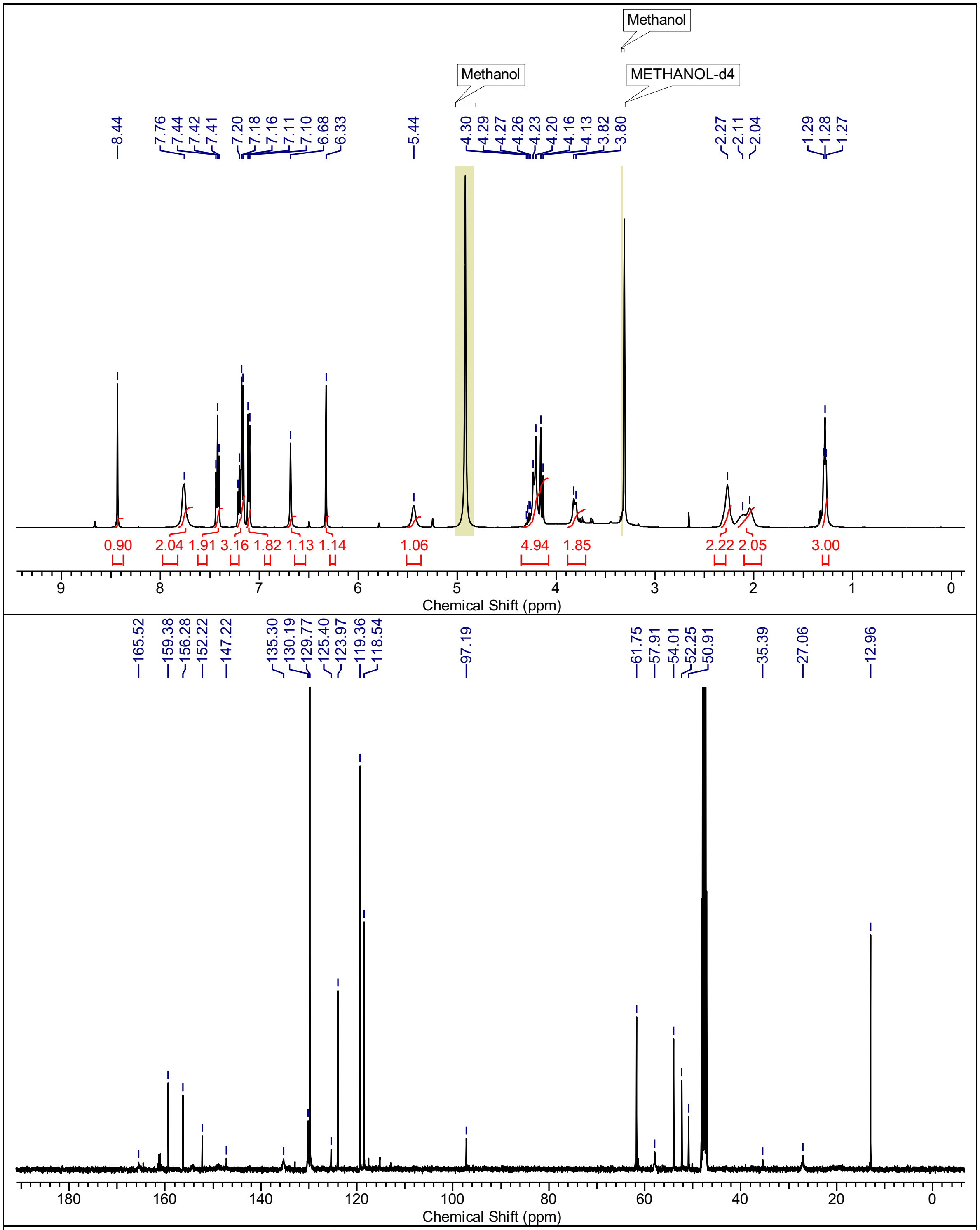

^{1}H and ^{13}C NMR spectra of **1a**

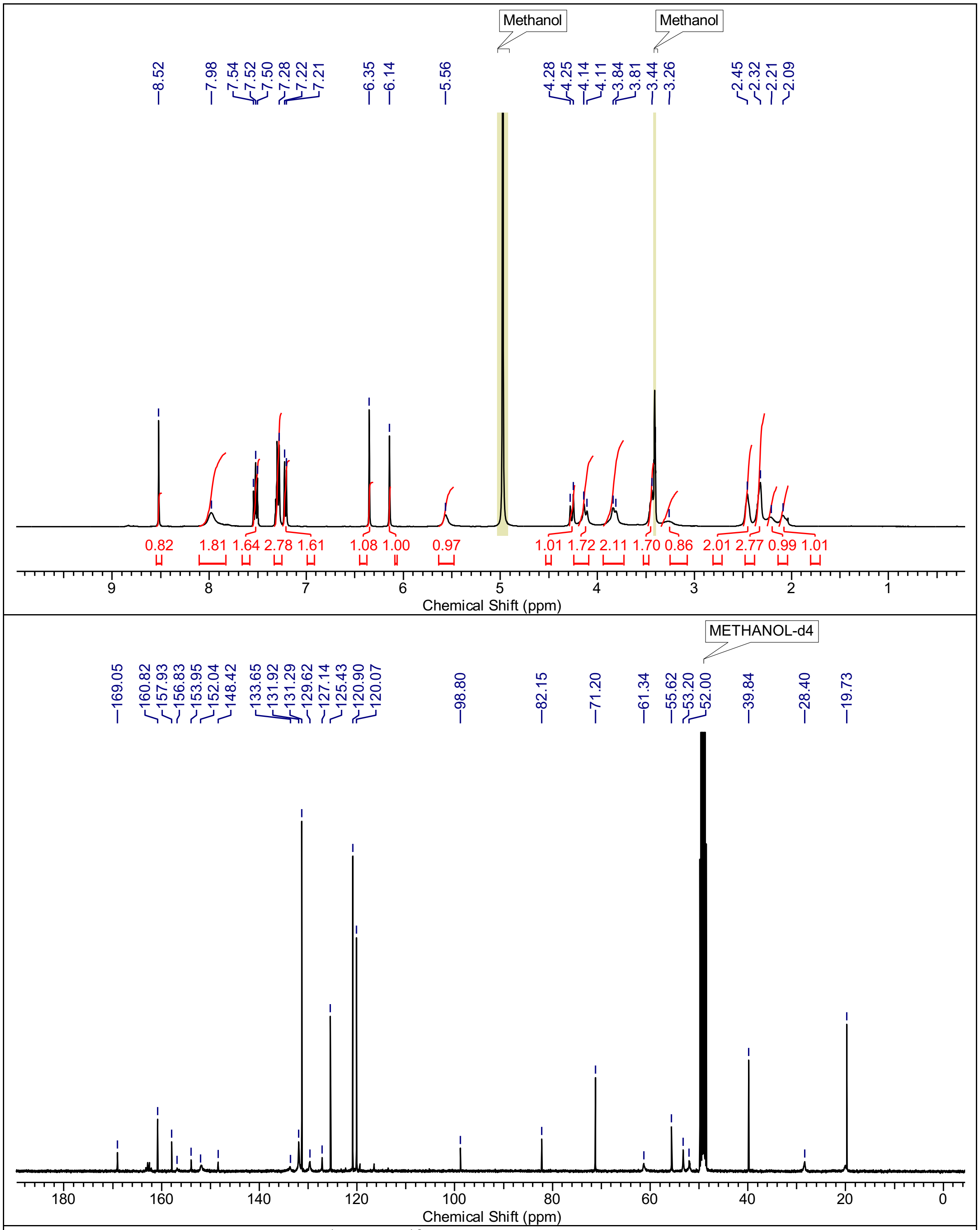

^{1}H and ^{13}C NMR spectra of **1b**

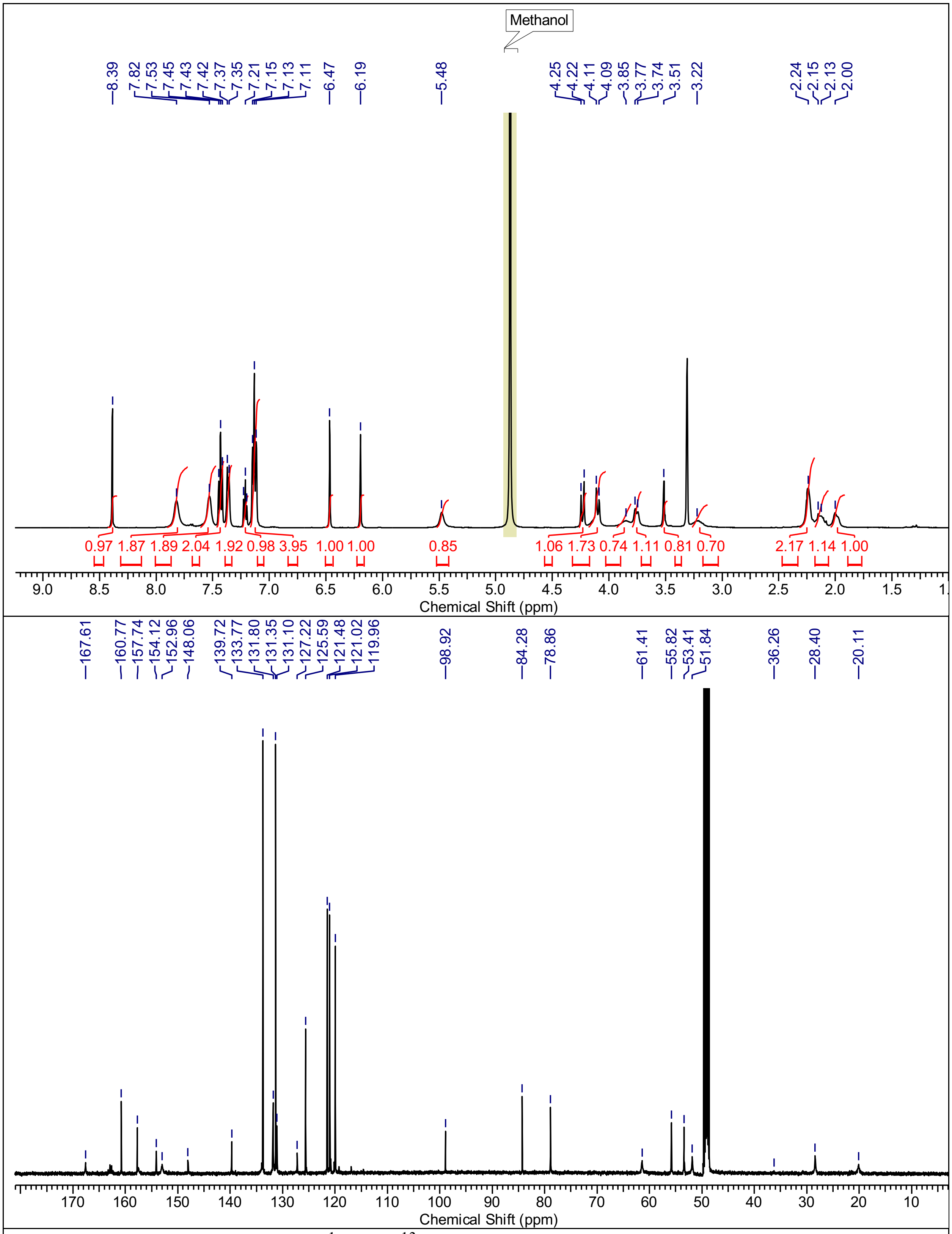

^{1}H and ^{13}C NMR spectra of **1c**

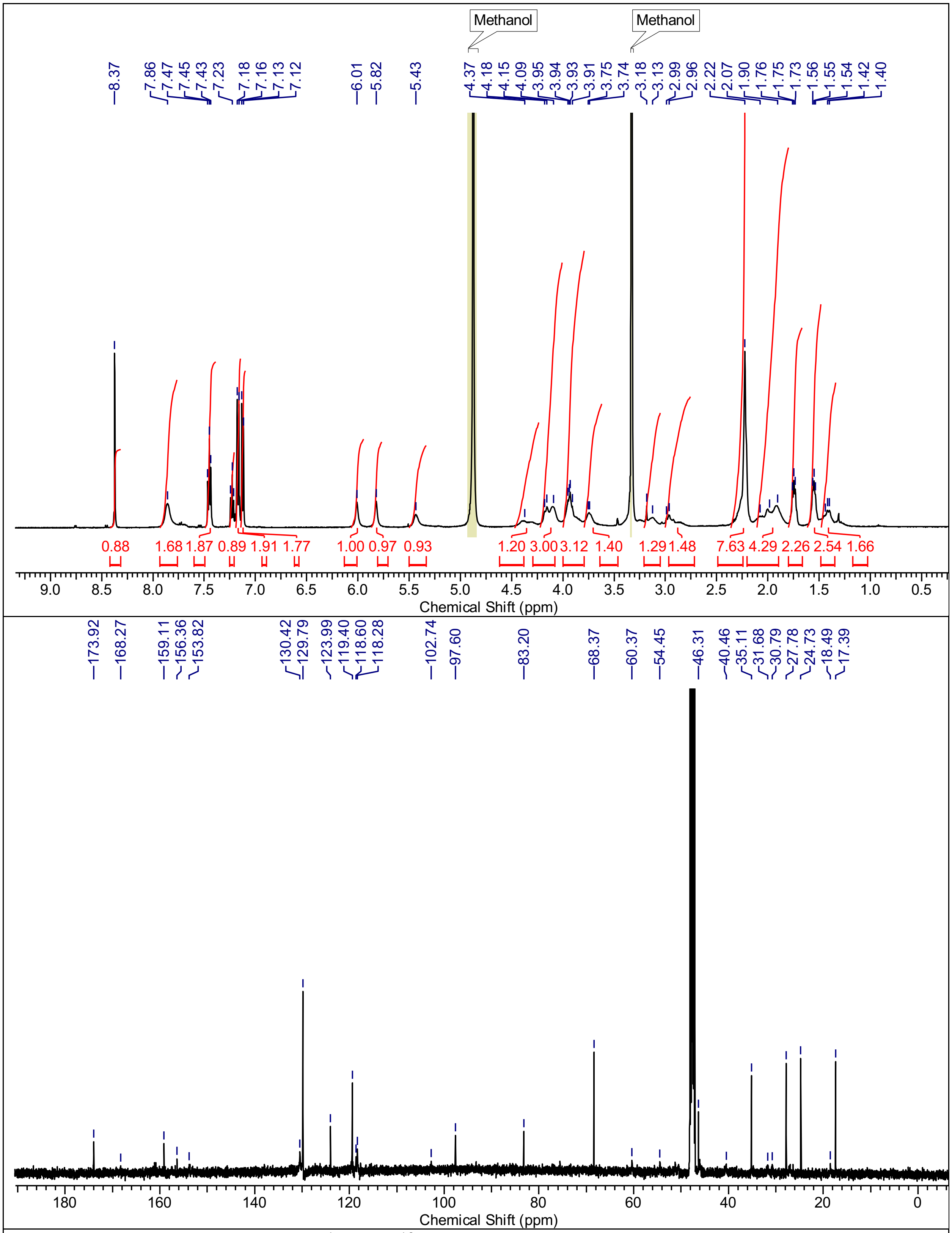

^{1}H and ^{13}C NMR spectra of **1d**

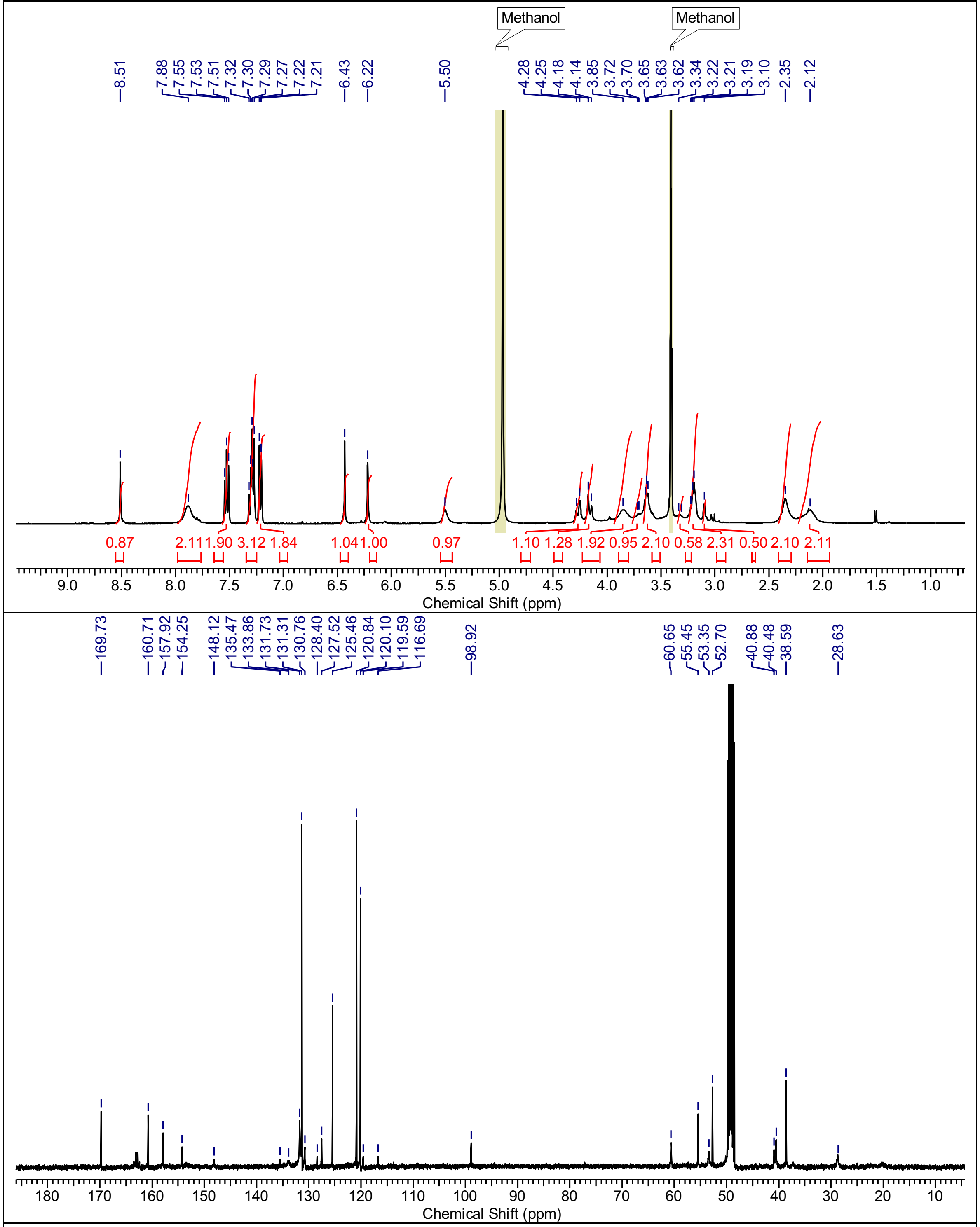

^{1}H and ^{13}C NMR spectra of **1e**

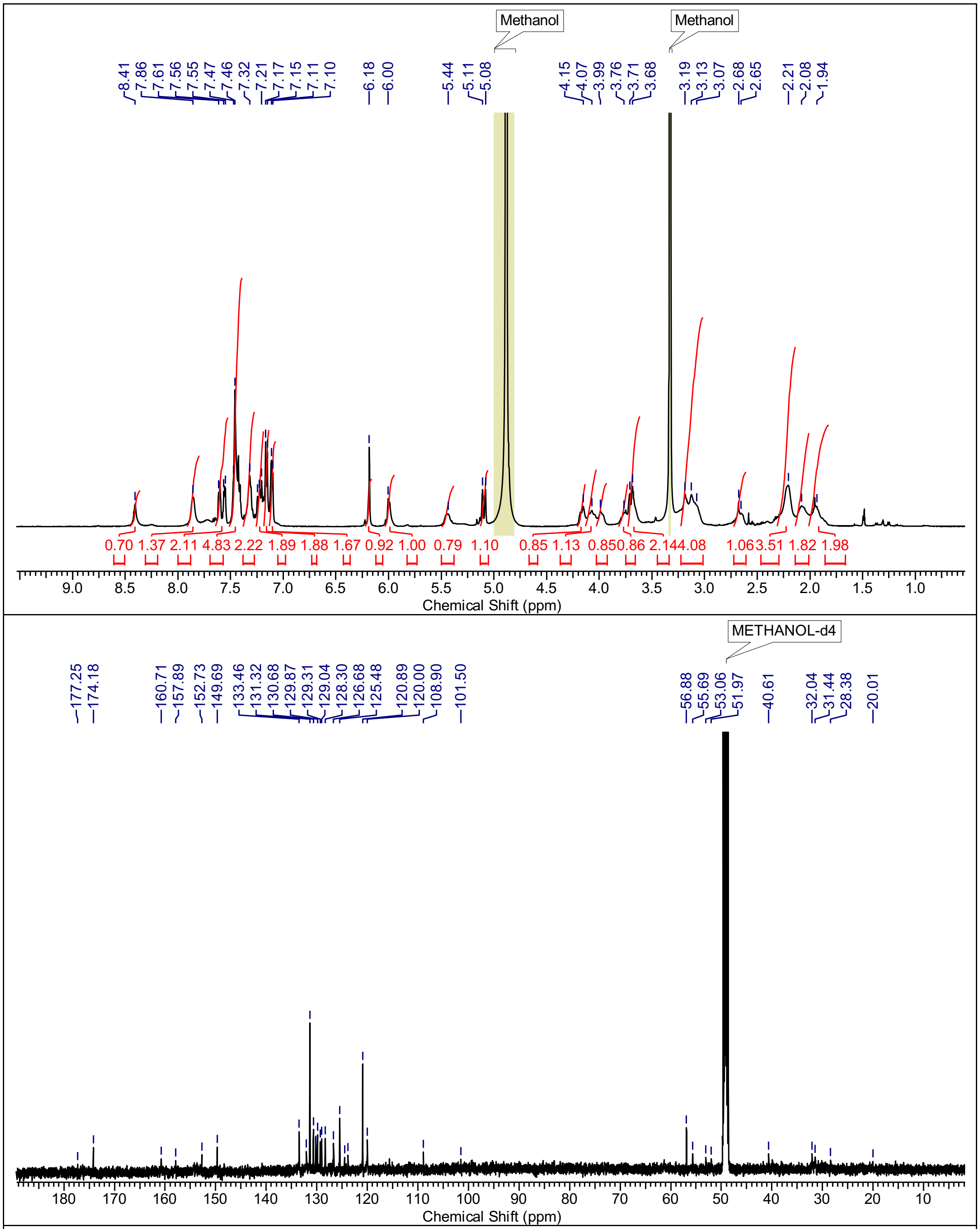

^{1}H and ^{13}C NMR spectra of **1f**

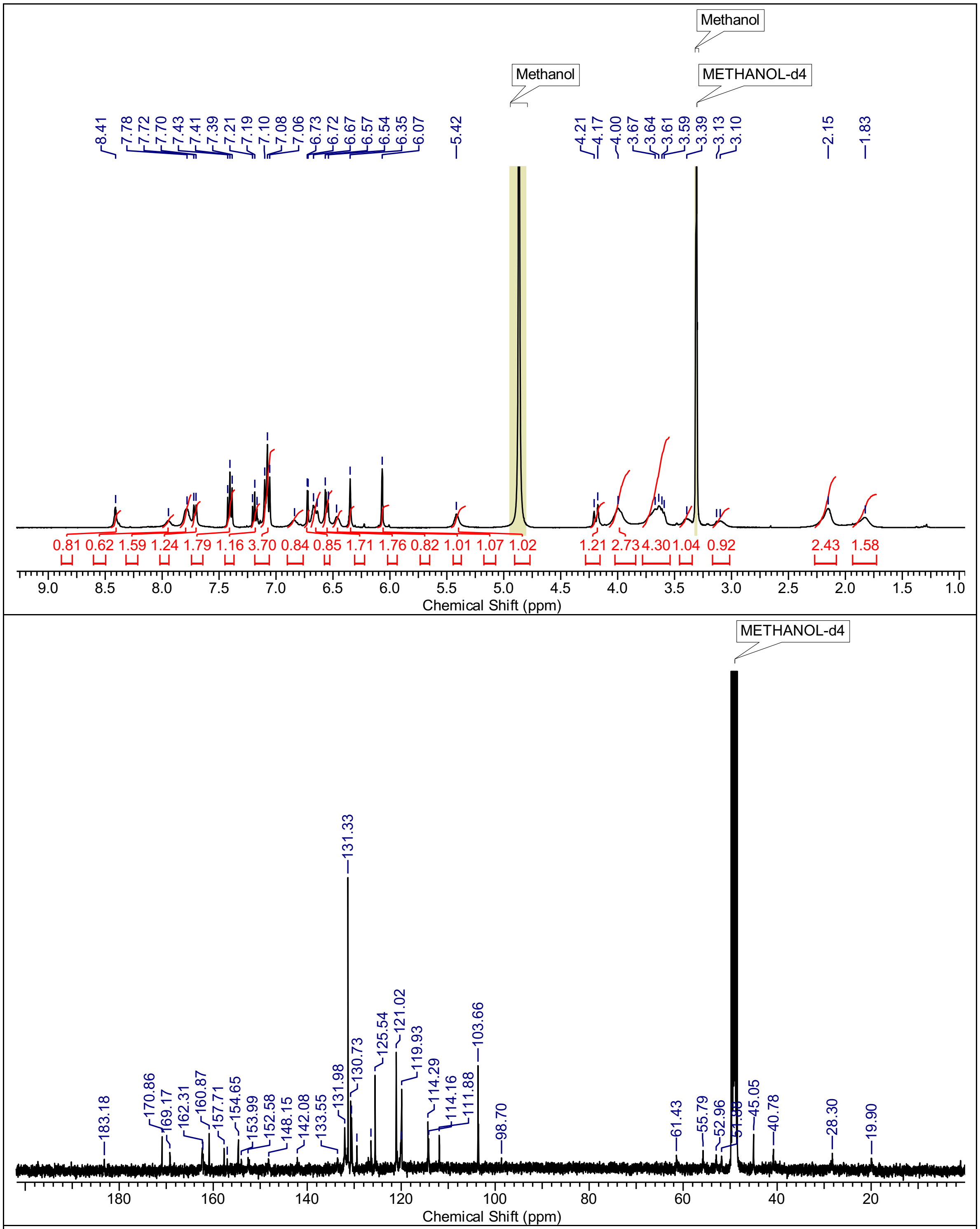

^{1}H and ^{13}C NMR spectra of **1g**

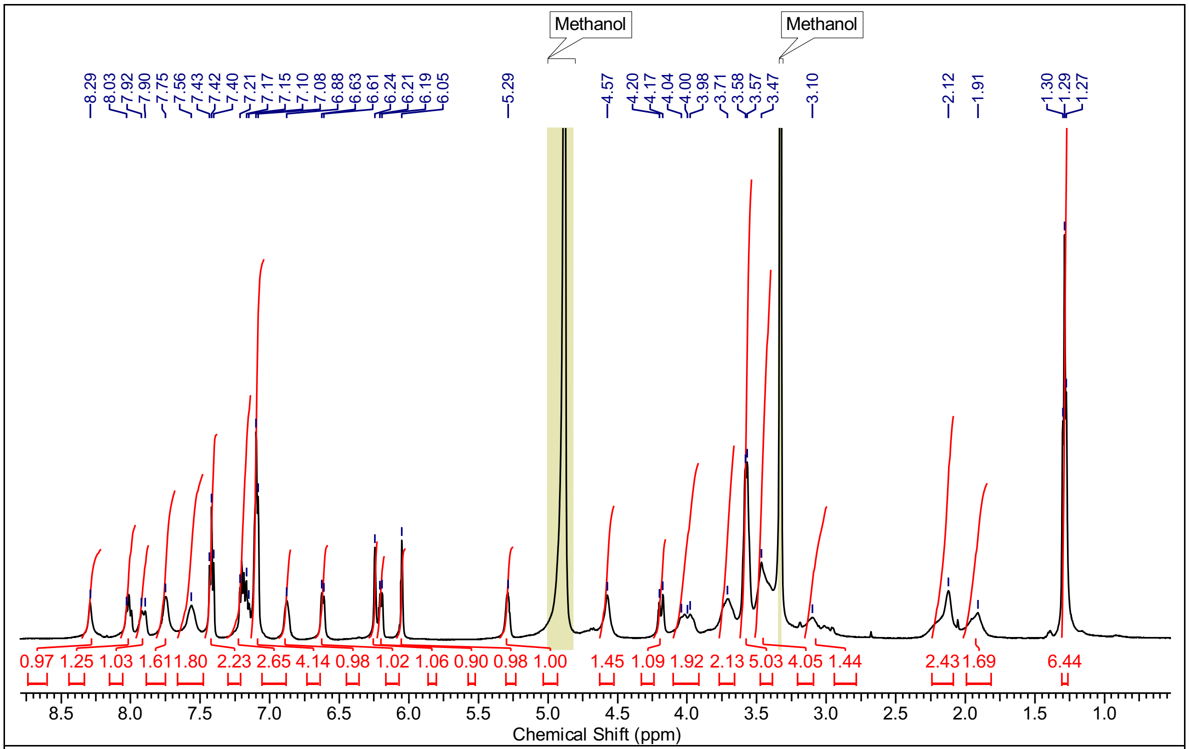

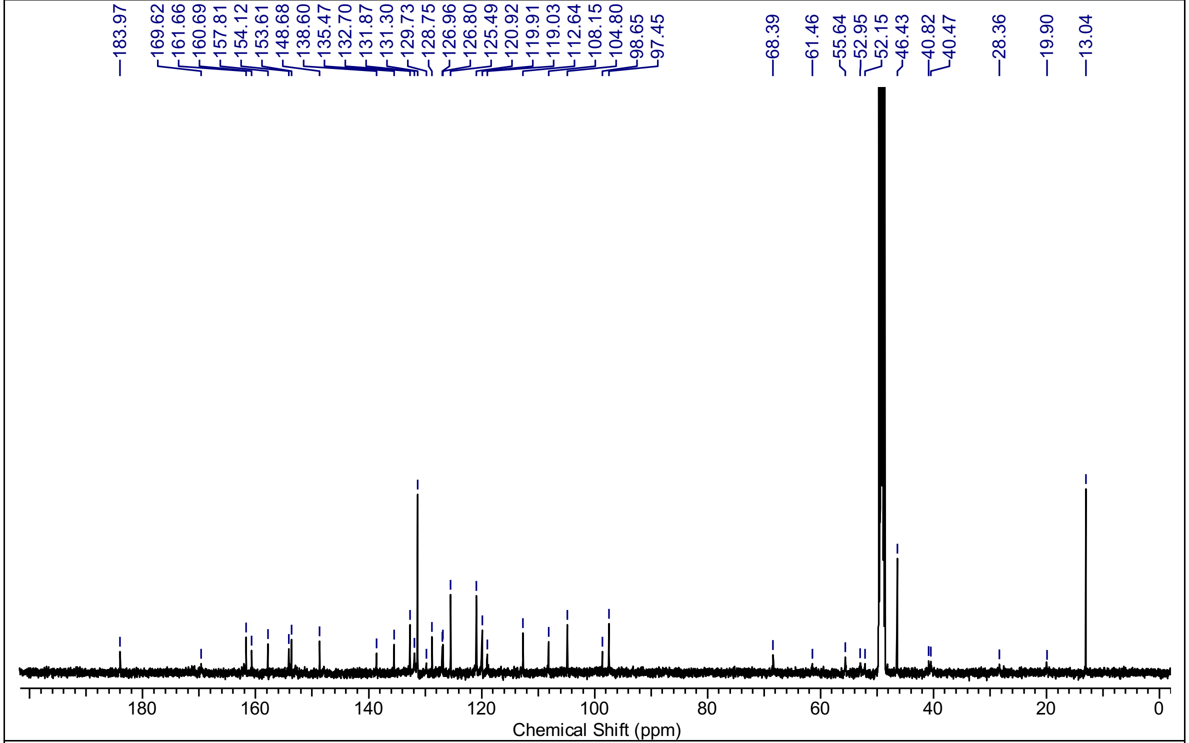

^{1}H and ^{13}C NMR spectra of **1h**

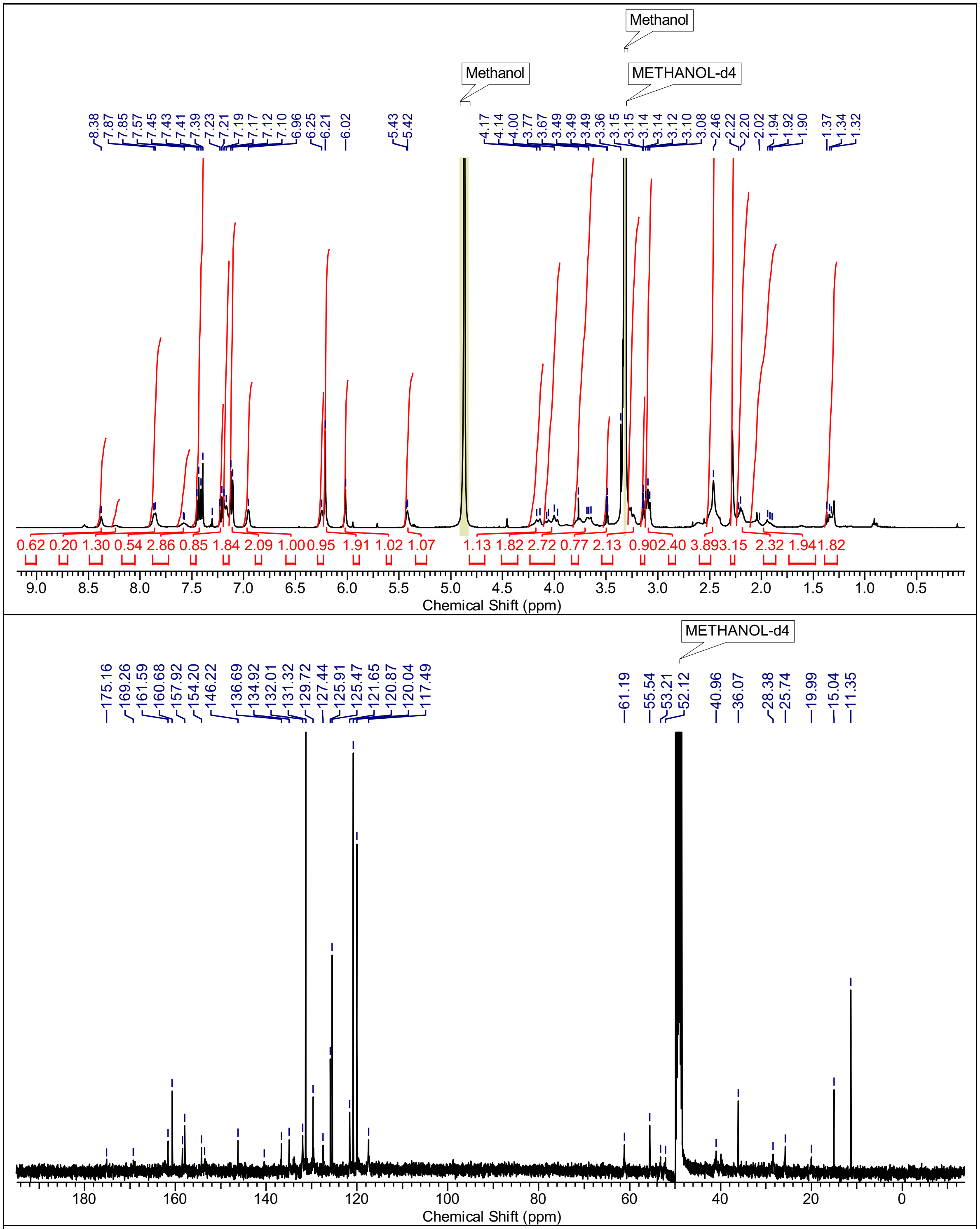

^{1}H and ^{13}C NMR spectra of **1i**

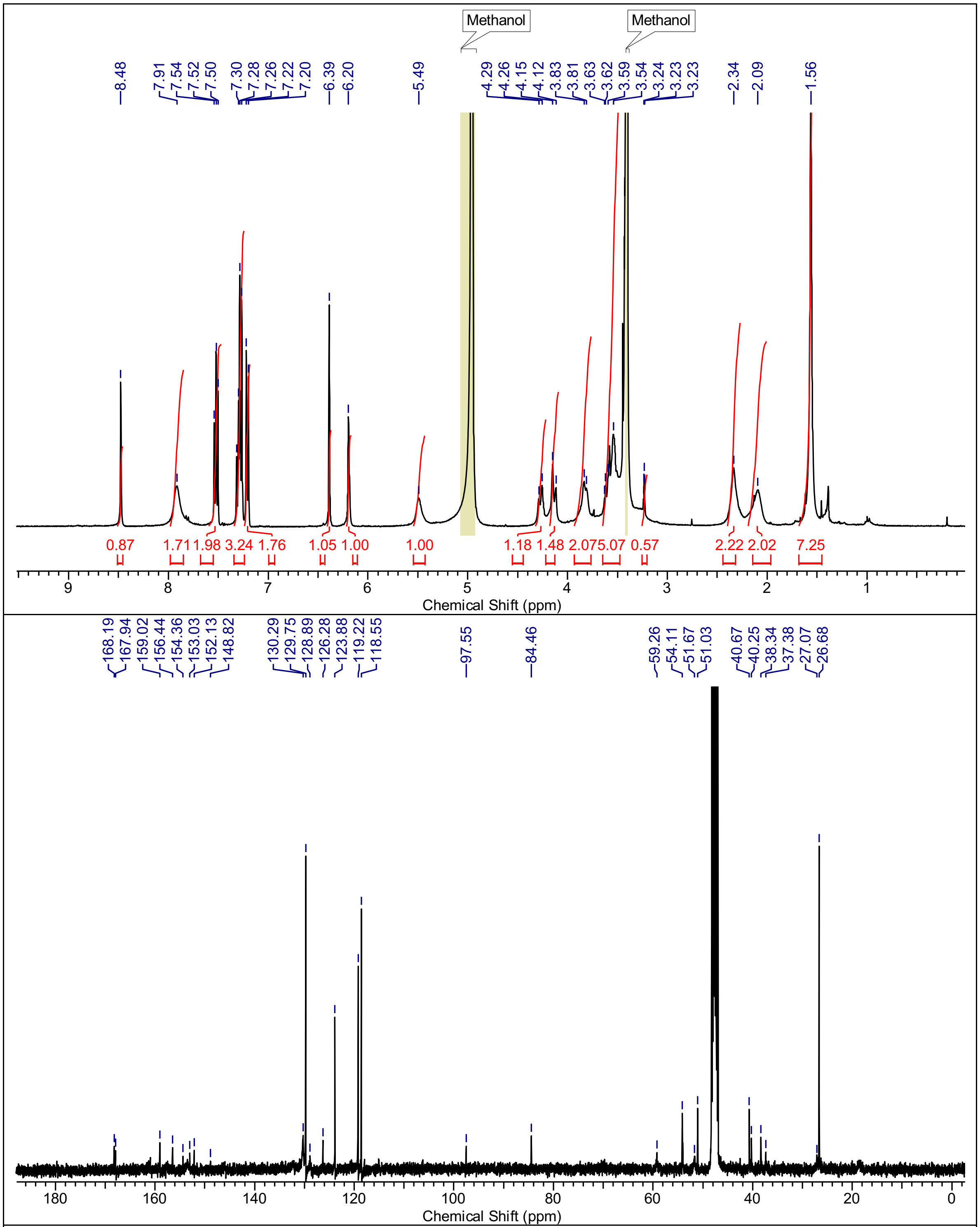

^{1}H and ^{13}C NMR spectra of **1j**

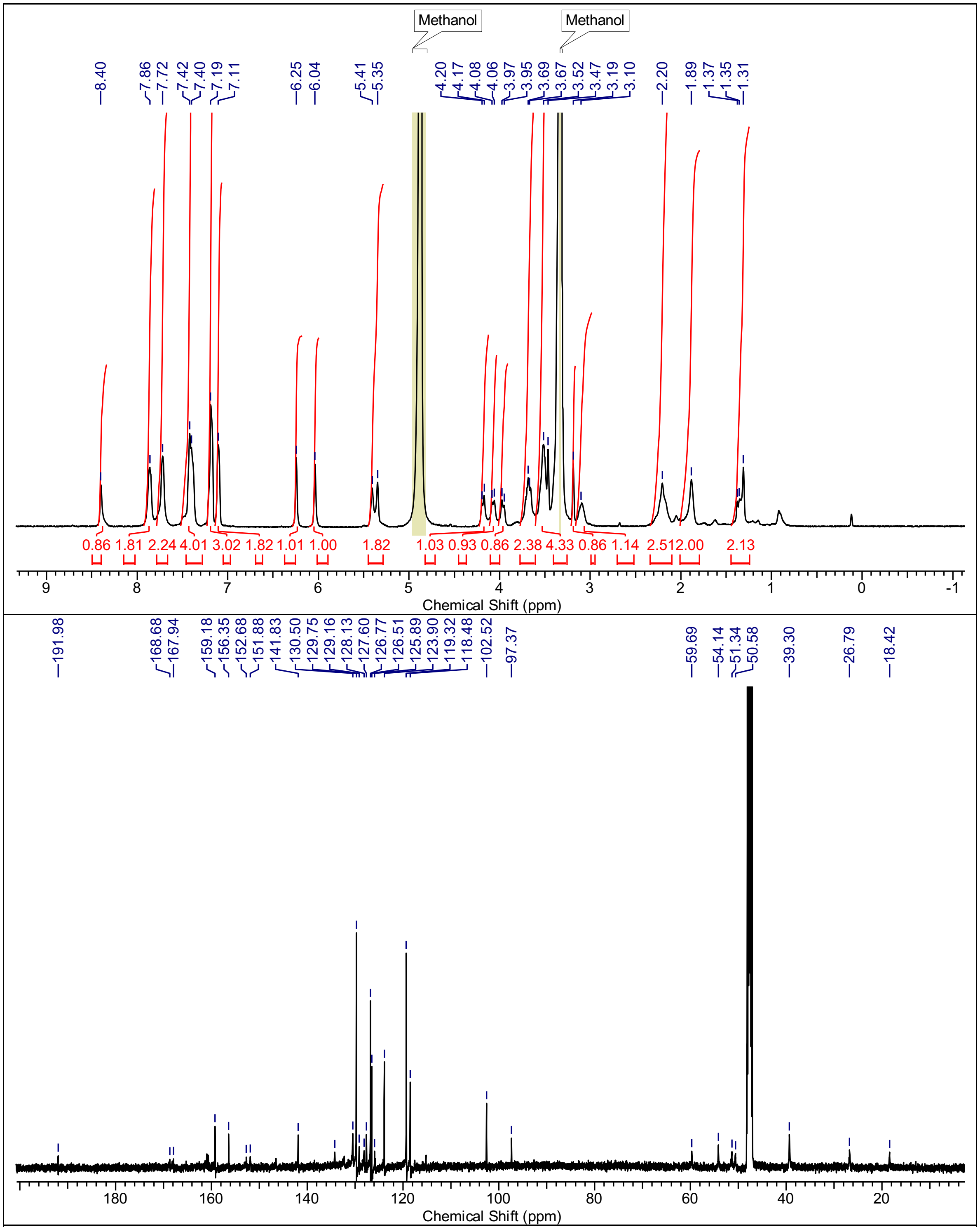

^{1}H and ^{13}C NMR spectra of **1k**

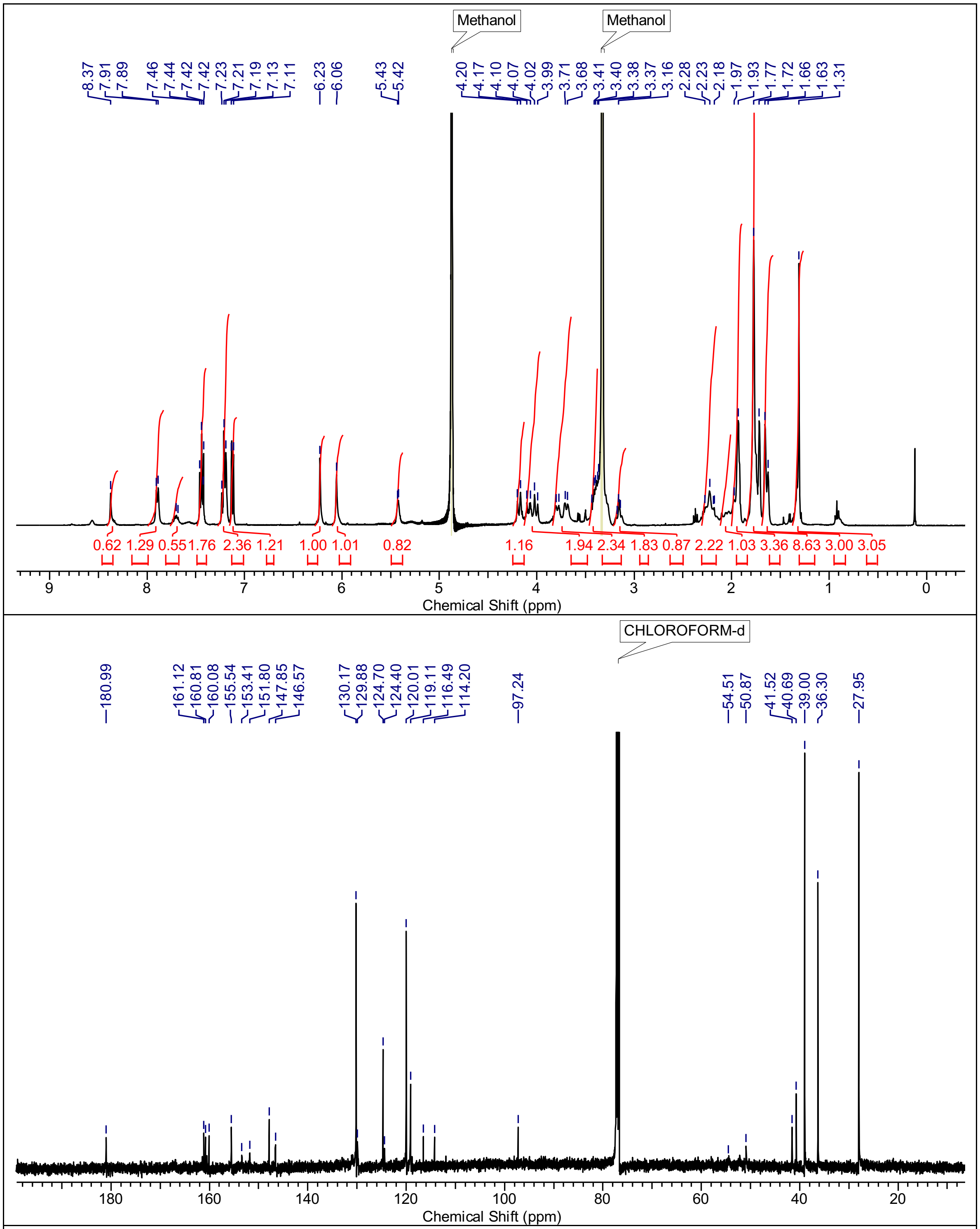

^{1}H and ^{13}C NMR spectra of **1l**

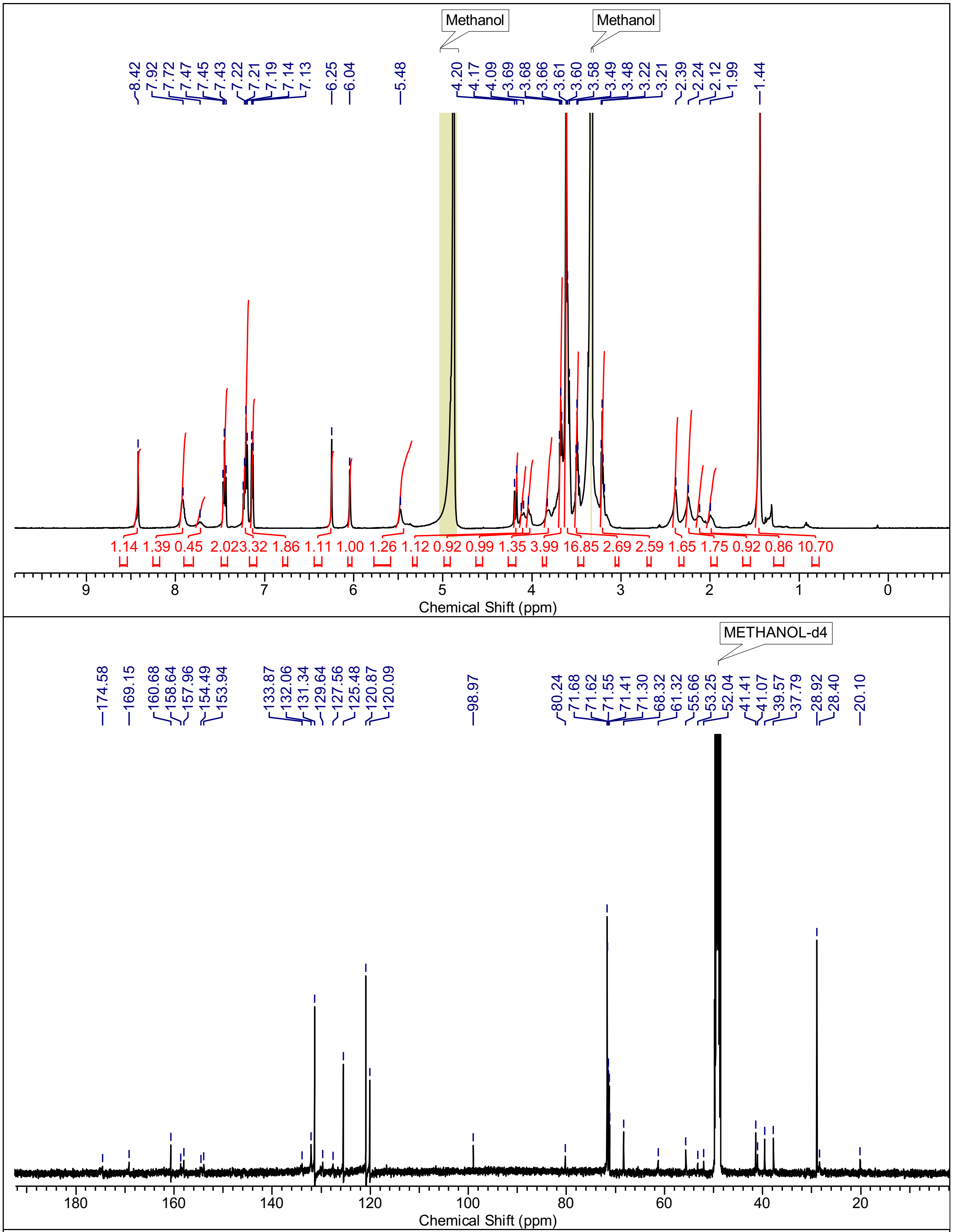

^{1}H and ^{13}C NMR spectra of **1m**

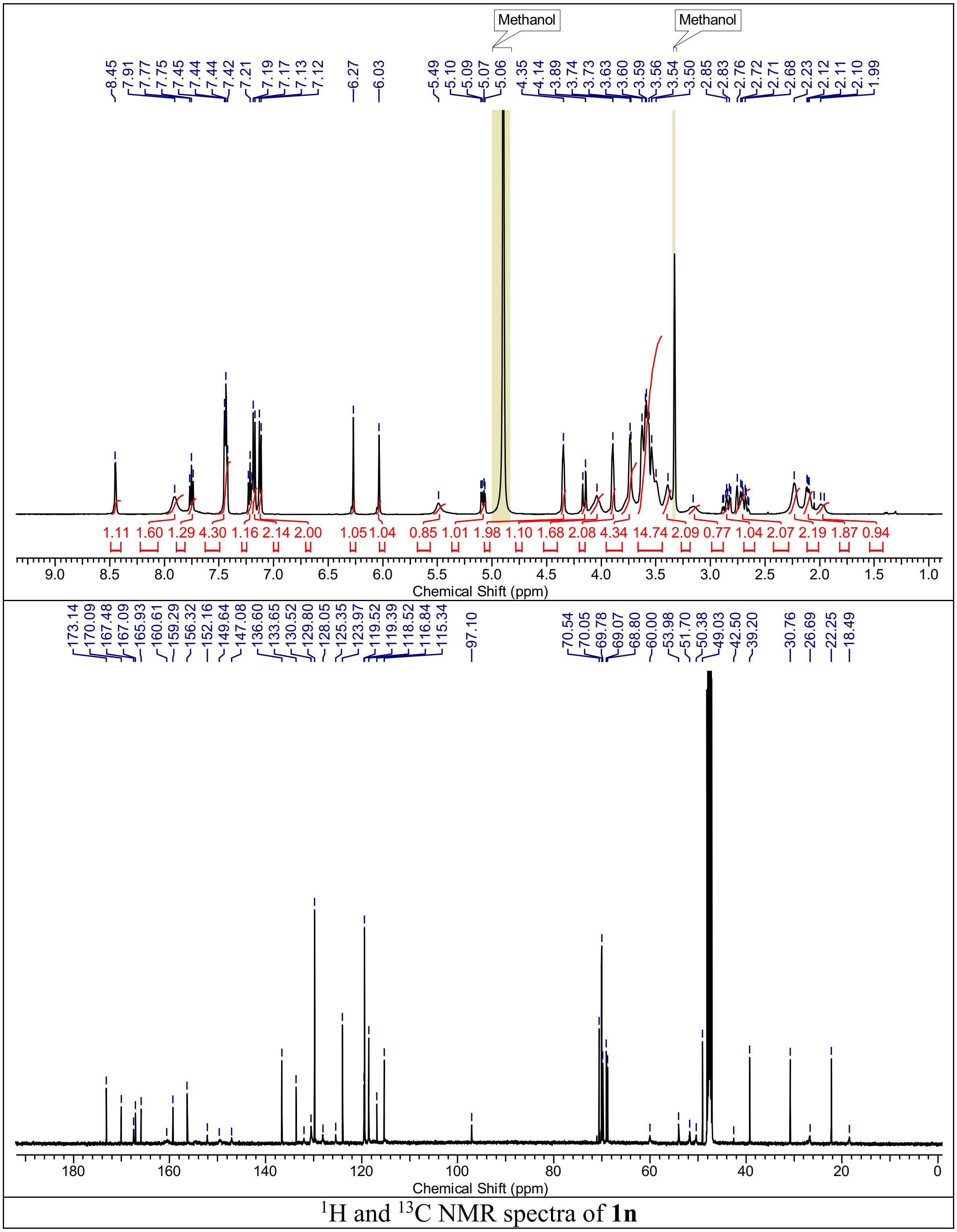

^{1}H and ^{13}C NMR spectra of **1n**

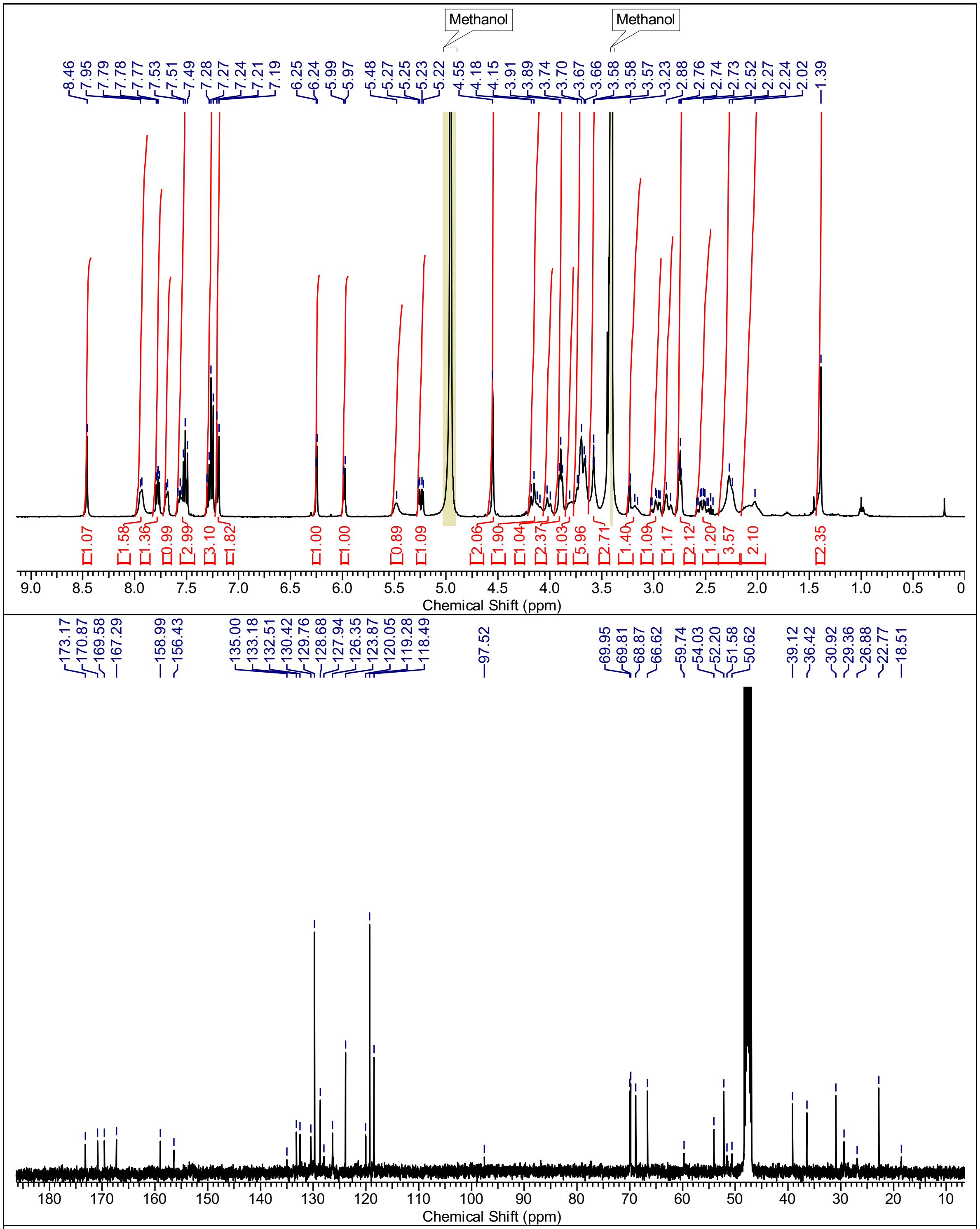

^{1}H and ^{13}C NMR spectra of **1o**

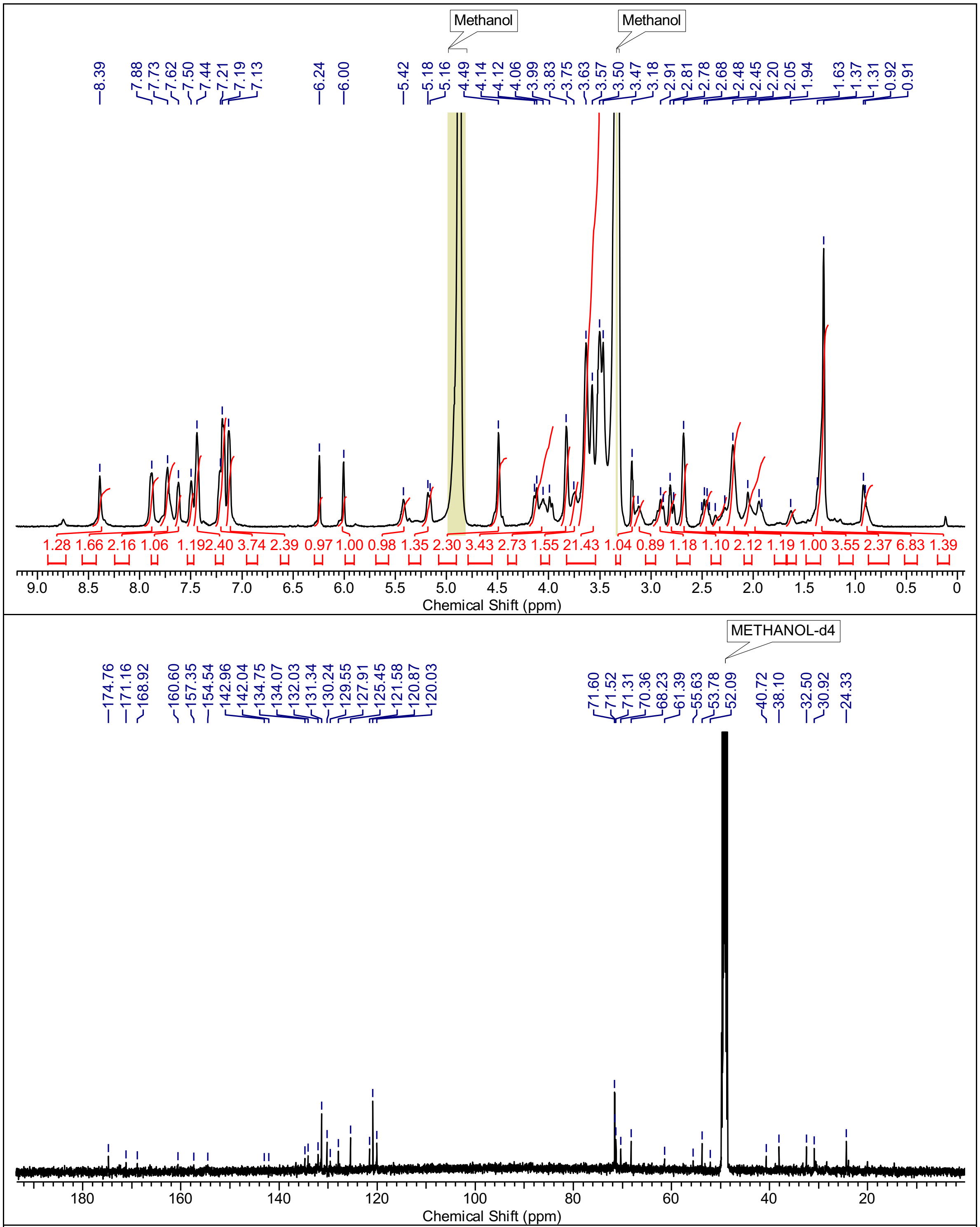

^{1}H and ^{13}C NMR spectra of **1p**

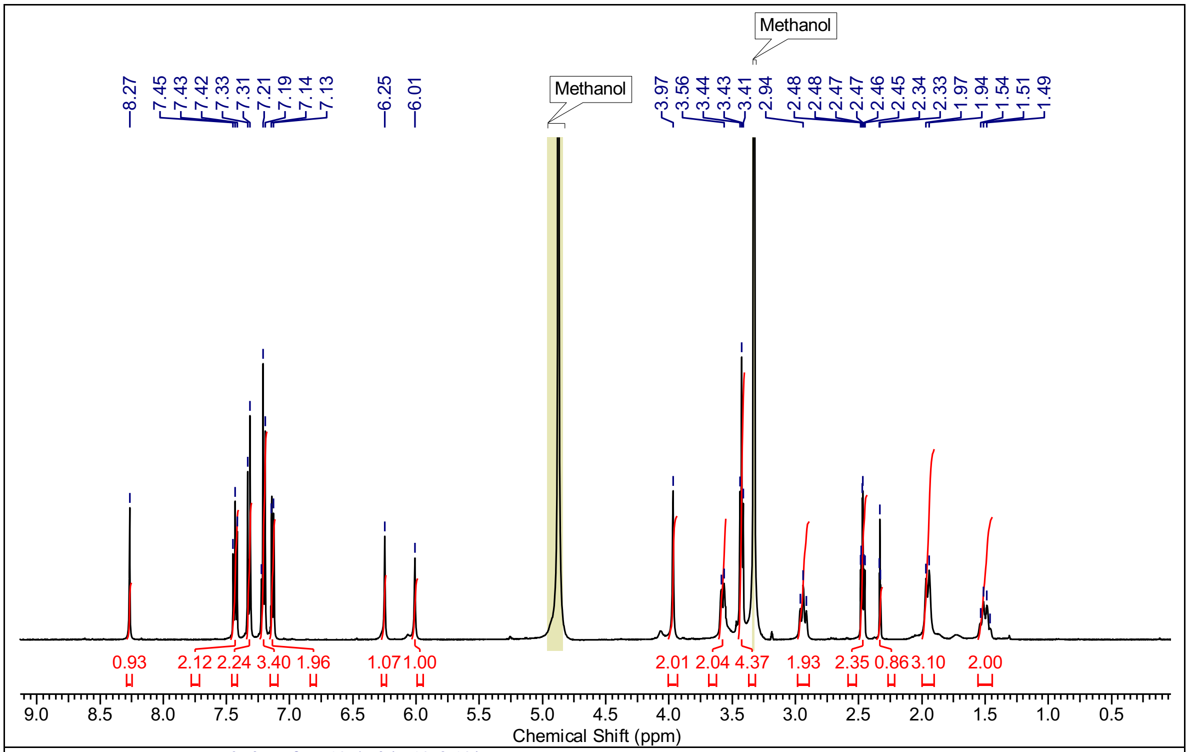

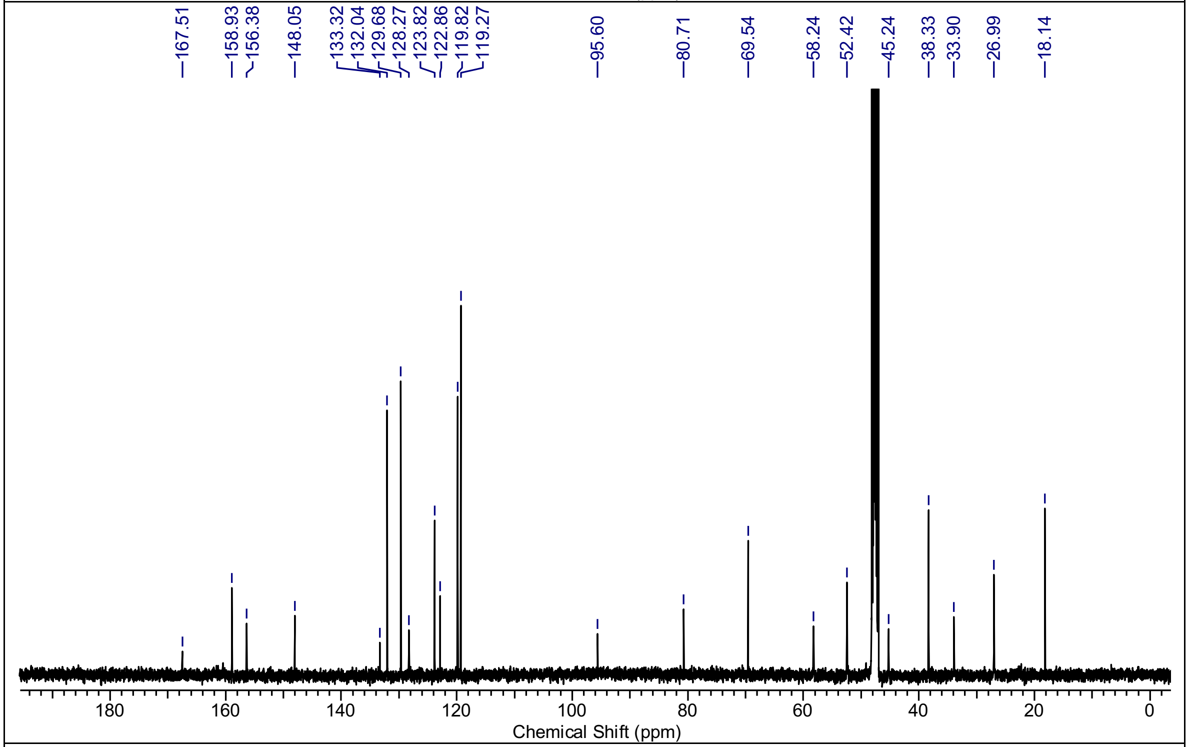

^{1}H and ^{13}C NMR spectra of **2a**

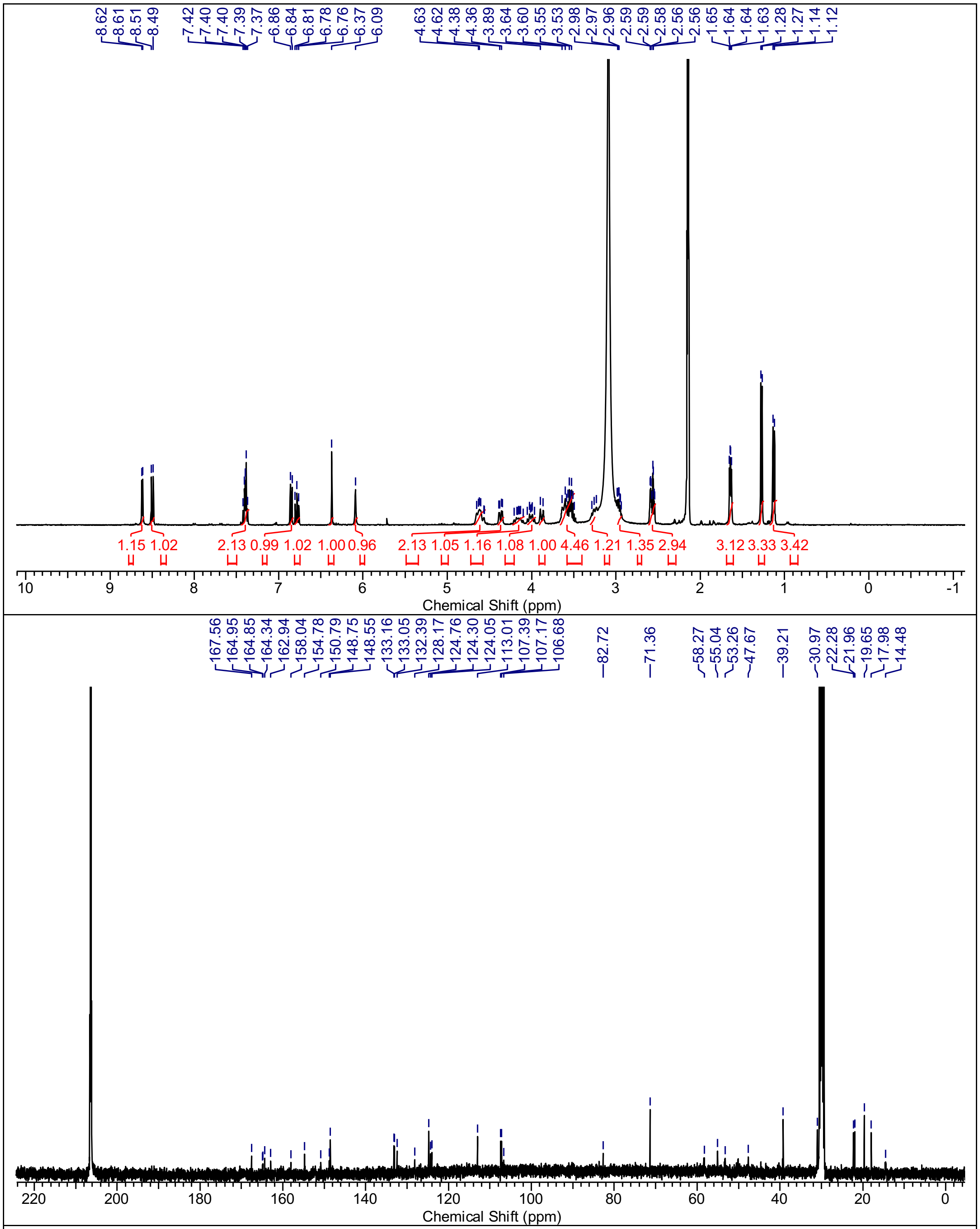

^{1}H and ^{13}C NMR spectra of **3a**

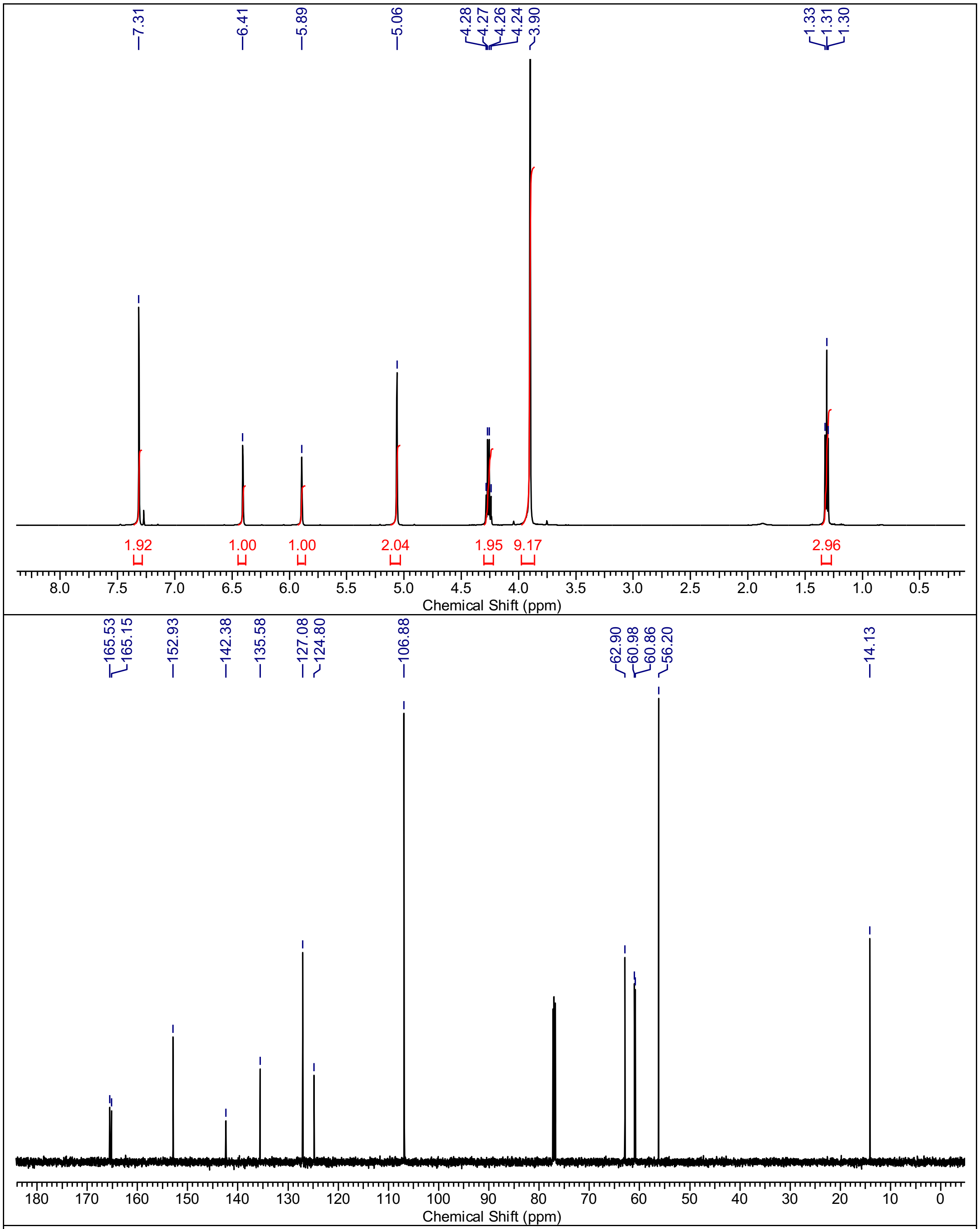

^{1}H and ^{13}C NMR spectra of **4a**
